# Neurophysiological effects of human-derived pathological tau conformers in the APPKM670/671NL.PS1/L166P amyloid mouse model of Alzheimer’s disease

**DOI:** 10.1038/s41598-022-11582-1

**Published:** 2022-05-11

**Authors:** S. Tok, H. Maurin, C. Delay, D. Crauwels, N. V. Manyakov, W. Van Der Elst, D. Moechars, W. H. I. M. Drinkenburg

**Affiliations:** 1 Department of Neuroscience, Janssen Research and Development, Janssen Pharmaceutica NV, Turnhoutseweg 30, 2340 Beerse, Belgium; 2 Data Sciences, Janssen Research and Development, Janssen Pharmaceutica NV, Turnhoutseweg 30, 2340 Beerse, Belgium; 3 Quantitative Sciences Janssen Research and Development, Janssen Pharmaceutica NV, Turnhoutseweg 30, 2340 Beerse, Belgium; 4 Faculty of Science and Engineering, Groningen Institute for Evolutionary Life Sciences, University of Groningen, Groningen, The Netherlands

**Keywords:** Diseases of the nervous system, Neural circuits

## Abstract

Alzheimer’s Disease (AD) is a neurodegenerative disease characterized by two main pathological hallmarks: amyloid plaques and intracellular tau neurofibrillary tangles. However, a majority of studies focus on the individual pathologies and seldom on the interaction between the two pathologies. Herein, we present the longitudinal neuropathological and neurophysiological effects of a combined amyloid-tau model by hippocampal seeding of human-derived tau pathology in the APP.PS1/L166P amyloid animal model. We statistically assessed both neurophysiological and pathological changes using linear mixed modelling to determine if factors such as the age at which animals were seeded, genotype, seeding or buffer, brain region where pathology was quantified, and time-post injection differentially affect these outcomes. We report that AT8-positive tau pathology progressively develops and is facilitated by the amount of amyloid pathology present at the time of injection. The amount of AT8-positive tau pathology was influenced by the interaction of age at which the animal was injected, genotype, and time after injection. Baseline pathology-related power spectra and Higuchi Fractal Dimension (HFD) score alterations were noted in APP.PS1/L166P before any manipulations were performed, indicating a baseline difference associated with genotype. We also report immediate localized hippocampal dysfunction in the electroencephalography (EEG) power spectra associated with tau seeding which returned to comparable levels at 1 month-post-injection. Longitudinal effects of seeding indicated that tau-seeded wild-type mice showed an increase in gamma power earlier than buffer control comparisons which was influenced by the age at which the animal was injected. A reduction of hippocampal broadband power spectra was noted in tau-seeded wild-type mice, but absent in APP.PS1 animals. HFD scores appeared to detect subtle effects associated with tau seeding in APP.PS1 animals, which was differentially influenced by genotype. Notably, while tau histopathological changes were present, a lack of overt longitudinal electrophysiological alterations was noted, particularly in APP.PS1 animals that feature both pathologies after seeding, reiterating and underscoring the difficulty and complexity associated with elucidating physiologically relevant and translatable biomarkers of Alzheimer’s Disease at the early stages of the disease.

## Introduction

Alzheimer’s Disease (AD) is a progressive neurodegenerative disorder generally characterized by two major histopathological hallmarks: extracellular amyloid plaques and intracellular tau aggregates which develop over the course of the disease^1,2^. However, the full understanding of the neurophysiological consequences of these pathologies in vivo have not been elucidated. Previous studies characterizing the neurophysiological effects of amyloid and tau pathology in animal models of AD pathology have identified several changes associated with alterations in theta-gamma coupling^3–6^, power spectrum changes^7–9^, presence of epileptiform activity^10,11^, network hyperactivity^12,13^ as well as tau-associated neuronal silencing^14,15^. However, many of these studies generally focus on either amyloid or tau pathology solely.

In order to model the effects of tau pathology in animals, one approach involves the injection of material capable of inducing the development of tau pathology; a method termed tau seeding^16^. A recent improvement to tau seeding published by Guo and colleagues^17^ involving the derivation of tau seeds from human brain material has demonstrated pathological conversion of endogenous tau in wild-type mice and is believed to confer a more physiological representation of AD-associated tau pathology. This is due to the fact that AD patients exhibit tau pathology that is not driven by mutations in the tau gene, unlike several animal models of tauopathies^18^. Subsequently, in a study by He and colleagues^19^, the pathological interaction between amyloid plaques and tau pathology was characterized and the finding of novel neuritic plaque tau pathology that was driving secondary tau conversion was reported. Clinical reports have also reported several neurophysiological changes associated with the progression of the disease^20^, such as: power spectra redistribution^21^, altered connectivity^22^, waveform complexity^23^ as well as epileptiform activity^24^. However, it is not clear what neurophysiological effects arise from the seeding of tau in this animal model. In addition, it is also unknown if any neurophysiological effects arise from the interaction between amyloid and tau pathology.

Our study was framed as an exploratory study to determine if and to what extent, neurophysiological effects associated with these amyloid and tau pathologies and their interactions would be. In this regard, we chose the APPKM670/671NL.PS1/L166P mouse model^25^ (subsequently referred to as the APP.PS1 mouse model in this paper), that start to develop amyloid pathology in the cortex at 6 weeks and in the hippocampus at 3–4 months of age, in order to investigate the synergistic effects of tau and amyloid pathology associated with the tau seeding method published by Guo and colleagues^17^. The primary neuropathological outcomes evaluated for this study were the presence, location, and amount of amyloid and tau pathology quantified via light sheet microscopy, and neurophysiological measures of local field potential (LFP) power spectra, phase-amplitude coupling, and Higuchi fractal dimension (HFD) complexity, which are also employed for clinical neurophysiological assessment of AD^21,23,26^, serving as a common ground for comparison.

We sought to identify neurophysiological changes associated with the onset and progression of AD-associated pathology in this exploratory study to determine if these readouts could be clinically relevant and translatable for detecting changes associated with the early phases of AD. Through the detection of these changes, a neurophysiological trajectory can be elucidated, which may aid in the identification of AD patient populations that may be at risk for or even be at the early stages of the disease. Thus, it is hoped that our findings from this study will eventually be useful for clinical assessment of AD from both a pathological and neurophysiological perspective.

## Results

### Age, genotype, treatment, and time-post injection interact and contribute to the development of AT8-positive tau pathology

**Figure 1:**
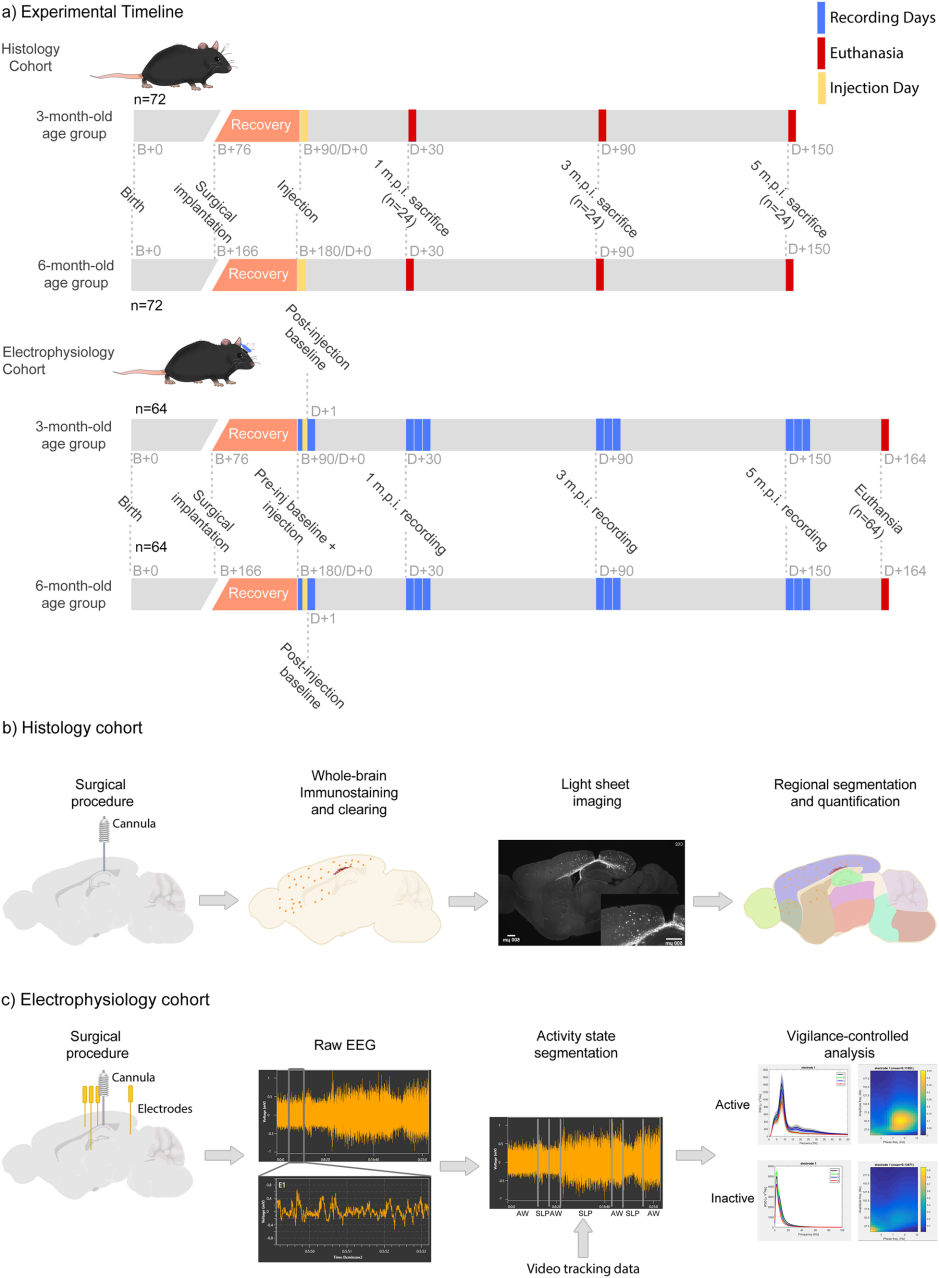
Overview of experimental design and analysis. (**a**) Visual timeline of experimental animal cohorts and initial number of animals in each cohort. Mice were instrumented with an electrophysiological montage and cannula or only a cannula at least 2 weeks prior to AD-tau seed injections. Mice were subsequently injected at 90 or 180 days after birth with AD-tau or buffer solutions and sacrificed or recorded at different timepoints (m.p.i. refers to months post injection). (**b**) Histological evaluation of pathology was carried out using brain clearance and immunohistological staining, followed by light-sheet imaging and image segmentation and quantification. (**c**) Sagittal illustration of the recording montage and analysis pipeline prior to quantitative analysis.

In order to understand the local and regional effects of tau pathology on neurophysiological outcomes, we first evaluated and characterized the spatiotemporal progression of tau pathology in APP.PS1 amyloid-bearing mice using AT8, a marker for phosphorylated tau^27^, correlated with the aggregation propensity of tau^28^. Pentameric formyl thiophene acetic acid (PFTAA), a marker for filamentous protein aggregates, was used to detect the presence of amyloid plaque pathology^29^. Aliquots of AD-Tau seeds prepared as described in the Methods section were thawed from -80 °C and used for injection into the hippocampus of mice. We measured the regional amount of AT8-positive tau and amyloid pathology (see Methods for more details) using light-sheet microscopy (LSM) images taken from animals sacrificed over a period of 5 months-post-injection (m.p.i.). This was carried out in 3- and 6-month-old animals in order to determine if the age at which injections occurred could influence outcomes (Fig. 1a). Heterozygous APP.PS1 transgenic (TG) and C57BL/6 wild-type (WT) animals (Fig. 1a) were injected with either AD-Tau seeds (i.e., TG-tau and WT-tau) or Buffer solution (i.e., TG-buffer and WT-buffer) via a cannula system (Fig. 1b). In addition, the neurophysiological effects of tau seeding were assessed in a separate electrophysiology cohort using electrodes implanted into the hippocampus, medial entorhinal cortex, retrosplenial cortex and thalamus (Fig. 1c).

**Figure 2:**
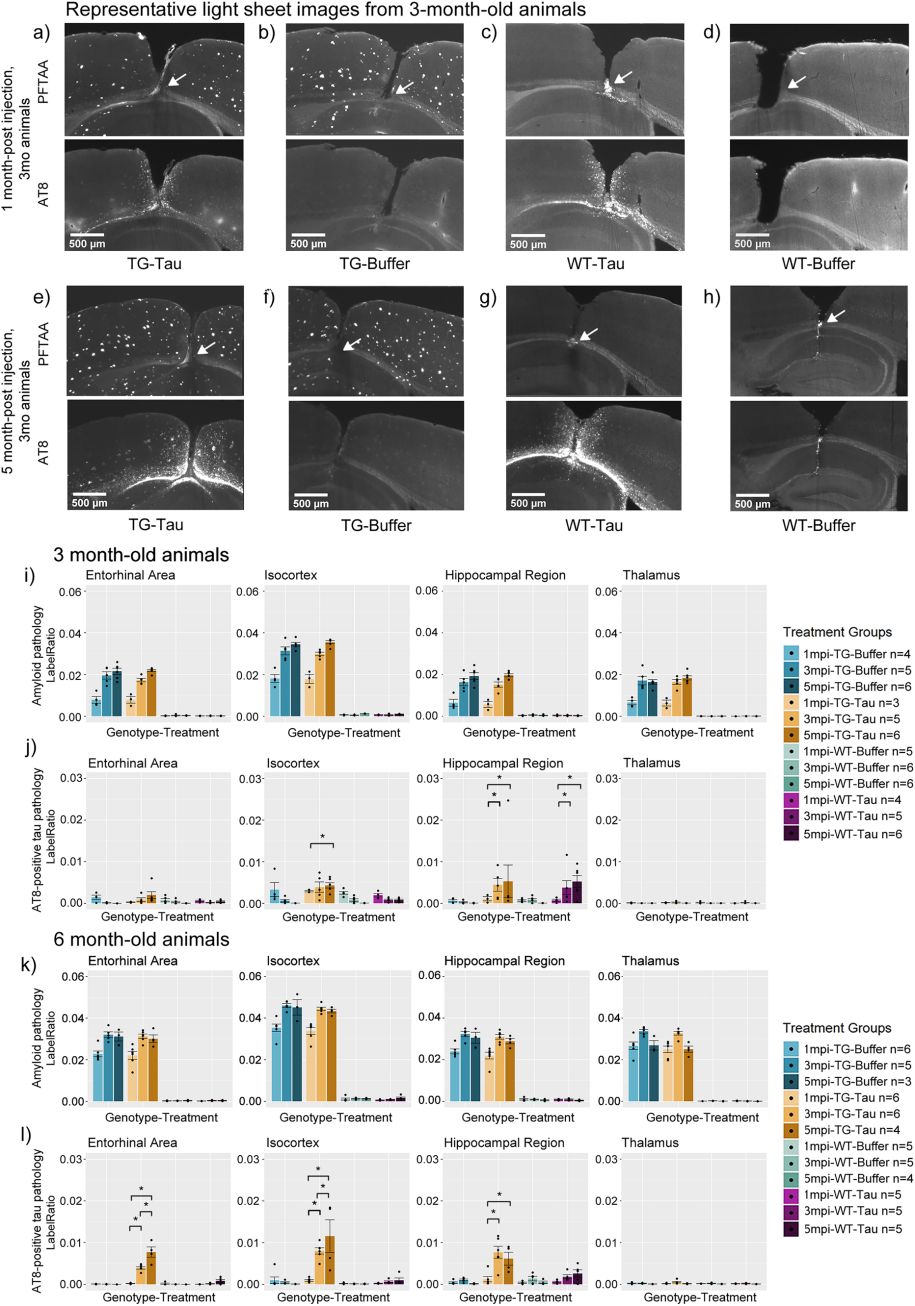
Representative histological images of APP.PS1 animals and wild-type littermates at 1- and 5- m.p.i. together with quantification of amyloid and tau pathology. (**a**)–(**d**) Representative histological images of 3-month-old APP.PS1 animals injected with (**a**) AD-tau or (**b**) Buffer, and wild type littermates injected with (**c**) AD-tau or (**d**) Buffer at 1 month-post injection. (**e**)–(**h**) Representative histological images of 3-month-old APP.PS1 animals injected with (**e**) AD-tau or (**f**) Buffer and wild type littermate injected with (**g**) AD-tau or (**h**) Buffer at 5 m.p.i. White arrows indicate site of injection. Bar plots of 3-month-old animals in terms of (**i**) Amyloid and (**j**) tau pathology across 5 months-post-injection. Bar plots of 6-month-old animals in terms of (**k**) Amyloid and (**l**) tau pathology across 5 months-post-injection. Significant comparisons across time indicate a progressive increase in AT8-positive tau pathology. (m.p.i. refers to months post injection). Asterisks indicate significant comparisons (*p* < 0.05). White arrows indicate the site of injection (i.e., the hippocampus).

TG mice exhibited amyloid deposition over the 5-month period regardless of injection type in 3-month-old animals (Fig. 2a,b). In contrast, WT animals did not exhibit amyloid plaque pathology (Fig. 2c,d). This was also the case after 5 months post-injection (Fig. 2e–h), seen in both 3-and 6-month-old animals (Fig. 2i,k). In line with the expected development of amyloid pathology, TG animals exhibited significant differences in the amount of amyloid pathology compared to WT animals (Supp. Fig. 1a,c, Supp. Table 1a). At 5 m.p.i., TG-tau and TG-Buffer animals did not exhibit significant differences in amyloid pathology in animals injected at either 3- or 6-months old, indicating that tau seeding did not significantly influence the development of the amount of amyloid pathology (Supp. Fig. 1b,d, Supp. Table 1b).

AT8-positive tau pathology was visible in the cortex overlying the injection site and near the injection site in both TG-tau (Fig. 2a) and WT-tau (Fig. 2c) animals at 1 m.p.i. and remained visible up till 5 m.p.i. in both TG-tau (Fig. 2e) and WT-tau (Fig. 2g) animals injected at either 3 or 6 month of age.

In order to understand which factors may be interacting and contributing to the development of either amyloid or tau pathology, a general linear mixed model (GLMM) was fit to the amount of AT8-positive tau pathology or amyloid pathology with Sex, Age at injection (referred to simply as Age in the model), Genotype, Treatment (seeded vs. buffer), Time post injection (also referred to as months-post-injection, or m.p.i.), and Brain region as fixed effects (main effects), and the Age x Genotype x Treatment x Time post injection x Brain region as interaction term (including all lower-order interactions that constitute this 5th-order interaction) (see Methods for more information).

For AT8 pathology, the Age x Genotype x Treatment x Time post injection x Brain region interaction term was not significant (using alpha = 0.05), (i.e., ${\chi }^{2}\left(6\right)=$ 4.323035, *p* = 0.633). After excluding the highest order interaction term and refitting the model, the significance of the lower-order interactions terms was tested. Subsequently, the Brain region x Genotype x Treatment x Time post injection interaction was statistically significant (${\chi }^{2}\left(6\right)=17.08624,$ *p*-value = 0.009), as well as the Age x Genotype x Treatment x Time-post-injection interaction (${\chi }^{2}\left(2\right)=6.182092$, *p*-value = 0.0455).

For amyloid pathology, The Age x Genotype x Treatment x Time post injection x Brain region interaction term was again not significant, (i.e., ${\chi }^{2}(6)$= 0.7491047, *p*-value = 0.9934). After excluding the highest order interaction term and refitting the model, the significance of the lower-order interactions terms was tested. There was no significant interaction that included Treatment, suggesting that amyloid pathology was not significantly affected by the tau seeding based on this data.

Subsequently, post-hoc contrasts were generated from the fitted models (i.e., using predicted marginal means) and adjusted using Benjamini–Hochberg false discovery rate (FDR) for the measures (Refer to Statistical Analysis and Supplementary Methods for more information). The interaction effects are investigated in more detail in the next sections.

### AT8-positive tau pathology develops longitudinally and proximal to the injection site in APP.PS1 mice following AD-tau injection in 3-month-old and 6-month-old animals

The injection of AD-tau seeds induced significant AT8-positive tau pathology in both WT and TG animals. TG-tau animals exhibited significantly more AT8 pathology in all quantified regions except the thalamus at 5 m.p.i. for both age groups (Supp. Fig. 2a,b, Supp. Table 2a) when compared to buffer-treated animals. In the case of WT-tau animals, significantly more AT8 pathology in the hippocampi of both age groups (Supp. Fig. 2a,b, Supp. Table 2a), at 5 m.p.i. was noted but not in other areas. Animals injected with buffer solution did not exhibit AT8 positive tau pathology in either TG-buffer (Fig. 2b) or WT-buffer (Fig. 2d) animals at 1 m.p.i. or at 5 m.p.i. (Fig. 2f, h). AT8-positive tau pathology was noted to be localised to the site near the site of injection (Fig. 2e, g). These results indicate that the injection of AD-tau seeds result in the development of AT8-positive tau pathology regardless of genotype.

AT8-positive pathology was also noted to develop longitudinally in 3- and 6-month-old TG and WT animals injected with AD-tau seeds (Fig. 2e, g, j, l), albeit to a much lesser extent in WT animals as seen in histological images and bar plots. Subsequently, we statistically compared AT8-positive tau pathology between animals at different timepoints to determine if tau pathology developed over time. Tau-injected animals from both 3- and 6-month groups showed significantly increased AT8-positive tau pathology at later timepoints compared to earlier timepoints (Fig. 2j, l, Table 1a). 6-month-old animals showed significant increases in AT8 pathology in the entorhinal, isocortex, and hippocampus at 3- and 5-m.p.i. when compared to 1 m.p.i. (Fig. 2l, Table 1a). However, 3-month-old TG-tau mice only showed a significant increase in AT8 pathology in the isocortex when comparing 5 m.p.i. to 1 m.p.i., and in the hippocampus (Fig. 2j, Table 1a), which could indicate a slower rate of development. No significant differences were noted in the Thalamus of either 3- or 6-month-old TG-tau mice at any timepoints, likely due to the distance from the injection site.

**Table 1:** Table containing the pairwise comparisons of the amount of quantified AT8-positive tau pathology.

Brain Region	Pairwise comparison	Estimate	SE	df	T ratio	*P* value
**(a) Longitudinal comparison of AT8 pathology**						
Entorhinal area	6mo TG-Tau 1mpi—6mo TG-Tau 3mpi	− 0.0037	0.0010	96	− 3.7452	0.0021
	6mo TG-Tau 1mpi—6mo TG-Tau 5mpi	− 0.0075	0.0011	96	− 6.7372	0.0001
	6mo TG-Tau 3mpi—6mo TG-Tau 5mpi	− 0.0038	0.0011	96	− 3.3899	0.0056
Isocortex	3mo TG-Tau 1mpi—3mo TG-Tau 5mpi	0.0032	0.0011	96	2.9076	0.0216
	6mo TG-Tau 1mpi—6mo TG-Tau 3mpi	− 0.0071	0.0010	96	− 7.0609	0.0001
	6mo TG-Tau 3mpi—6mo TG-Tau 5mpi	− 0.0035	0.0011	96	− 3.1687	0.0106
	6mo TG-Tau 1mpi—6mo TG-Tau 5mpi	− 0.0106	0.0011	96	− 9.4793	0.0000
Hippocampal region	3mo TG-Tau 1mpi—3mo TG-Tau 3mpi	− 0.0033	0.0013	96	− 2.6427	0.0433
	3mo TG-Tau 1mpi—3mo TG-Tau 5mpi	− 0.0042	0.0012	96	− 3.4451	0.0048
	6mo TG-Tau 1mpi—6mo TG-Tau 3mpi	− 0.0065	0.0010	96	− 6.4639	0.0001
	6mo TG-Tau 1mpi—6mo TG-Tau 5mpi	− 0.0049	0.0011	96	− 4.4162	0.0002

Brain region	Pairwise comparison	Estimate	SE	df	T ratio	*P* value
**(b) Genotype comparison of AT8 pathology**						
Entorhinal area	3mo TG-Tau 5mpi—3mo WT Tau 5mpi	0.0016	0.0010	96	1.5568	0.3969
	6mo TG-Tau 5mpi—6mo WT Tau 5mpi	0.0069	0.0012	96	5.9365	0.0001
Isocortex	3mo TG-Tau 5mpi—3mo WT Tau 5mpi	0.0035	0.0010	96	3.5311	0.0039
	6mo TG-Tau 5mpi—6mo WT Tau 5mpi	0.0106	0.0012	96	9.0844	0.0000
Hippocampal region	3mo TG-Tau 5mpi—3mo WT Tau 5mpi	0.0001	0.0010	96	0.0649	0.9999
	6mo TG-Tau 5mpi—6mo WT Tau 5mpi	0.0036	0.0012	96	3.0635	0.0143

Brain region	Pairwise comparison	Estimate	SE	df	T ratio	*P* value
**(c) Age comparison of AT8 pathology**						
Entorhinal area	3mo TG-Tau 3mpi—6mo TG-Tau 3mpi	− 0.0038	0.0010	96	− 3.6673	0.0026
	3mo TG-Tau 5mpi—6mo TG-Tau 5mpi	− 0.0057	0.0011	96	− 5.1333	0.0000
Isocortex	3mo TG-Tau 3mpi—6mo TG-Tau 3mpi	− 0.0041	0.0010	96	− 3.9257	0.0012
	3mo TG-Tau 5mpi—6mo TG-Tau 5mpi	− 0.0072	0.0011	96	− 6.4584	0.0000

Pairwise comparisons between (a) Between timepoints in the same genotype-treatment group, (b) Between genotypes, and (c) Between different age groups. 3mo and 6mo refer to 3- or 6-months old animals. (m.p.i. refers to months post injection). TG refers to APP.PS1 animals, WT refers to wild-type. Tau refers to tau-seeded mice, Buffer refers to phosphate-buffered saline injected mice. SE refers to standard error. Df refers to degrees of freedom. Estimate refers to the estimated difference in value between pairwise comparisons. mo refers to months-old at injection.

### Amyloid pathology facilitates the development of AT8 pathology

Next, we sought to understand the role of amyloid plaque pathology in facilitating the progression of tau pathology. Previous reports^17,19^ and computational modelling^30^ have indicated a potential role of amyloid promoting the development and progression of tau pathology. Also in this regard, the significant interaction effect containing factors of Genotype and Age, implies that animals with more amyloid pathology (i.e., aged APP.PS1 animals) may develop AT8-positive tau pathology differently compared to animals with less amyloid pathology (i.e., younger APP.PS1 animals or WT animals).

**Figure 3:**
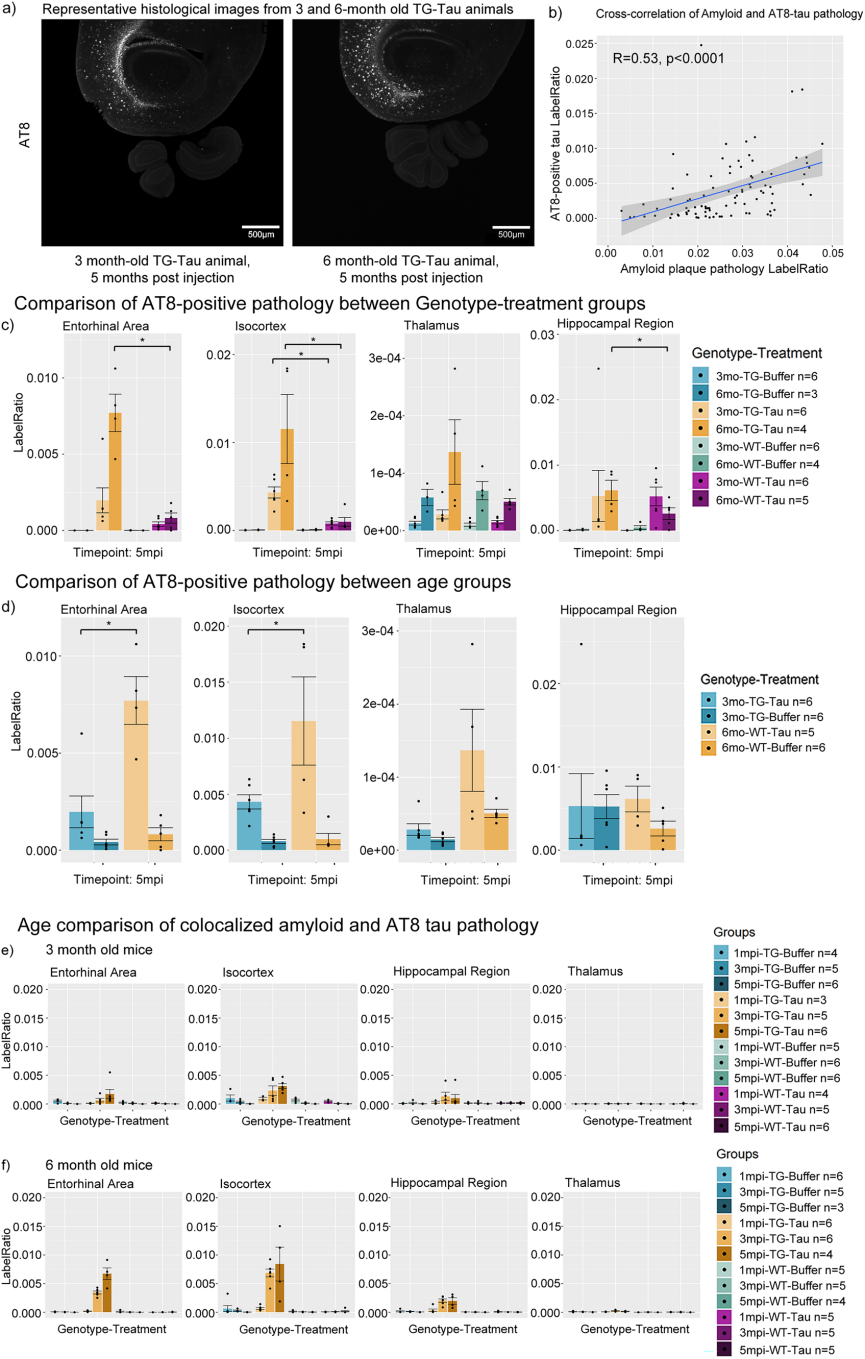
(**a**) Representative images of AT8 pathology at 5 m.p.i. in mice injected with AD-tau seeds at 3 and 6 months of age. (**b**) Cross-correlation of amyloid and tau pathology pooling the hippocampal region, isocortex and entorhinal area at both 3 and 6 months of age (R = 0.53, *p* < 0.0001). (**c**) Bar plots comparisons of AT8-positive tau pathology between genotype-treatment groups at 5 months post injection. APP.PS1 animals exhibit significantly more AT8-positive tau pathology than wild-type animals in the isocortex and entorhinal cortex. (**d**) Comparison of AT8-positive tau pathology in APP.PS1 animals between age groups at 5 months post injection. Older APP.PS1 animals exhibit significantly more AT8 pathology compared to younger animals when controlling for same time post-injection. (**e**) Bar plot comparisons of AT8-positive tau pathology colocalized with amyloid pathology in 3-month-old animals, and (**f**) 6-month-old animals. (m.p.i. refers to months post injection). Asterisks indicate significant comparisons (*p* < 0.05).

To better understand this interaction effect, first we qualitatively compared 6-month-old TG-tau to 3-month-old TG-tau animals (Fig. 3a) as seen from representative LSM images, which showed that older TG animals demonstrated more AT8-positive tau pathology, even at the same time post injection.

As a cursory visualization of this relationship, we performed a direct correlation of the amount of measured amyloid plaque and AT8-positive tau pathology. A Pearson correlation was computed between the amount of amyloid pathology and AT8-positive tau pathology, considering only tau-injected animals, pooling animals from different ages, time-post-injection, sex, brain region and genotype. A significant moderate correlation between the amount of amyloid and tau pathology was present in the data (R = 0.53, *p* < 0.0001) (Fig. 3b).

Next, we sought to quantify and statistically evaluate the interaction described above. To understand the effect of genotype on the development of AT8-positive tau pathology, we statistically compared TG-tau and WT-tau animals at the same ages and timepoints. Significant increases were found in AT8-positive tau pathology in TG mice compared to similarly aged and treated WT mice in the hippocampal region, isocortex and entorhinal cortex of 6-month-old animals, and isocortex of 3-month-old animals (Fig. 3c, Table 1b), indicating that when accounting for other factors, TG-tau mice develop more AT8-positive tau pathology than WT-tau mice after seeding.

The relationship between age and AT8-positive tau pathology was investigated by quantitatively comparing older and younger TG-tau animals when controlling for the same time post injection. Significantly more AT8-positive tau pathology was found in aged mice compared to younger TG-tau mice at 3 m.p.i. (Table 1c) and 5 m.p.i. (Fig. 3d, Table 1c) in the Entorhinal cortex and Isocortex, suggesting that more AT8-positive tau pathology may be developing in older animals compared to younger ones. Interestingly, this relationship was not noted in the hippocampus (i.e., no significant difference in between older and younger animals) suggesting that amyloid plaque load could be a factor influencing the development of AT8-positive tau pathology.

From the representative light sheet images (Fig. 3a), the distribution pattern of AT8-positive tau pathology appeared to be localized near amyloid plaque pathology, suggesting a possible affinity of AT8-positive tau pathology for amyloid plaques. Thus, we sought to understand and quantify the prevalence of AT8 pathology near plaques (Fig. 3e and f) (see Methods for defining colocalized tau pathology). This result was modelled with a GLMM and fit with the same interaction terms as for individual pathologies. An example of the plaque-associated AT8-positive tau pathology can be found in (Supp. Fig. 4b, white arrow), as well as non-associated AT8-positive tau pathology (Supp. Fig. 4b, grey arrow). The interaction between Age x Genotype x Treatment x Time post injection x Brain region was significant (i.e., ${\chi }^{2}(6)$= 16.24371, *p*-value = 0.0125), suggesting that the amount of colocalized AT8 pathology develops differently, based on the age at which the animal was injected and genotype of the injected animal. In addition, the regions do not develop colocalized pathology in the same way, and that it develops differently across time-post-injection.

Taken together, these data strongly indicate that the progression and development of AT8 is dependent on the interplay (i.e., the interaction) of the factors of age, genotype, and time post-injection, which correlate with the presence and amount of amyloid pathology.

### Significant interactions between age, treatment, genotype, timepoints and electrodes for neurophysiological readouts were not present in the data

As a follow up to the significant interactions observed in histological experiments, we sought to understand if this interaction effect might be reflected in neurophysiological readouts. One main hypothesis of interest to the field is understanding how pathology influences the neurophysiological and electrophysiological properties of the brain (i.e., does the amount or presence of pathology correspond to a change in a neurophysiological readout?).

Thus, we implanted electrodes in the hippocampus (HPC), thalamus (Tha), medial entorhinal cortex (MEC) and retrosplenial cortex (RSC) (see Methods for more details) in a separate cohort of identically aged and injected APP.PS1 and wild-type mice. We recorded the LFPs of these animals at timepoints that corresponded to the measurement of pathologies in the histological cohort (i.e., 1, 3 and 5 m.p.i.), as well as baselines before and after injection. Subsequently the power spectra density, phase-amplitude coupling modulation indices, as well as HFD complexity scores were endpoints for evaluating changes in neurophysiology associated with pathology.

To determine if significant interactions were present, we fit a GLMM with Sex, Age at injection, Genotype, Treatment (seeded vs. buffer), Time post injection and Electrode location (i.e., brain region) as fixed effects (main effects), and the Age x Genotype x Treatment x Time post injection x Electrode location as interaction term (including all lower-order interactions that constitute this 5th-order interaction). Individual GLMMs for all neurophysiological readouts of power spectra measurements, including sub-band power, phase-amplitude coupling and all sub-coupling pairs, as well as HFD scores were constructed and tested to determine if significant interactions were present (see Methods for more details).

The Age x Genotype x Treatment x Time post injection x Electrode interaction was not significant in any of the readouts. After excluding the highest order interaction term and refitting the model, the significance of the lower-order interactions terms was tested. Significant lower-order interactions were noted for the outcomes of theta-2, low gamma and high gamma power and fractal dimension scores, suggesting an interaction effect of these independent variables but no significant interaction of a time-post-injection factor that was detected using a linear model.

From the power spectra measures, theta 2 (i.e., ${\chi }^{2}(3)$= 24.90998, *p*-value < 0.0001), low gamma (i.e., ${\chi }^{2}(3)$= 43.1246, *p*-value < 0.0001) and high gamma power (i.e., ${\chi }^{2}(3)$= 44.2878, *p*-value < 0.0001), significant interactions between Age, Electrode, Genotype and Treatment was noted. These interactions indicate that the effects of tau seeding on theta and gamma powers are affected by the age at which the animal was injected, genotype, and that this effect is different across electrodes.

For readouts of phase-amplitude coupling, no significant interactions of interest were noted in the dataset.

For HFD measures, a significant interaction between Age, Electrode, Genotype and Treatment was noted (i.e., ${\chi }^{2}(3)$= 39.09707, *p*-value < 0.0001). Similar to the readouts of power spectra, these interactions indicate that the effects of tau seeding on HFD scores are affected by the age at which the animal was injected, genotype, and that this effect differs by brain region.

Notably, none of these interactions included a time-post-injection factor, indicating that there was no significant longitudinal effect of the tau seeding.

From these linear models, pairwise contrasts were generated and adjusted using Benjamini–Hochberg FDR for each of the sub-measures of power spectra and fractal dimension. The results of the significant interactions are described in the next sections.

### APP.PS1 animals exhibit altered power spectra and fractal dimension baselines prior to injections

**Figure 4:**
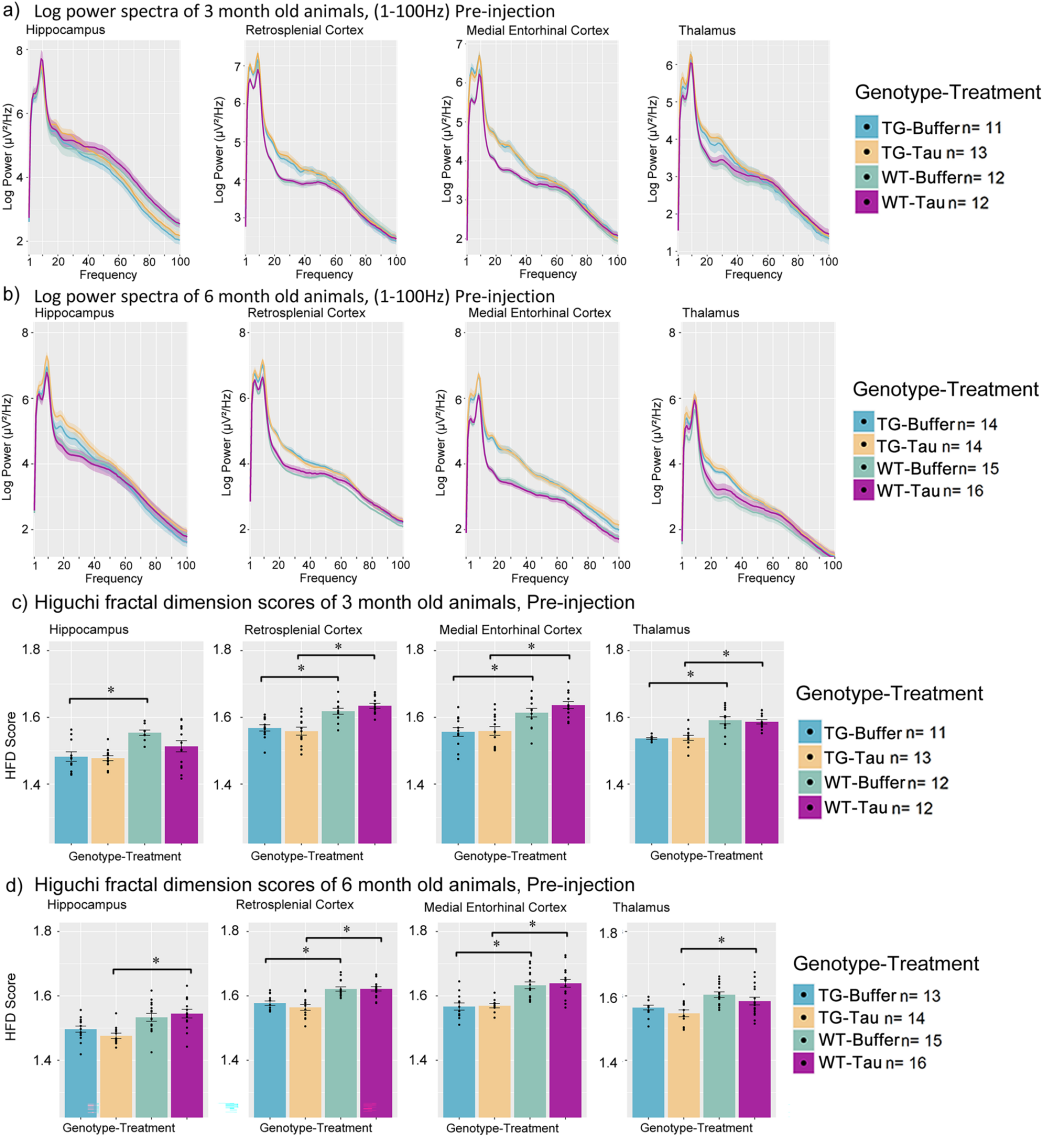
Power spectrum and fractal dimension baseline measures of 3-month-old APP.PS1 and wild-type animals across the hippocampus, retrosplenial cortex, medial entorhinal cortex and thalamus. Log power spectra of APP.PS1 animals and wild-type animals from 1-100 Hz at Pre-injection for (**a**) 3-month-old and (**b**) 6-month-old animals. Bar plots of fractal dimension scores of (**c**) 3-month-old and (**d**) 6-month-old animals at Pre-injection. (m.p.i. refers to months post injection). All bar plots are Mean + SEM. Asterisks indicate significant comparisons (*p* < 0.05).

In TG animals, EEG power was substantially increased in the RSC, MEC and Tha compared to WT animals prior to any injections. Qualitative indications of increased power of TG animals were observed from 1 to 100 Hz power spectra graph in 3-month-old animals (Fig. 4a), as well as 6-month-old animals (Fig. 4b), most notably in the RSC, MEC and Tha electrodes.

To understand which frequency bands contribute to the increased power, power of frequency bands of Delta, Theta-1, Theta-2, Low Gamma and High Gamma were quantified (see Methods for more details on the frequency definitions for each band). TG animals exhibited a consistent significant increase most notably in Theta-2 band power in the RSC and MEC and Delta, Theta-1 for the MEC (refer to Table 2a for a summary of significant pairwise comparisons for each of the specific bands for each region). No consistent difference was detected in these frequency bands for the Tha electrode.

**Table 2:** Table containing the pairwise comparisons of quantified power spectra values and Higuchi fractal dimension values of animals at baseline conditions.

Frequency band	Pairwise comparison	Estimate	SE	df	T ratio	*P* value
**(a) Baseline power spectrum comparisons**						
Delta	3mo MEC Tg Buffer Pre—3mo MEC Wt Buffer Pre	0.7118	0.2206	112	3.2265	0.0035
	3mo MEC Tg Tau Pre—3mo MEC Wt Tau Pre	0.6326	0.2089	112	3.0283	0.0061
	6mo MEC Tg Buffer Pre—6mo MEC Wt Buffer Pre	0.5232	0.2084	112	2.5109	0.0238
	6mo MEC Tg Tau Pre—6mo MEC Wt Tau Pre	0.6651	0.2074	112	3.2066	0.0037
Theta1	3mo MEC Tg Buffer Pre—3mo MEC Wt Buffer Pre	0.8670	0.2153	112	4.0273	0.0002
	3mo MEC Tg Tau Pre—3mo MEC Wt Tau Pre	0.6644	0.2039	112	3.2579	0.0029
	6mo MEC Tg Buffer Pre—6mo MEC Wt Buffer Pre	0.7321	0.2036	112	3.5966	0.0010
	6mo MEC Tg Tau Pre—6mo MEC Wt Tau Pre	0.7920	0.2025	112	3.9108	0.0004
Theta2	3mo RSC Tg Tau Pre—3mo RSC Wt Tau Pre	0.4348	0.1959	112	2.2196	0.0438
	6mo RSC Tg Buffer Pre—6mo RSC Wt Buffer Pre	0.4166	0.1917	112	2.1729	0.0487
	3mo MEC Tg Buffer Pre—3mo MEC Wt Buffer Pre	0.7177	0.2068	112	3.4701	0.0015
	3mo MEC Tg Tau Pre—3mo MEC Wt Tau Pre	0.6066	0.1959	112	3.0967	0.0045
	6mo MEC Tg Buffer Pre—6mo MEC Wt Buffer Pre	0.6828	0.1954	112	3.4940	0.0014
	6mo MEC Tg Tau Pre—6mo MEC Wt Tau Pre	0.7342	0.1945	112	3.7754	0.0005
Low Gamma	3mo RSC Tg Tau Pre—3mo RSC Wt Tau Pre	0.4066	0.1794	112	2.2660	0.0380
	6mo RSC Tg Buffer Pre—6mo RSC Wt Buffer Pre	0.4220	0.1754	112	2.4065	0.0273
	3mo MEC Tg Buffer Pre—3mo MEC Wt Buffer Pre	0.4541	0.1895	112	2.3958	0.0281
	6mo MEC Tg Buffer Pre—6mo MEC Wt Buffer Pre	0.6605	0.1789	112	3.6921	0.0007
	6mo MEC Tg Tau Pre—6mo MEC Wt Tau Pre	0.6927	0.1781	112	3.8896	0.0004
High Gamma	6mo MEC Tg Tau Pre—6mo MEC Wt Tau Pre	0.4132	0.1635	112	2.5273	0.0241

HFD comparisons	Pairwise comparison	Estimate	SE	df	T ratio	*P* value
(b) Baseline fractal dimension analysis						
	3mo HPC Tg Buffer Pre—3mo HPC Wt Buffer Pre	–.0672	0.0201	112	–3.3355	0.0024
	6mo HPC Tg Tau Pre—6mo HPC Wt Tau Pre	–.0588	0.0184	112	–3.1858	0.0037
	3mo RSC Tg Buffer Pre—3mo RSC Wt Buffer Pre	–.0581	0.0195	112	–2.9793	0.0067
	3mo RSC Tg Tau Pre—3mo RSC Wt Tau Pre	–.0612	0.0185	112	–3.3128	0.0026
	6mo RSC Tg Buffer Pre—6mo RSC Wt Buffer Pre	–.0505	0.0181	112	–2.7950	0.0110
	6mo RSC Tg Tau Pre—6mo RSC Wt Tau Pre	–.0597	0.0180	112	–3.3122	0.0026
	3mo MEC Tg Buffer Pre—3mo MEC Wt Buffer Pre	–.0656	0.0195	112	–3.3640	0.0022
	3mo MEC Tg Tau Pre—3mo MEC Wt Tau Pre	–.0771	0.0185	112	–4.1767	0.0002
	6mo MEC Tg Buffer Pre—6mo MEC Wt Buffer Pre	–.0650	0.0184	112	–3.5280	0.0013
	6mo MEC Tg Tau Pre—6mo MEC Wt Tau Pre	–.0758	0.0183	112	–4.1354	0.0002
	3mo Tha Tg Buffer Pre—3mo Tha Wt Buffer Pre	–.0683	0.0198	112	–3.4479	0.0017
	3mo Tha Tg Tau Pre—3mo Tha Wt Tau Pre	–.0595	0.0185	112	–3.2222	0.0033
	6mo Tha Tg Tau Pre—6mo Tha Wt Tau Pre	–.0394	0.0178	112	–2.2183	0.0445

Pairwise comparisons between (a) power spectra band values and (b) fractal dimension between APP.PS1 animals and wild-type animals prior to injection. 3mo and 6mo refer to 3- or 6-months old animals. Pre refers to Pre-injection timepoint. MEC refers to Medial Entorhinal Cortex, HPC refers to Hippocampal CA1, Tha refers to thalamus, RSC refers to Retrosplenial Cortex. HFD refers to Higuchi fractal dimension. TG refers to APP.PS1 animals, WT refers to wild-type. Tau refers to tau-seeded mice, Buffer refers to phosphate-buffered saline injected mice. SE refers to standard error. Df refers to degrees of freedom. Estimate refers to the estimated difference in value between pairwise comparisons. mo refers to months-old at injection.

TG animals also exhibited significantly decreased HFD scores in the RSC, MEC, as well as the Tha at 3 months of age (Fig. 4c, Table 2b) and in the MEC and RSC when compared to WT controls. 6-month-old animals exhibited significantly reduced HFD scores in the RSC and MEC but not consistently in the HPC nor Tha (Fig. 4d, Table 2b) when compared to age-matched WT controls.

Measures of phase-amplitude coupling were not noted to be significantly different at baseline within any of the sub-measures, suggesting comparable phase-amplitude coupling modulation indices in both TG and WT animals at 3 and 6 months of age prior to injection.

These data suggest that APP.PS1 animals exhibit marked increases in power spectra in the MEC, RSC, and Tha, but not in the hippocampus, and reduced HFD scores at baseline when compared to WT animals, but do not exhibit significantly different phase-amplitude coupling scores in our current study, likely reflecting the effect of amyloid pathology.

### AD-tau injected animals immediately exhibit decreases in theta and gamma power in both genotypes but return to comparable levels at 1 month post injection

Next, we attempted to characterize the immediate effects of the injection in both TG and WT animals. Prior findings from Busche et al. as well as Marinkovic et al.^14,15^ have suggested the effects of soluble tau to possess a silencing effect on neuronal activity resulting from transgenic manipulations of tau. Thus, we hypothesized the injection of AD-tau seeds and subsequent development of pathology could result in similar effects of neuronal silencing and thus, reduced power spectra values.

**Figure 5:**
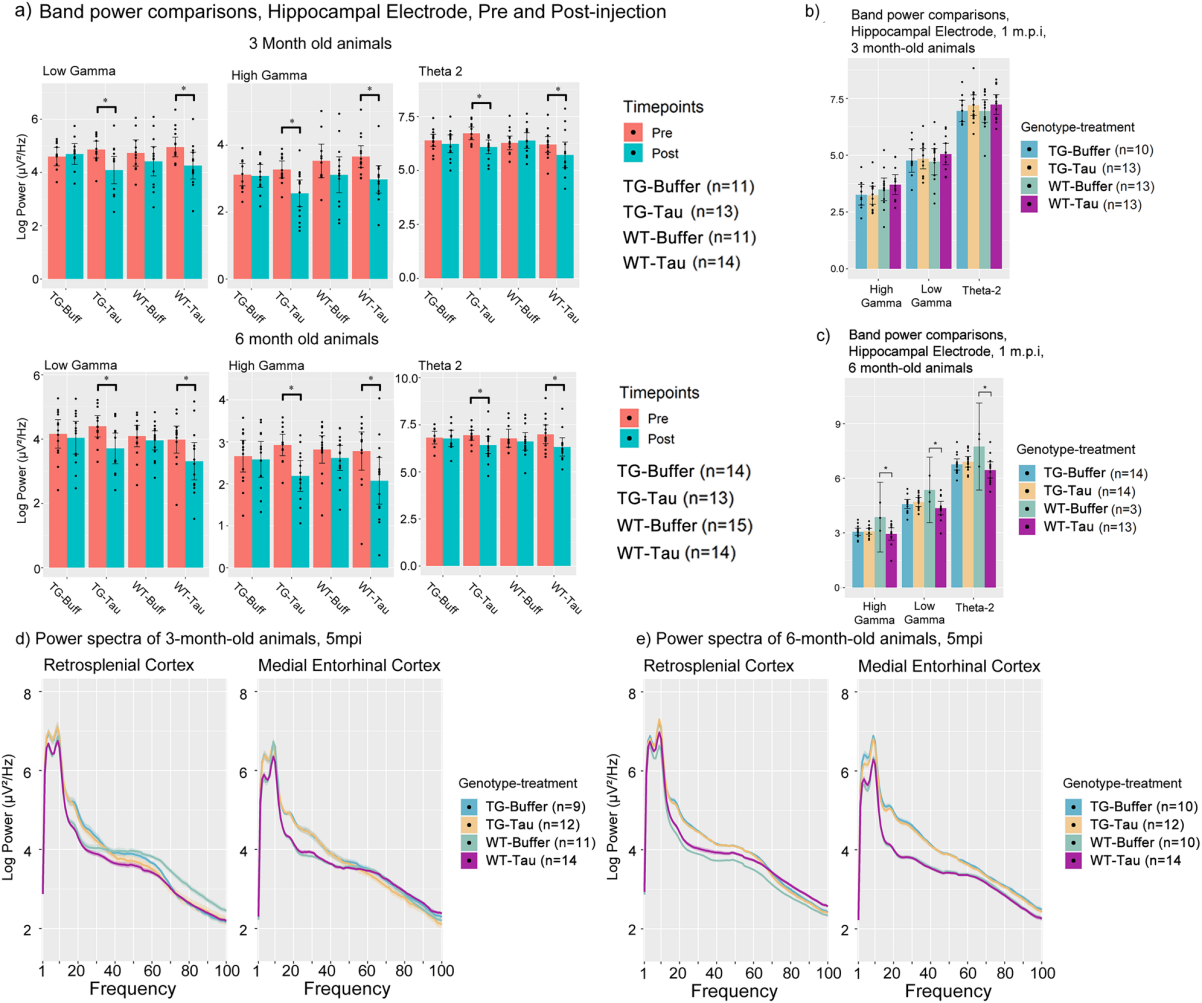
A comparison of power values immediately after and before AD-tau injection as well as power values at 1 m.p.i. (**a**) Log power bar plots from 3-and 6-month-old animals showing immediate changes in Theta 2, Low and High Gamma power at Pre-injection (Pre) and Post-injection (Post). (**b**) Bar plot comparisons of 3-month-old animals for each of the bands investigated for pre- and post-injection changes in power spectra at 1 m.p.i. (**c**) Bar plot comparisons of 6-month-old animals for each of the bands investigated for pre- and post-injection changes in power spectra at 1 m.p.i. (Due to the impact of Covid-19 lockdowns, several datapoints from the 6-month-old 1 m.p.i. timepoint for WT- Buffer group could not be obtained, thus resulting in *n* = 3). Log Power spectra from 1 to 100 Hz of the retrosplenial cortex of (**d**) 3-month-old animals and (**e**) 6-month-old animals at 5 months post injection. (m.p.i. refers to months post injection). All bar plots are Mean + SEM. Asterisks indicate significant comparisons (*p* < 0.05).

Immediate decreases in both theta-2, low and high gamma power 1 day after tau seed injection were observed in the hippocampal electrode of animals injected with AD-tau but not in buffer-injected animals of both age groups (Fig. 5a, Table 3a). This was also observed in both TG and wild-type animals, suggesting that the effects are not differentially affected by genotype or age. Because this reduction was also noted to only be present in the hippocampal electrode, local to the injection site (Supp. Fig. 3a, c), and was not noted in buffer-injected animals, this finding suggests it to be a direct effect of the AD-tau injection. This relationship was also investigated for the other frequency bands, but no significant differences were noted (Supp. Fig. 3b), suggesting a specific interaction with theta-2, gamma oscillations and AD-tau injections.

**Table 3:** Table containing the pairwise comparisons of power spectra values immediately before and after tau seeding, as well as longitudinal pairwise power spectra comparisons, and pairwise comparisons of Higuchi fractal dimension scores at different timepoints.

Frequency band	Pairwise comparison	Estimate	SE	df	T ratio	*P* value
**(a) Pre and post injection power spectra comparisons in the Hippocampus**						
Theta 2	3mo Tg Tau Post—3mo Tg Tau Pre	− 0.5623	0.1628	112	− 3.4548	0.0011
	6mo Tg Tau Post—6mo Tg Tau Pre	− 0.5703	0.1680	112	− 3.3953	0.0014
	3mo Wt Tau Post—3mo Wt Tau Pre	− 0.6912	0.1653	112	− 4.1829	0.0001
	6mo Wt Tau Post—6mo Wt Tau Pre	− 0.4815	0.1615	112	− 2.9815	0.0052
Low gamma	3mo Tg Tau Post—3mo Tg Tau Pre	− 0.8025	0.1518	112	− 5.2869	0.0001
	6mo Tg Tau Post—6mo Tg Tau Pre	− 0.6461	0.1567	112	− 4.1240	0.0001
	3mo Wt Tau Post—3mo Wt Tau Pre	− 0.7126	0.1541	112	− 4.6240	0.0001
	6mo Wt Tau Post—6mo Wt Tau Pre	− 0.6713	0.1506	112	− 4.4568	0.0001
	3mo Tg Buffer 1mpi—3mo Tg Tau 1mpi	− 0.0152	0.1407	112	− 0.1082	0.9313
	3mo Wt Buffer 1mpi—3mo Wt Tau 1mpi	− 0.2753	0.1318	112	− 2.0888	0.0564
	6mo Tg Buffer 1mpi—6mo Tg Tau 1mpi	− 0.0961	0.1336	112	− 0.7190	0.5349
	6mo Wt Buffer 1mpi—6mo Wt Tau 1mpi	0.8917	0.2166	112	4.1168	0.0002
High Gamma	3mo Tg Tau Post—3mo Tg Tau Pre	− 0.7348	0.1346	112	− 5.4573	0.0001
	6mo Tg Tau Post—6mo Tg Tau Pre	− 0.6687	0.1389	112	− 4.8128	0.0001
	3mo Wt Tau Post—3mo Wt Tau Pre	− 0.6818	0.1367	112	− 4.9875	0.0001
	6mo Wt Tau Post—6mo Wt Tau Pre	− 0.7082	0.1336	112	− 5.3013	0.0001
	3mo Tg Buffer 1mpi—3mo Tg Tau 1mpi	0.0611	0.1325	112	0.4612	0.7169
	3mo Wt Buffer 1mpi—3mo Wt Tau 1mpi	− 0.1965	0.1242	112	− 1.5829	0.1715
	6mo Tg Buffer 1mpi—6mo Tg Tau 1mpi	0.0004	0.1263	112	0.0034	0.9979
	6mo Wt Buffer 1mpi—6mo Wt Tau 1mpi	0.8205	0.1966	112	4.1737	0.0002

High Gamma by region	Pairwise comparison	Estimate	SE	df	T ratio	*P* value
**(b) Longitudinal Power changes**						
Hippocampus	3mo Tg Buffer 1mpi—3mo Wt Buffer 1mpi	− 0.2015	0.1325	112	− 1.5205	0.1904
	3mo Tg Tau 1mpi—3mo Wt Tau 1mpi	− 0.4591	0.1244	112	− 3.6913	0.0009
	3mo Tg Buffer 3mpi—3mo Wt Buffer 3mpi	− 0.4311	0.1559	112	− 2.7659	0.0132
	3mo Tg Tau 3mpi—3mpi Wt Tau 3mpi	− 0.5416	0.1483	112	− 3.6525	0.0010
	3mo Tg Buffer 5mpi—3mo Wt Buffer 5mpi	− 0.4993	0.1594	112	− 3.1320	0.0048
	3mo Tg Tau 5mpi—3mo Wt Tau 5mpi	− 0.5213	0.1320	112	− 3.9480	0.0004
	6mo Tg Buffer 1mpi—6mo Wt Buffer 1mpi	− 0.7288	0.1954	112	− 3.7289	0.0008
	6mo Tg Buffer 3mpi—6mo Wt Buffer 3mpi	− 0.3141	0.1242	112	− 2.5300	0.0239
	6mo Tg Buffer 5mpi—6mo Wt Buffer 5mpi	− 0.4374	0.1253	112	− 3.4919	0.0016
Retrosplenial cortex	3mo Wt Buffer 5mo—3mo Wt Tau 5mo	0.4061	0.1338	112	3.0352	0.0063
**Low Gamma by region**						
Retrosplenial cortex	3mo Wt Buffer 5mpi—3mo Wt Tau 5mpi	0.3258	0.1433	112	2.2737	0.0373

**(c) Hippocampal fractal dimension scores at 5 mpi**						
HFD values	6mo Tg Buffer 3mpi—6mo Tg Tau 3mpi	0.0313	0.0139	112	2.2563	0.0409
	6mo Tg Buffer 5mpi—6mo Tg Tau 5mpi	0.0364	0.0139	112	2.6142	0.0175
	6mo Wt Buffer 5mpi—6mo Wt Tau 5mpi	− 0.0360	0.0137	112	− 2.6322	0.0167

(a) Power spectra band values before and after injection within the same genotype-treatment group, (b) Power spectra changes between genotype-treatment groups across time post injection, and (c) Hippocampal Higuchi Fractal Dimension scores at 3- and 5-months post injection. (m.p.i. refers to months post injection). Pre refers to Pre-injection timepoint. Post refers to 1 day after injection. MEC refers to Medial Entorhinal Cortex, HPC refers to Hippocampal CA1, Tha refers to thalamus, RSC refers to Retrosplenial Cortex. TG refers to APP.PS1 animals, WT refers to wild-type. Tau refers to tau-seeded mice, Buffer refers to phosphate-buffered saline injected mice. SE refers to standard error. Df refers to degrees of freedom. Estimate refers to the estimated difference in value between pairwise comparisons. HFD refers to Higuchi fractal dimension. mo refers to months-old at injection.

However, at 1 month post injection, power spectra levels of theta-2, low and high gamma power were not significantly different across all treatment-genotype groups, indicating a reversion or disappearance of this particular change with the exception of one genotype-treatment group (Fig. 5b, c, Table 3a). Notably, only 6-month-old WT-tau animals exhibited a decrease in low and high gamma power compared to buffer-injected animals at 1 month-post injection (Fig. 5c, Table 3a), suggesting that age and genotype may be factors influencing this effect. These results indicate that the immediate consequences of seeding impair theta 2 and gamma oscillatory band power but are transient.

### Longitudinal neurophysiological changes associated with genotype and seeding

We attempted to understand if any differences were present in the power spectra at 5 months post injection, which show the most abundant amount of pathology, in regions exhibiting more AT8-positive tau pathology (i.e., RSC and MEC). The lack of a significant interaction effect reported above preluded this, confirming a lack of an overtly significant effect when comparing TG-tau animals and TG-buffer animals (Fig. 5d, Table 3b). Only the RSC electrode showed significant, but marginal decreases in low and high gamma activity, and only in 3-month-old animals but not 6-month-old animals (Fig. 5e, Table 3b).

**Figure 6:**
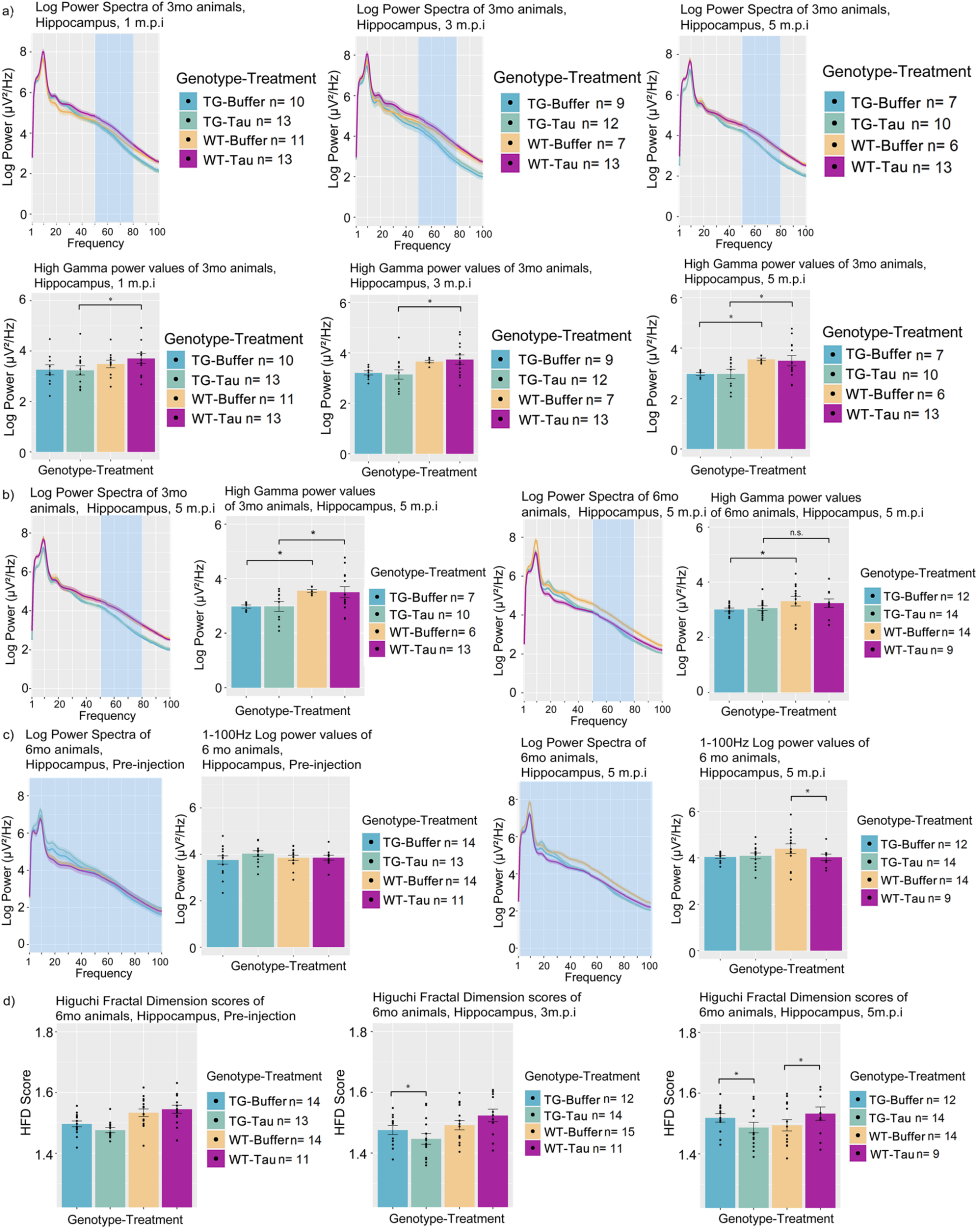
Longitudinal power spectra comparisons and Higuchi fractal dimension score comparisons (**a**) Log power spectra and accompanying bar plots from 3-month-old animals showing longitudinal change in high gamma power across 5 m.p.i. and treatment-genotype groups. Blue regions indicate the region of the power spectra used for the plotting of bar plots. (**b**) Bar plot comparisons of 3-month-old and 6-month-old animals for high gamma power. (**c**) Whole power spectra (1-100 Hz) comparison between 6-month-old animals at 5 m.p.i. and pre-injection. (**d**) Higuchi fractal dimension score bar plots of the hippocampus of 6-month-old animals at pre-injection, 3 and 5 m.p.i. (m.p.i. refers to months post injection). All bar plots are Mean + SEM. Asterisks indicate significant comparisons (*p* < 0.05).

Subsequently, we turned our attention to analysis of the injection site, the hippocampus. We report that 3-month-old TG animals exhibit high gamma power differences in the hippocampus, which appear to be influenced by genotype and treatment. TG-tau animals exhibit significantly reduced high gamma power compared WT-tau animals beginning at 1 m.p.i., up until 5 m.p.i. (Fig. 6a, Table 3b). In contrast, when comparing TG-buffer and WT-buffer animals, this relationship is absent until 5 m.p.i., suggesting that tau seeding may be influencing this relationship. The difference between WT-Buffer and TG-Buffer animals at 5 m.p.i. suggests a potential longitudinal effect, which is already present between WT-Tau and TG-Tau animals at 1 m.p.i. This indicates the difference in high gamma power between TG and WT animals may appear earlier due to tau-seeding.

In 6-month-old animals, when testing for significant differences between TG-tau and WT-tau animals, this pairwise comparison was noted to be non-significant (Fig. 6b, right panels, Table 3b). This significant relationship at 5 m.p.i. was present only between WT-buffer and TG-buffer animals (Fig. 6b, right panels, Table 3b), indicating a differential effect of tau associated with the age at which the animal was injected in WT animals. WT-tau animals at 5 m.p.i. exhibited lower high gamma power compared to WT-buffer controls, and the difference was not significant (i.e., *p* > 0.05).

However, this was not limited to high gamma power, as hippocampal power spectra from the range of 1–100 Hz were noted to be significantly reduced in WT-tau animals compared to WT-buffer animals (Fig. 6c, Table 3b), at 5 m.p.i. Taken together, these results appear to indicate that differences in high gamma power are present between TG and WT animals. This appears to be influenced by tau-seeding, whereby WT-Tau animals appear to exhibit this difference earlier than buffer injected animals in animals injected at 3 months of age. In animals injected at 6-months of age, this relationship was not significantly present in WT-tau animals, suggesting a differential effect at which age animals were injected.

In terms of HFD scores, 6-month-old TG-tau animals showed significant decreases in the hippocampus at 3 m.p.i. and 5 m.p.i. compared to TG-Buffer animals (Fig. 6d, Table 3c). This relationship was noted to be absent prior to injections, suggesting it to be a consequence of tau injections. Significant differences between WT-tau and WT-buffer animals also began to emerge at 5 m.p.i. (Fig. 6d, Table 3c) but paradoxically show increases in WT-tau as opposed to reductions in TG-tau animals. Again, other brain regions investigated did not exhibit significant effects associated with tau seeding at the different time points. This indicates that the effects of tau seeding appear to significantly reduce HFD scores in TG-tau animals, but in contrast, WT-Buffer animals show reduced HFD scores compared to WT-Tau animals, suggesting a differential effect associated with genotype, indicating that the presence of amyloid plaque pathology may be affecting this relationship. No significant differences in HFD scores were noted in animals injected at 3-month of age.

## Discussion

In both clinical and preclinical research, understanding the dysfunction associated with pathology in AD is of great importance for understanding the neurophysiological trajectory of the disease. Based on this, therapeutic approaches can be evaluated on the basis of modifying this trajectory. Our study has examined a longitudinal progression of both amyloid and AT8-positive tau pathology in an animal model simultaneously exhibiting both pathologies characteristic of AD, at different ages, and across a 5-month post injection time window. By complementing this approach with the electrophysiological readouts, we sought to understand and characterize the neurophysiological trajectory associated with the development of pathology.

We have shown that the development and progression of AT8-positive tau pathology in this study occurs longitudinally in animals injected with AD tau seeds but exhibit more pathology in APP.PS1 animals compared to wild-type animals. The longitudinal development of AT8-positive tau seen in our study and from previous reports provides additional support for the ability of pathogenic species of tau to be capable of spreading across the brain in a prion-like manner^31^. It is believed that tau can spread transsynaptically^32^, as well as locally from nearby neurons^33,34^, although it is not clear which form of spreading is dominant in our study, and a topic for subsequent investigation.

This study builds upon prior work from Guo and colleagues^17,19^, supporting the hypothesis that the progression of this form of tau pathology is facilitated by the presence of amyloid plaque pathology. Notably, we have demonstrated that the presence of amyloid pathology increases the development of AT8-positive tau pathology, from histological images, the significant interaction effects of AT8-positive tau pathology, colocalized pathology, as well as correlation between pathology. In addition, the development of AT8-positive tau pathology in APP.PS1 mice appear to favor isocortical and entorhinal areas instead of the hippocampus. This may be due to the fact that the hippocampal CA1 region generally features comparatively less plaque pathology than cortical regions at younger ages in transgenic mouse models with amyloid pathology^35^, and could be the main factor limiting the development of AT8 pathology in the hippocampus based on our data.

Previous reports characterizing tau pathology induced by this seeding method by He and colleagues have indicated three primary forms of tau pathology: Neuritic plaque tau, which is plaque-associated tau pathology, and non-plaque associated tau pathology which include: intracellular neurofibrillary tangles, and neuropil threads^19^. While the scope of this study did not include a detailed characterization of which forms are present, at least 2 forms of tau pathology are likely present in our study, as demonstrated by the presence of plaque-associated tau seen in TG-tau animals, and non-plaque-associated tau seen in WT-tau animals.

In relation to the neurophysiology of these animals, we have shown that comparing the power spectra of TG animals to WT animals, the MEC, and RSC exhibited significant increases in power prior to injections and could be indicative of network hyperexcitability^12,13,36^. Network hyperexcitability has been suggested to be a prodromal indicator of AD, and reflects a state of increased neuronal network activity in the early amnestic mild cognitive impairment stage of the disease^36,37^. Notably, AD patients have been reported to exhibit increased network activity in the hippocampus^38–40^, medial temporal lobe^41^ as well as the default mode network^42^ as possible indicators of network hyperexcitability, which may correspond to increases in portions of the power spectra. However, based on our results, cortical regions rather than the hippocampal region feature increased power spectra in APP.PS1 mice, suggesting that in this animal model, hyperexcitability may be present more overtly in cortical regions.

Gamma band power has been associated with the function and integrity of inhibitory interneurons^43^, which has been suggested to be compromised in AD patients^44,45^ and animal models of AD^44,46–48^. The dysfunction of said interneurons has also been related to the propensity for the development of seizure-like phenomena, which is present in several animal models featuring amyloid pathology^48,49^. Additionally, the increase in power of lower frequency (i.e. Delta and Theta) oscillations, while also accompanying decreases in higher frequency gamma (50–80 Hz) in APP.PS1 animals may be indicative of a “slowing” of the EEG^21^, a phenomenon commonly reported to occur in AD^50^.

Another factor driving the development of AT8 pathology near amyloid plaque pathology may stem from the neurophysiological changes proximal to plaque pathology. This is also likely reflected in the increased power spectra in the medial entorhinal area and isocortex, which likely contain more amyloid pathology, and previous reports examining increased neuronal activity near plaque pathology^12^. Tau exosomal release has been suggested to be correlated with neuronal activity^34,51,52^ and could also be another explanation for increased AT8-positive tau pathology in APP.PS1 animals, particularly near amyloid pathology in our study. Thus, the increased neuronal activity near plaques may be driving increased tau pathology, and a subject for further investigation (e.g., modulation of network activity via pharmacological compounds).

Another notable neurophysiological consequence associated with the injection of tau seeds was the immediate impairment of theta and gamma power in the region of injection, suggesting a silencing or reduction of neuronal activity associated with these AD-tau seeds. The identity of these AD-tau seeds have been previously reported to be purified tau, accompanied by minute amounts of Aβ42, Aβ40 and α-syn^17,19^. To our knowledge, the immediate and longitudinal effects of this form of tau induction have not been previously characterized in terms of neurophysiological consequences, but previous reports regarding transgenic approaches to studying tau pathology^8,14,15^ have indicated a reduction in neuronal activity associated with tau-associated experimental manipulations. The findings of injections appear to fall in line with the currently understood role of tau pathology.

However, the absence of this effect at 1 month-post-injection suggests a short-term effect associated with tau seeding, rather than due to the pathology itself. In addition, animals exhibiting clear histological signs of AT8-positive tau pathology in the RSC and MEC also failed to demonstrate significant neurophysiological differences even at the oldest ages and timepoints. This could potentially reflect the difficulty in detecting EEG-associated abnormalities, especially at the earlier and middle stages of the disease^36^. Alternatively, other methods not tested in the scope of this study may be able to detect more subtle changes associated with incipient pathology.

We note however, a reduction of hippocampal power in older WT mice injected with tau, indicative of an overall reduction of network activity, which was not present in comparable APP.PS1 animals. This relationship is also differentially influenced by both the genotype and age of the animal, suggesting that older animals are more susceptible to pathological tau insults, but may be counteracted or obscured by the presence of the APP.PS1 genotype.

Lastly, alterations in HFD scores were noted to be able to identify significant changes associated with AD-associated pathology in our study. HFD scores have been reported to be reduced in AD patients as the disease progresses^23,53^. This in line with our data showing reduced HFD scores in APP.PS1 animals compared to WT animals at pre-injection baselines as well APP.PS1 animals injected with tau. Interestingly, the effects of tau seeding on HFD scores were divergent depending on the genotype of the animal, with WT animals exhibiting increases and APP.PS1 animals exhibiting decreases. This suggests a role implicating amyloid pathology for the observed differential outcomes and may indicate a synergistic effect of amyloid and tau pathology on HFD scores. This highlights a potentially useful role of HFD for the discrimination between different pathological states associated with tau, amyloid or both, but further understanding of the mechanistic processes driving HFD changes is needed.

However, the disconnect between the clear pathological outcomes and the lack of neurophysiological effects is puzzling, particularly in APP.PS1 animals. The hypothesized interaction between amyloid and tau pathology was anticipated to produce a more severe neurophysiological phenotype in contrast to either pathology, but a general lack of an effect was present, particularly in regions that contained more pathology (i.e., cortical regions).

Some reports have suggested that animal models containing amyloid-associated transgenes may exhibit altered neurophysiological phenotypes at the slice and cellular level^54,55^, and related to intracellular levels of APP/amyloid-beta, rather than extracellular plaques. Investigation of cellular or slice neurophysiology following longitudinal seeding experiments could reveal changes at the cellular level associated with tau seeding which may not be readily detectable using aggregated neuronal activity in the LFP.

Another possibility may be that the effect of amyloid pathology may be dominating that of tau seeding, given the relative proportions of the pathologies, as well as the suggested opposing effects of amyloid and tau in terms of excitability^14^, neurophysiological effects may be slightly more pronounced in wild-type animals lacking amyloid. Longer-term characterization of neurophysiological effects in APP.PS1 may reveal neurophysiological phenotypes as tau pathology develops even more.

However, there are some limitations associated with the present study that need to be considered when interpreting the findings. While it is clear the injection of tau seeding material elicits effects immediately in contrast to buffer injections, the material is likely comprised of tau, minimal levels of amyloid-beta and alpha-synuclein^17^. Ideally, a control solution derived from the purification of multiple healthy, age-matched subjects (since the seeding material is derived from a pool of patients) would allow for teasing apart the factors that contribute to seeding and neurophysiological changes but is not easily obtainable.

Second, this was an exploratory study in which the effects of multiple independent variables on the outcomes of interest were investigated. Even though the total number of animals that were included in the study was substantial (i.e., *N* = 72 and *N* = 64 in the histology and electrophysiology cohorts per age group), the statistical power can be adversely affected when many independent variables (and their interactions) are considered. It can thus not be excluded that some non-significant results are attributable to a lack of power (or put alternatively, to type II errors / false negatives), especially for independent variables that have only a small effect size on the outcomes of interest. Confirmatory experiments of specific main effects and/or interaction terms of specific scientific interest can be useful to corroborate the results of the present study. On a related note, given the exploratory nature of the study it was not possible to conduct a proper power analysis/sample size computation, because doing so requires reasonable estimates of the effect sizes of the different fixed effects and variability components (i.e., random effects). This information was not available at the time that the study was set-up.

Another potential limitation associated with transgenic animal models, may be the effects of transgene insertion or protein overexpression influencing some of the endpoints of the study. This has recently been highlighted as a concern^56,57^. Further research can be done in a separate, but similar amyloid animal model (e.g., APP knock-in animals) to confirm if the effects associated with pathologies are consistent or differ according to the strain of the animal and teasing apart these potential confounds.

Lastly, while PFTAA has been reported to detect the presence of tau pathology as well as amyloid pathology^58^, the conditions at which PFTAA are used in this study are unlikely to confound the quantification of amyloid and tau pathology. This is evident in the lack of PFTAA signal in WT-tau mice, which do not exhibit amyloid pathology, but still exhibit tau pathology following seeding.

Our study has attempted to provide a comprehensive exploratory longitudinal characterization and evaluation of the pathological changes associated with the interaction between amyloid and tau pathology, as well as neurophysiological consequences in the context of spectra changes, phase-amplitude coupling and fractal dimension scores. The lack of obvious neurophysiological changes in the face of overt, visible and quantifiable pathology serves to highlight the complexity associated with identifying effective indicators of incipient pathology. This is also a problem being faced in clinical settings that aim to determine suitable neurophysiological biomarkers for the identification of patient populations at risk of progressing to a confirmed diagnosis of AD. The application of nonlinear methods such as fractal dimension scores appear sensitive in detecting more subtle changes associated with both amyloid and tau pathology, whereas power spectra changes appear to identify amyloid-associated changes and initial tau seeding-associated changes, but not overt AT8-positive tau pathology changes. Nonetheless, this animal model may serve useful as a screening platform for the identification of robust biomarkers associated with pathology, which may eventually be translated to the clinic.

## Methods

### Animal Cohorts and usage

Data were obtained from heterozygous APP KM670/671NL.PS1/L166P (B6.Cg-Tg(Thy1-APPSwe,Thy1-PSEN1*L166P)21Jckr (line 21)) mice and Wild-type C57BL6 littermates. Transgene expression was noted to be stable, with the current backcrossing at 21 generations. These mice have been reported to exhibit threefold APP overexpression^25^. Animal housing conditions, diet, light–dark cycles, except for food restriction were identical to the conditions listed in^59^. Animals were given ad libitum access to food and water. Animals were genotyped using PCR of ear punches.

For the purposes of characterizing and understanding the neuropathological and neurophysiological effects of tau seeding in APP.PS1 mice, we established 2 cohorts of animals (Fig. 1a): Histology (Fig. 1b) and Electrophysiology (Fig. 1c) cohorts. Each cohort consisted of 2 age groups (3-month-old and 6-month-old, *n* = 64 for electrophysiology and 72 for histology per age group), which were further comprised of 4 genotype-treatment groups (*n* = 16 for electrophysiology and *n* = 18 for histology): APP.PS1 + tau seeding (TG-tau), C57BL6 wild-type controls with tau seeding (WT-tau), and respective buffer-injected controls (TG-Buffer and WT-Buffer). For the histology cohort, animals were sacrificed at 3 timepoints (1, 3 or 5 months) after injection (Fig. 1a, *n* = 6 per age group per genotype-treatment group per timepoint). For the electrophysiology cohort, animals were recorded at the same timepoints as the histology readouts (1, 3 and 5 months after injection), and sacrificed at the end of the experiment. All cohorts and groups were equally balanced for sex. Animals were randomly assigned to each group using random number generation. Animals were also recorded in a randomised manner using the same random number generator approach. Due to the exploratory pilot nature of this study, a reference study was not available for power analysis for a proper sample size estimation. The treatment conditions and genotypes of the animals were blinded to the experimenter until the point of data analysis. This study is reported in accordance with the ARRIVE guidelines.

All in vivo and in vitro studies were performed in strict accordance with the guidelines of the Association for Assessment and Accreditation of Laboratory Animal Care International (AAALAC) and with the European Council Directive of 24 November 1986 (86/609/EEC) and European Ethics Committee directive (2010/63/EU) for the protection of laboratory animals. In line with Belgian governmental directives all protocols were approved by the Animal Care and Use Committee of Janssen Pharmaceutica NV.

### In vitro and ex vivo methods

#### AD-tau seed purification

Human brain samples were obtained from the University of Washington brain bank as a generous gift from Virginia M.Y. Lee and John Q. Trojanowski. The use of post-mortem brain tissues for research was approved by the University of Pennsylvania’s Institutional Review Board with informed consent from patients or their families. Purification methods for the preparation of AD-Tau seeds were followed according to the protocol listed in^17^ and detailed below. All methods were performed in accordance with the relevant guidelines and regulations.

Patient brain samples stored at -80 °C were thawed and transferred to a precooled metal cooling block. Grey matter was separated using metal forceps and was kept for subsequent extraction of seeding material. Total grey matter weight was recorded and extraction buffer was added before homogenization (QuickPrep, FastPrep-24, 4 m/s, 30 s) and stored at -80 °C.

Homogenates were thawed and centrifuged at 10,000 g for 10 min at 4 °C. The supernatant was separated and kept. The pellet was re-homogenized by adding 30 ml of extraction buffer per 180 ml of homogenate and re-homogenized (QuickPrep, FastPrep-24, 4 m/s, 30 s). This was subsequently centrifuged again at 10,000 g. 25% Sarkosyl was added to the supernatant from the first centrifugation step to a final concentration of 1% Sarkosyl in the mixture and stirred at 100 rpm, room temperature. The supernatant from the second centrifugation step was separated from the pellet and 25% Sarkosyl was added to a final concentration of 1% Sarkosyl. The two volumes of supernatants containing Sarkosyl were combined and stirred for 1.5 h at 100 rpm, room temperature and subsequently referred to as the Sarkosyl mixture.

The Sarkosyl mixture was subsequently centrifuged at 150,000 g for 75 min at 4 °C. The resulting supernatant was decanted, and the pellet gently rinsed with PBS 3 times. Pellets were reconstituted with PBS and vortexed at 2000 rpm for 1 min. Another 23 ml of PBS was added and another round of centrifugation at 250,000 g, for 30 min, at 4 °C was performed. The supernatant was decanted, and the pellet transferred to a 2 ml Eppendorf tube, which was subsequently filled with PBS and rocked for 12–16 h (Hoolamixer, orbital 5 rpm 15 s, reciprocal 45° 10 s, vibro 5° 5 s).

This was subsequently centrifuged at 1000 g for 1 min at room temperature before using a 20-, 23- and 26-gauge needle and syringe consecutively to break the pellet into smaller pieces by passing the suspension through the syringes repeatedly. This mixture was sonicated (Sonotrode, Amplitude 100%, Cycle = 50% and Total watts of 200 W). This was subsequently centrifuged in 1 ml ultracentrifuge tubes at 100,000 g for 30 min at 4 °C. The supernatant was removed and reconstituted with PBS, before repeating the same process of breaking the pellet using a 23 and 26-gauge needle. This was subsequently centrifuged at 1000 g for 1 min at room temperature and sonicated again at the same settings.

A final centrifugation at 10,000 g was carried out for 30 min at 4 °C and the supernatant collected as AD-Tau seeding material, subsequently stored at -80 degrees Celsius. This was performed on brain samples from 6 different patients on different days and the purified seeds from each sample were pooled. Purified seeds from each purification, as well as the pooled sample were checked for seeding in mouse primary neurons. For more details regarding the properties of the seeding material (purity, protein concentration, etc.), as well as patient data of brain samples, please refer to Supplementary Table 5.

#### Brain clearing method

Cleared brain samples were bisected along the midline and the hemisphere containing the electrode implantation sites and/or injection were placed into 15 ml flip-cap tubes. Fluorescent labelling and clearing of brain hemispheres were done based on the iDISCO + protocol for all brains^60^. Hyperphosphorylated tau was specifically detected using an AT8 antibody (pSer202/Thr205/PSer208, produced at Janssen Pharmaceutica) conjugated with a near-infrared fluorescent tag (PerkinElmer VivoTag 680XL) following the manufacturer’s protocol prior to labelling (9.18 µg/ml in 1.8 ml for 14 days per hemisphere). Pentameric formyl thiophene acetic acid (PFTAA) was used for selective staining of protein aggregates (30 µM in 1.8 ml for 1 day per hemisphere)^61^. Samples were stored in 5 ml tubes containing dibenzyl ether until imaging was carried out. An overview of the workflow can be seen in Fig. 1b.

#### Light sheet microscopy

Cleared samples were imaged using a light sheet microscope (Ultramicroscope II, Lavision Biotec GmbH), equipped with an Olympus MVPLAPO 2X (NA 0.50) objective lens and a DBE-corrected LV OM DCC20 dipping cap. Images were acquired with a Neo sCMOS camera (Andor) at a total magnification of 1.6X. Z-step were set at 10 µm, giving a voxel size of 4 µm^2^ × 10 µm. A linear blending algorithm was used to merge on the fly both left and right light sheets. Sagittal pictures were framed by 2-tiled mosaic was done using 488 nm, 561 nm and 640 nm emission lasers with 525/50 nm, 620/60 nm and 680/30 nm emission filter respectively. The exposure time was defined and fixed at 100 ms and laser power was kept constant across batches.

#### Image analysis

The image semiquantitative analysis protocol used for the detection of both PFTAA and AT8 signals was adapted from ClearMap^62^ and further refined from Detrez et al.^63^. Subsequently, the total number of AT8-positive or PFTAA-positive voxels for a given brain region volume was calculated and expressed as a pathological load (% of voxels stained/total number of voxels in that region). Additionally, we dilated the binary segmented plaques by 5 pixels for defining boundaries of plaque-associated AT8-positive tau versus non-associated AT8-positive tau. AT8-positive tau within this boundary were considered as plaque-associated tau. For every experiment, bar graphs expressing the pathological load (y-axis) were constructed across time (x-axis) with standard error of mean error bars. Pathology was evaluated in several brain regions of interest as delineated by mapping to the Mouse Allen Brain Atlas using Elastix^64,65^, namely: the hippocampal region, entorhinal area, isocortex and thalamus. For all graphs, the LabelRatio refers to the number of voxels stained divided by the total number of voxels in that entire brain region.

#### In Vivo methods

##### Surgical procedures

Surgeries were carried out when mice were 2–2.5 months old and 5–5.5 months old. Anesthesia was induced via isoflurane inhalation (O2, N2O and 5% isoflurane), followed by the shaving of the fur and disinfection with Isobetadine (Meda Pharma SA, Belgium) and 70% Ethanol. Analgesics (dipidolor, 0.025 mg/kg, Xylocaine, 10%), and eye ointment (Opticorn A, EcuPhar BV, Belgium) were applied to the animal prior to insertion into a stereotactic frame. The animal was maintained under isoflurane during surgery (O2, N2O and 2–2.5% isoflurane), and kept at 37–38 °C using a heating pad. An incision was made on the skin along the sagittal plane to expose the skull, and sutures to hold the skin apart at the lateral edges of the opening. The tilt, yaw and roll of the head was adjusted for by measuring the DV differential between the bregma and lambda sutures. All differentials were corrected to within 0.05 mm prior to drilling. Drilling locations were measured relative to bregma and drilled by hand. Stainless steel screws were affixed over the left frontal and right occipital lobes to secure the implant.

For the histology cohort, the dura was punctured by a hypodermic needle (Precisionglide, 25 g, BD) followed by cannula implantation (AP: -2.2, ML:1.8, DV:1.7), (C315IA/SPC, 26G, Plastics One Inc), fixed using dental cement (Relyx Unicem 2 cement, 3 M United States) and cured with dental light.

For the electrophysiology cohort, single polyamide-coated stainless-steel wire electrodes (100 μm diameter with a blunt-tip, Peira bvba, Belgium) were implanted. The dura was punctured by a hypodermic needle (PrecisionGlide, 25 g, BD) at each location and implantation of electrodes were carried out with these coordinates: Medial entorhinal cortex (AP: -4.8, ML:3.25, DV:2.2), Hippocampal CA1 (AP: -2.2, ML:1.8, DV:-1.4), Retrosplenial cortex (AP: -1.75, ML: 0.5, DV: 1.0), Thalamus (AP: -0.83, ML:0.75, DV:2.75), and Reference (AP: -1.0, ML:1.2, DV:0.8), followed by the cannula (AP: -2.5, ML:2.45, DV:1.4, lateral angle of + 24.5 degrees ,C315GAS-5/SPC, Plastics One). Each electrode was fixed with dental cement (Relyx Unicem 2 cement, 3 M United States) and cured using a dental light. The ground screw electrode was implanted in the skull approximately 1 mm posterior to lambda. Subsequently, a multichannel connector (Nano strip connector, Omnetics, Minneapolis, USA) was connected to the electrodes and affixed using dental cement.

The skin surrounding the surgical site was sealed using veterinary glue (Vetbond, 3M United States) after either surgical procedure. Mice recovery was closely monitored until they were fully recovered (approximately ten to fourteen days).

##### Animal injections

Animals were injected with either AD-tau seeds or sterile phosphate-buffered saline (PBS) buffer solution via the cannula implanted in the hippocampus at 3 or 6 months of age. Animals were anesthetized and mounted into the stereotactic frame as in the surgical procedure. The needle used for injections was a Hamilton 10 μl syringe mounted onto an injection robot (StereoDrive, Neurostar, Germany). The injection needle was fitted with a custom tube and locking needle adaptor for the cannula (C315IAS-5/SPCm, Plastics One Inc). Injection speed was set as 0.2 μl per minute and a volume of 5 μl was injected, with a waiting time of 5 min after injection. Needles were tested for blockage by ejection of 0.1 μl of injectate before and after injection and changed once a blockage was noted. Animals were returned to home cage and monitored for 2 h after injection.

##### Animal euthanasia

Animals implanted with electrodes were electro-lesioned using a stimulator (STG4002-1.6 mA, MultiChannel Systems, GmbH) while under isoflurane anaesthesia (O2, N2O and 2–2.5% isoflurane). A detailed description of the electro-lesion settings can be found in Supplementary Methods M1. Animals were subsequently administered a dose of pentobarbital diluted in saline (120 mg/kg) and perfused with PBS mixed with heparin (10U/mL), followed by 4% paraformaldehyde (PFA). Brains were kept overnight in 4% PFA and washed two times in PBS for 15 min each before transferring to a solution of 0.1% sodium azide and stored at 4 °C.

##### Electrophysiological recording procedures

Mice were recorded in customized plexiglass chambers (modified from Med Associates Inc. Fairfax, Vermont). These Perspex boxes were placed in opaque sound attenuated chambers fitted with a small ventilation fan and a house light. The entire home cage without the cover or food tray of the animal was placed above a plastic pedestal into the box. The house light was switched on during the entire recording duration. Each recording box was controlled by K-limbic software, (Med Associates Inc., version 1.20.2). A video camera (uEye CP, IDS imaging GmbH) was mounted on the top of the chamber to record animal behavior.

Electrophysiological signals were acquired using a 4-channel wireless headstage (W2100-HS4, MultiChannel Systems GmbH) and interface board (W2100-IFB system, MultiChannel Systems GmbH) at a sampling rate of 1000 Hz. The wireless headstages were powered by a 30mAh battery (Wireless-B-30mAh, MultiChannel Systems GmbH). All signals recorded were referenced and grounded to the respective physical electrodes as described in the surgical procedures. Signals and battery levels (> 80%) were checked prior to recording. The MultiChannel Experimenter software (MultiChannel Systems GmbH, version 2.14.0.19346) was used to acquire the recordings and synchronize the video acquisition, which was carried out on a separate computer running the MultiChannel VideoControl software (MultiChannel Systems GmbH, version 2.2.0) at 25 Hz. The duration of each recording session was 1 h in length and carried out 2 h after the start of the dark phase. Animals were returned to the home rack after each session. Animals were recorded on 3 consecutive days at the same time each day to control for circadian effects. Animals were recorded at 5 timepoints after surgical implantation: Pre-injection (4–6 h before injection), post-injection (1 day after injection), 1 month-post-injection (30 days), 3 months-post-injection (90 days), and 5 months-post-injection (150 days).

##### Animal activity level estimation

In order to assess the activity state of the animal for subsequent analysis, the video files of each recording were processed to extract movement and activity information of the animal using DeepLabCut^66^. Once the model snapshot was finalized, the model was incorporated into a custom in-house software based on LabView (National Instruments, USA) for estimating the position of the animal and determining the activity level of the animal. A detailed protocol of the video pre-processing, tracking model, analysis, and activity level estimation can be found in Supplementary Methods M2.

The entire duration of each recording session was divided into 4 s epochs and classified as active or inactive based on whether the activity data crossed the threshold for a sufficient duration during each epoch. The classification of animal activity was a binary state of 0 (inactive) or 1 (active) with an activity threshold calculated from 30% of the peak motion displacement over the entire recording session. Only active epochs were considered for all subsequent EEG analysis.

##### EEG data preprocessing and exclusion criteria

LFP data was acquired from the electrophysiological recordings of the animal and divided into the same time-matched 4 s epochs as used for activity detection, and subsequently processed to remove noise and artefacts using MATLAB 2016a. Animals were also excluded based on noise and artefacts that could not be removed by processing. For a detailed description of the artefact and noise detection processing algorithms, as well as specific exclusion criteria, please refer to Supplementary Methods M3.

##### EEG analysis

Analysis was carried out on the data with 3 primary endpoints: Power spectrum density estimation, phase amplitude coupling and HFD analysis. Frequency bands definitions of delta (1–4 Hz), Theta-1 (4–6), Theta-2 (6–8)^67^, Low Gamma (30–50 Hz) and High Gamma (51–80)^68^ were used for the analysis of each readout. The information extracted from the LFP of each epoch was averaged across all epochs to generate an animal average.

Power spectrum density was estimated using the Welch method^69^ with a Hamming window applied to the signal. The resulting power spectrum was median-filtered across the power spectrum. The resulting power spectrum was subsequently log-normalized using a natural logarithm log_e_^70^ to better meet the assumptions of a normal distribution for subsequent statistical parametric testing. The power density was estimated for each of the frequency bands listed above.

For phase amplitude coupling, signals were convolved using complex Morlet wavelets to generate filtered signals in steps of 5 Hz from 10 to 200 Hz for the amplitude-modulated signals and in steps of 0.5 Hz from 2 to 12 Hz for the phase modulating signals with a Morlet wavelet width of 7 cycles. Phase angles for each filtered signal was calculated using the angle() function in MATLAB. The modulation index (MI) was subsequently calculated based on the approach by Tort et al.^71^ for each phase-amplitude signal pair to construct a matrix of MI values.. The MI values were subsequently averaged across amplitude-frequency band pair ranges. The frequency band pair ranges used for averaging of MI values were: Theta 1-Low Gamma, Theta 1-High Gamma, Theta 2-Low Gamma and Theta 2-High Gamma (as defined above).

Lastly, the calculation of the Higuchi fractal dimension (HFD) (Higuchi, 1988), a nonlinear approach to estimating the fractal complexity of a time-series was carried out using a custom MATLAB script to calculate the HFD value for each 4 s epoch. The equation used for the estimation of the HFD is based on the original equation from Higuchi^72^, reviewed in Kesić and Spasić^73^. The value of the free parameter kmax = 13 was derived based on the approach listed in Spasić et al.^74^.

##### Statistical analysis

Statistical analysis was performed in R v.4.0.5 using RStudio as frontend for development of analysis scripts. A general linear mixed model (GLMM) was fit to the readouts of histology and electrophysiology. A detailed description of the GLMM can be found in Supplementary Methods M4 and M5.

To determine if the interaction effects were significant, a null model containing all other terms except for the interaction term of interest was compared to the model via log-likelihood. Significant interactions were determined if the null model was significantly different from the threshold (alpha = 0.05). Subsequent post-hoc comparisons using Benjamini–Hochberg FDR for multiple comparisons were generated. The threshold for determination of significance was set at q = 0.05 for the multiple comparisons after correction using Benjamini–Hochberg FDR. All data was plotted using ggplot2 as bar graphs and dots representing individual animal values and error bars representing standard errors.

The correlational analysis was performed using the stat_cor method in the ggplot2 library in R using Pearson’s correlation, considering only tau-injected animals, pooling animals from different ages, time-post-injection, sex, brain region and genotype.

## Supplementary Information

Supplementary Information 1.

Supplementary Information 2.

Supplementary Information 3.

Supplementary Information 4.

Supplementary Information 5.
