# Supplementary figures and images for: Neurophysiological effects of human-derived pathological tau conformers in the APPKM670/671NL.PS1/L166P amyloid mouse model of Alzheimer’s disease
Source: Sci Rep. 2022 May 11;12:7784. doi: 10.1038/s41598-022-11582-1 (PMC9094605; doi:10.1038/s41598-022-11582-1)

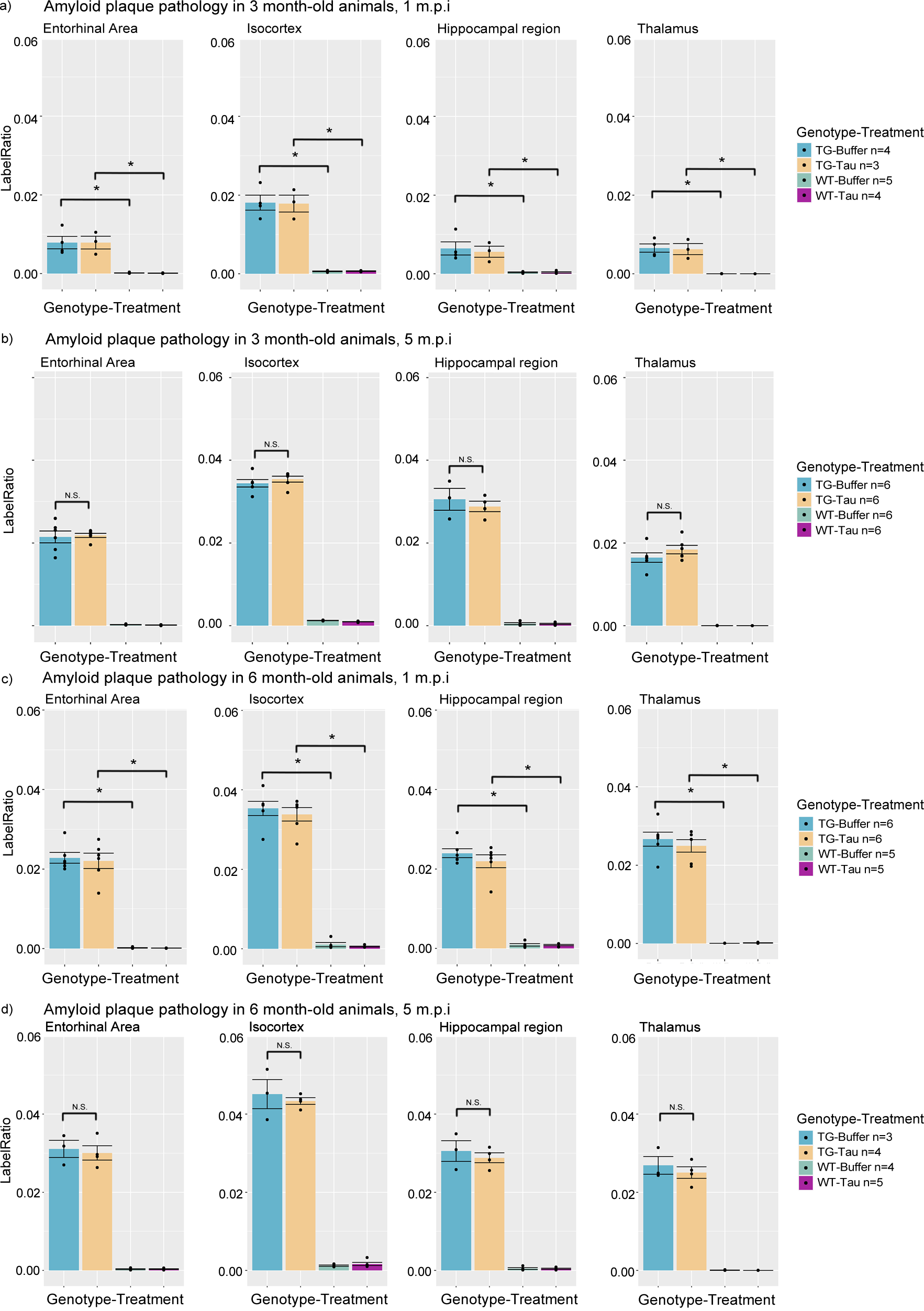

Supplement: Supplementary file 1 — Supplementary Information 1. [file 41598_2022_11582_MOESM1_ESM.png]

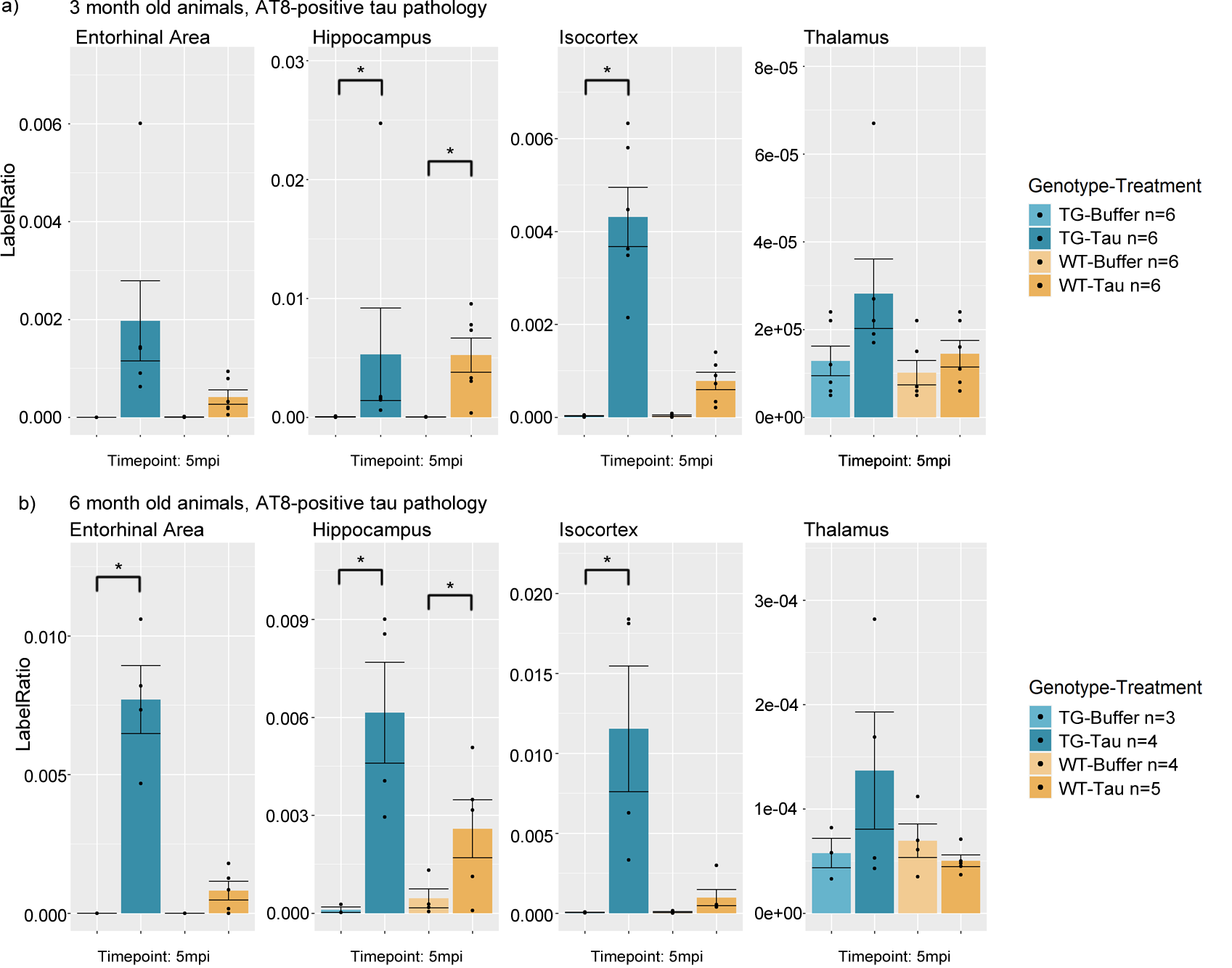

Supplement: Supplementary file 2 — Supplementary Information 2. [file 41598_2022_11582_MOESM2_ESM.png]

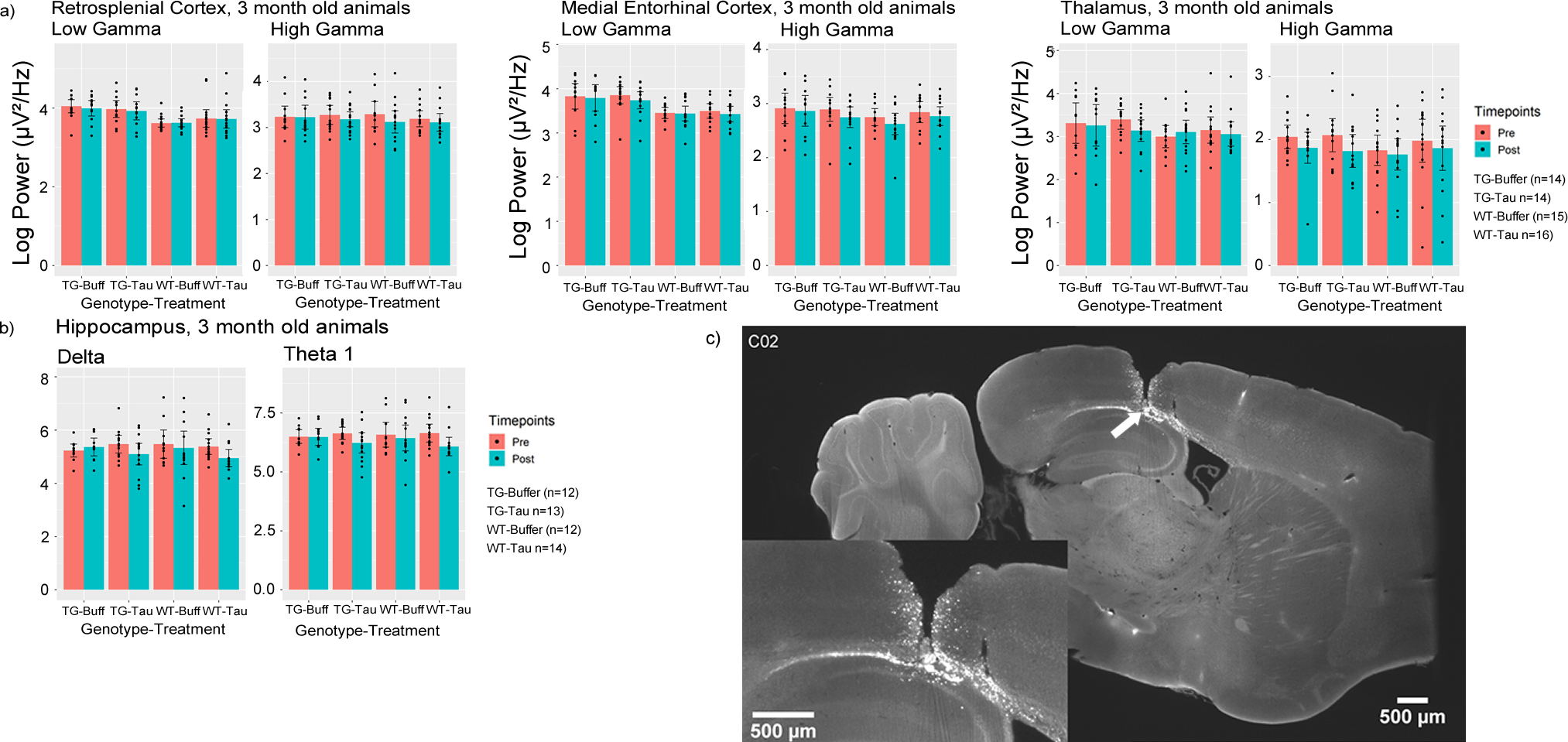

Supplement: Supplementary file 3 — Supplementary Information 3. [file 41598_2022_11582_MOESM3_ESM.png]

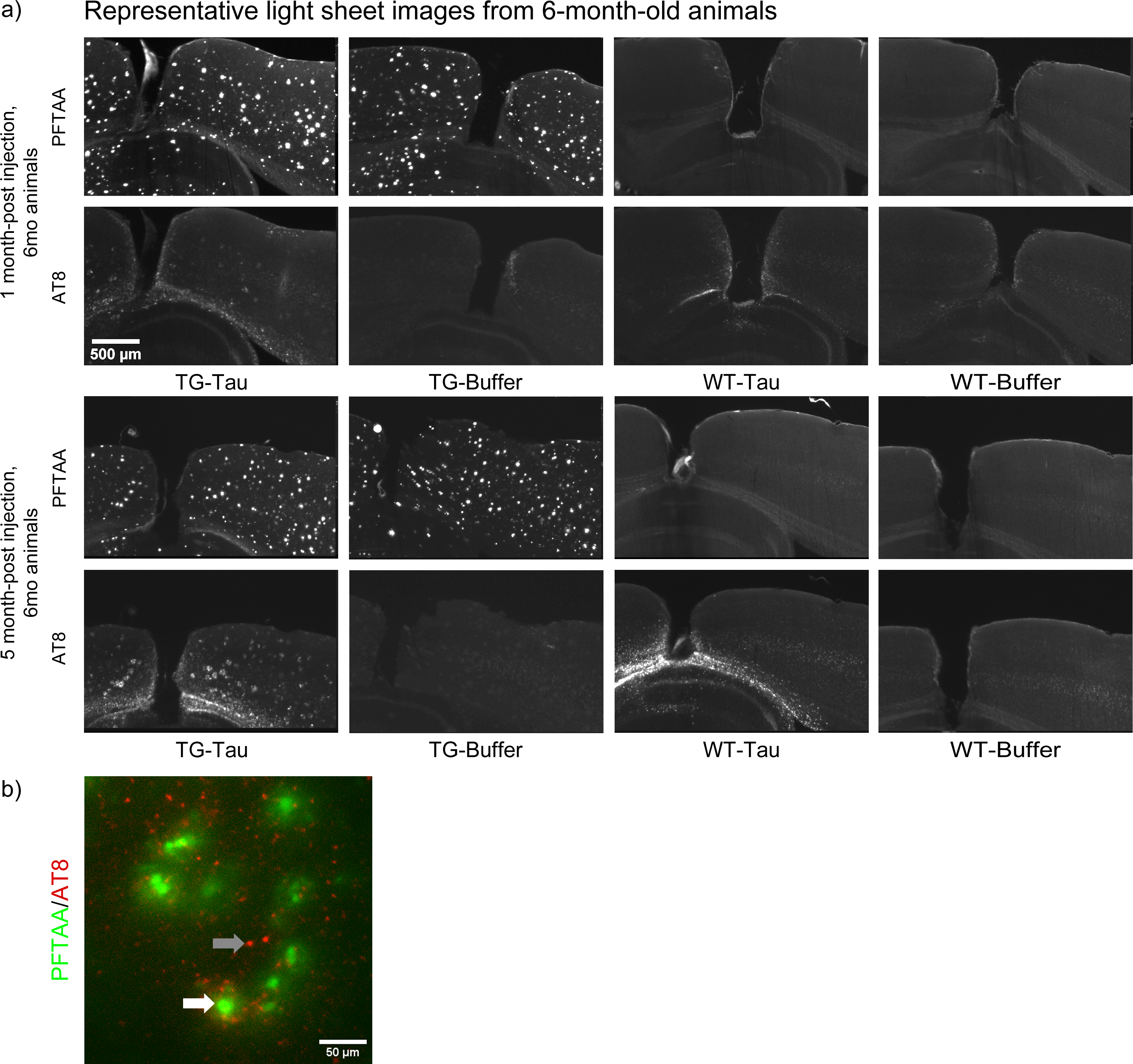

Supplement: Supplementary file 4 — Supplementary Information 4. [file 41598_2022_11582_MOESM4_ESM.png]
