# Supplementary material for: Neurophysiological effects of human-derived pathological tau conformers in the APPKM670/671NL.PS1/L166P amyloid mouse model of Alzheimer’s disease
Source: Sci Rep. 2022 May 11;12:7784. doi: 10.1038/s41598-022-11582-1 (PMC9094605; doi:10.1038/s41598-022-11582-1)
Supplement: Supplementary file 5 — Supplementary Information 5. [file 41598_2022_11582_MOESM5_ESM.docx]

Supplementary information

# Supplementary Methods

## M1. Electrolesion parameters and settings

The stimulation for electrolesion was a rectangular AC pulse signal with a duty cycle of 25%, at a current of ±250µA for 150 cycles at 2Hz. The stimulator ground wire was connected to ground electrode of the animal and stimulation wire was connected to each recording and reference electrode on the animal.

## M2. DeepLabCut model construction and activity level estimation

Video data obtained during recording was cropped and downscaled using ffmpeg version 4.2 via a custom MATLAB script. Videos were cropped to a relative rectangular frame that fit the dimensions of the home cage in the recording. These videos were subsequently resized to 50% of the cropped width and height.

Analysis of the videos was carried out using DeepLabCut to estimate the position of body parts of the animal, and subsequently, the activity of the animal. A DeepLabCut model was manually trained to track the head, body and base of the tail using 25 different animals across multiple recording boxes in order to build a generalizable neural network model. 20 still frames from each video recording for a total of 500 frames were extracted using the kmeans algorithm for frame selection and body parts of the animal were manually labelled by the experimenter where possible. If the animal was not visible due to visual obstruction by the environment, the respective tracking point was removed from that frame. The base neural network model was selected as resnet_50 and was trained using the default augmentation method and frame dataset specified above. The model was trained for up to 500000 iterations, or until the loss plateaued. Once the network was trained, the network was evaluated on naïve video data not used in the training or testing process to evaluate the generalizability of the network and detect tracking errors. Outlier frames were extracted to retrain the model for 2 more iterations using the jump algorithm specified in the software package.

The position data from the animal was generated for each frame of the video and motion data was calculated from frame position data interpolation. Motion data was smoothed across 25 frames using mean averaging, and additional movement artefacts were detected using a standard deviation filter of 5 SD and removed by interpolating the position data. The classification of animal activity was a binary state of 0 (inactive) or 1 (active) with an activity threshold calculated from 30% of the peak motion displacement over the entire recording session

## M3. Artefact and noise removal algorithms and specific exclusion criteria.

Local field potential data acquired from recordings was 1Hz high-pass filtered using a 2^nd^ order Butterworth zero-phase IIR software filter constructed using the Signal Processing Toolbox in MATLAB 2016a. Signal quality was inspected at multiple levels to remove noise and artefacts by excluding animals that did not meet the quality criteria. Signals were visually inspected for the presence of 50Hz noise, in one (indicative of a broken electrode) or multiple recording channels (indicative of multiple damaged electrodes or broken ground electrode) and electrodes were excluded from subsequent analysis. The presence of high-amplitude low frequency signals slightly above 1Hz correlated with the movement of the animal was indicative of a broken reference electrode and recordings containing that artefact were excluded from the analysis. Subsequent artefact detection involved the removal of non-physiological high-amplitude spikes that had similar amplitudes across all channels using a standard deviation cutoff of 10 SD using a custom in-house MATLAB script. Epochs The final quality check was carried out using histological verification of electrode positions in the brain regions via the electro-lesion procedure described above. Electrode locations were checked in a subset of animals to verify the accuracy of electrodes.

## M4. General linear mixed model and statistical analysis.

A general linear mixed model (GLMM) was fit to the amount of AT8, amyloid or colocalized pathology with Sex, Age at injection, Genotype, Treatment (seeded vs. buffer), Time post injection and Brain region as fixed effects (main effects), and the Age x Genotype x Treatment x Time post injection x Brain region as interaction term (including all lower-order interactions that constitute this 5th-order interaction). A random intercept was included for each animal. A backward stepwise elimination model building procedure was used in which a series of likelihood ratio tests were conducted to evaluate the statistical significance of the fixed effects (starting with the highest-order terms in a hierarchical way). The asymptotic null distribution of the likelihood ratio test is a $\chi^{2}$ with degrees of freedom equal to the difference in the number of fixed-effect parameters in the models that are being compared (Verbeke & Molenberghs, 2000). An alpha-level of 0.05 was used throughout the analyses, using Benjamini-Hochberg false discovery rate to account for multiple testing. Marginal residuals were visually inspected to check the normality assumption, and homoscedasticity was evaluated based on graphical inspection of the marginal residuals against the predicted values. There were no major violation of the model assumptions.

The dependent variables of PFTAA (i.e. amyloid pathology) and AT8 (tau pathology), or colocalized pathology (i.e. plaque-associated tau) was fit using the independent variables of gender (binary, categorical), genotype (binary, categorical), treatment (binary, categorical), brain region (nominal, categorical), time post injection (numerical, continuous) and age (numerical, continuous) as main effects and the genotype x treatment x brain region x time post injection x age interaction term (including all lower-order interaction terms). The model included a random intercept for animal. The model can be written as:

$Y_{is}=\beta_{0}+b_{i}+\beta_{1}{Gender}_{i}+\beta_{2}{Age}_{i}+\beta_{3}{Brain region}_{i}+\beta_{4}{Genotype}_{i}+\beta_{5}{Treatment}_{i}+\beta_{6}{Time}_{i}+\ldots+ \beta_{k}\left( {Age}_{i}*{Brain region}_{i}*{Genotype}_{i}*{Treatment}_{i}*{Time}_{i} \right)+\varepsilon_{is}$,

With:

$Y_{is}$= the measured amyloid or tau pathology for the *i*-th animal in the *s*-th brain region

$\beta_{0}$= the intercept

$b_{i}$= the random intercept for the *i*-th animal

$\beta_{1}$ to $\beta_{k}$ = the fixed effects for the main effects and interaction terms

Notice that shorthand notation is used to avoid a very long formula, i.e., … refers to all lower-order interaction terms of the ${Age}_{i}*{Brain region}_{i}*{Genotype}_{i}*{Treatment}_{i}*{Time}_{i}$ interaction term in the model. In addition, brain region is a categorical variable with 4 levels so it is coded using three dummy variables in the mixed model (i.e., the main effect of brain region is in fact captured by 3 fixed effects (beta’s) instead of only one as is shown in the above equation for brevity). The model assumes that the residuals and the random intercepts are normally distributed with mean zero and variance sigma, i.e., $\varepsilon_{is}\sim N(0, \sigma_{\varepsilon})$ and $b_{i}\sim N(0, \sigma_{b})$.

The following R code is used to fit the model as follows:

M1 <- lme (fixed = Pathology~SEX+Age*BrainRegion*GENOTYPE*TREAT*TP, data=Data_filt, random = ~1|MouseID, method="ML", na.action = na.omit)

Whereby Pathology refers to the quantified amount of amyloid, tau or colocalized pathology, SEX refers to the Sex of the animal, Age refers to the age of the animal when it was injected (i.e. 3 or 6 months of age), BrainRegion refers to the respective brain region (Hippocampal region, Entorhinal area, Isocortex or Thalamus), GENOTYPE refers to the genotype of the animal (TG or WT), treatment refers to the type of injection (i.e. buffer or tau-seed) and TP refers to the amount of time after injection (1, 3, or 5 months post injection). Data refers to the dataframe used for analysis (amyloid or tau data values). The random intercepts are specified with the random = ~1|MouseID option. The method for fitting the model is specified as method="ML", where ML refers to maximum likelihood. na.action = na.omit specifies that the model omits data that contains missing values.

Subsequently, the interaction effect was tested by comparing to a model that does not contain the interaction term of interest using the anova package. This is described in the code as such:

M1b<- lme(fixed= LabelRatio~SEX+TP*TREAT*GENOTYPE*LabelName+TP*TREAT*GENOTYPE*Age+TP*TREAT*LabelName*Age+TP*GENOTYPE*LabelName*Age+TREAT*GENOTYPE*LabelName*Age+TP*TREAT*GENOTYPE+TP*TREAT*LabelName+TP*GENOTYPE*LabelName+TREAT*GENOTYPE*LabelName+TP*TREAT*Age+TP*GENOTYPE*Age+TREAT*GENOTYPE*Age+TP*LabelName*Age+TREAT*LabelName*Age+GENOTYPE*LabelName*Age+TP*TREAT+TP*GENOTYPE+TREAT*GENOTYPE+TP*LabelName+TREAT*LabelName+GENOTYPE*LabelName+TP*Age+TREAT*Age+GENOTYPE*Age+LabelName*Age, data=Data _filt, random = ~1|MouseID, method="ML", na.action = na.omit)

anova(M1,M1b)

Where M1b refers to the model containing all interaction terms except for the interaction term of interest (i.e. Age*LabelName*GENOTYPE*TREAT*TP).

The anova command compared the two models and generates a likelihood ratio between the two models, as well as a p-value indicating if the two models differ significantly, and if the model containing the interaction term fits the data better.

## M5. General linear model for analysis of neurophysiological readouts

Similar to the model described in M4., the neurophysiological outcomes were evaluated using the general linear model approach. The dependent variables of power spectra (delta, theta 1, theta 2, low gamma, high gamma), phase amplitude coupling (Theta1-Low gamma, Theta2-Low gamma, Theta1-High gamma, Theta2-High gamma) and Higuchi Fractal Dimension score were fit using the using the independent variables of sex (binary, categorical), and an interaction term of genotype (binary, categorical), treatment (binary, categorical), electrode (nominal, categorical), time post injection (numerical, continuous) and age (numerical, continuous) as fixed effects. The model can be written as:

$Y_{isj}=\beta_{0}+b_{i}+\beta_{1}{Gender}_{i}+\beta_{2}{Age}_{i}+\beta_{3}{Brain region}_{i}+\beta_{4}{Genotype}_{i}+\beta_{5}{Treatment}_{i}+\beta_{6}{Time}_{i}+\ldots+ \beta_{k}\left( {Age}_{i}*{Brain region}_{i}*{Genotype}_{i}*{Treatment}_{i}*{Time}_{i} \right)+\varepsilon_{isj}$,

With:

$Y_{isj}$= the measured amyloid or tau pathology for the *i*-th animal in the *s*-th brain region of the j-th timepoint

$\beta_{0}$= the intercept

$b_{i}$= the random intercept for the *i*-th animal

$\beta_{1}$ to $\beta_{k}$ = the fixed effects for the main effects and interaction terms

Notice that shorthand notation is used to avoid a very long formula, i.e., … refers to all lower-order interaction terms of the ${Age}_{i}*{Brain region}_{i}*{Genotype}_{i}*{Treatment}_{i}*{Time}_{i}$ interaction term in the model. In addition, brain region is a categorical variable with 4 levels so it is coded using three dummy variables in the mixed model (i.e., the main effect of brain region is in fact captured by 3 fixed effects (beta’s) instead of only one as is shown in the above equation for brevity). The model assumes that the residuals and the random intercepts are normally distributed with mean zero and variance sigma, i.e., $\varepsilon_{isj}\sim N(0, \sigma_{\varepsilon})$ and $b_{i}\sim N(0, \sigma_{b})$.

The code describing the model is as follows:

M1 <- lme(fixed = THETA_1~SEX+Age*ELECTRODE*GENOTYPE*TREAT*TP, data=Data, random = ~1|ANIM_REF, method="ML", na.action = na.omit)

Whereby M1 refers to the fitted model, THETA_1 in this instance, refers to the power spectra values of the theta 1 frequency band, SEX refers to the sex of the animal, Age refers to the age of the animal when it was injected (i.e. 3 or 6 months of age), ELECTRODE refers to the respective electrode as a proxy for brain region (Hippocampal CA1 region, Entorhinal cortex, Isocortex or Thalamus), GENOTYPE refers to the genotype of the animal (TG or WT), treatment refers to the type of injection (i.e. buffer or tau-seed) and TP refers to the amount of time after injection (1, 3, or 5 months post injection). Data refers to the dataframe used for analysis (i.e. power spectra, HFD scores, phase-amplitude coupling values etc.). The random intercepts are specified with the random = ~1|ANIM_REF option. The method for fitting the model is specified as method="ML", where ML refers to maximum likelihood. na.action = na.omit specifies that the model omits data that contains missing values.

Subsequently, the interaction effect was tested by comparing to a model that does not contain the interaction term of interest using the anova package. This is described in the code as such:

M1b<-lme(fixed = THETA_1~SEX+Age*ELECTRODE*GENOTYPE*TREAT+Age*ELECTRODE*GENOTYPE*TP+Age*ELECTRODE*TREAT*TP+Age*GENOTYPE*TREAT*TP+ELECTRODE*GENOTYPE*TREAT*TP+Age*ELECTRODE*GENOTYPE+Age*ELECTRODE*TREAT+Age*ELECTRODE*TP+Age*GENOTYPE*TREAT+Age*GENOTYPE*TP+Age*TREAT*TP+ELECTRODE*GENOTYPE*TREAT+ELECTRODE*GENOTYPE*TP+ELECTRODE*TREAT*TP+GENOTYPE*TREAT*TP+Age*ELECTRODE+Age*GENOTYPE+Age*TREAT+Age*TP+ELECTRODE*GENOTYPE+ELECTRODE*TREAT+ELECTRODE*TP+GENOTYPE*TREAT+GENOTYPE*TP*+TREAT*TP,
data=Data, random = ~1|ANIM_REF, method="ML", na.action = na.omit)

anova(M1,M1b)

Where M1b refers to the model containing all interaction terms except for the interaction term of interest (i.e. Age*LabelName*GENOTYPE*TREAT*TP).

Similarly, for electrophysiological analyses general linear mixed model (GLMM) was fit to the neurophysiological readouts with Sex, Age at injection, Genotype, Treatment (seeded vs. buffer), Time post injection and Brain region as fixed effects (main effects), and the Age x Genotype x Treatment x Time post injection x Brain region as interaction term (including all lower-order interactions that constitute this 5th-order interaction).

# Supplementary Figures

## Supplementary Figure 1.


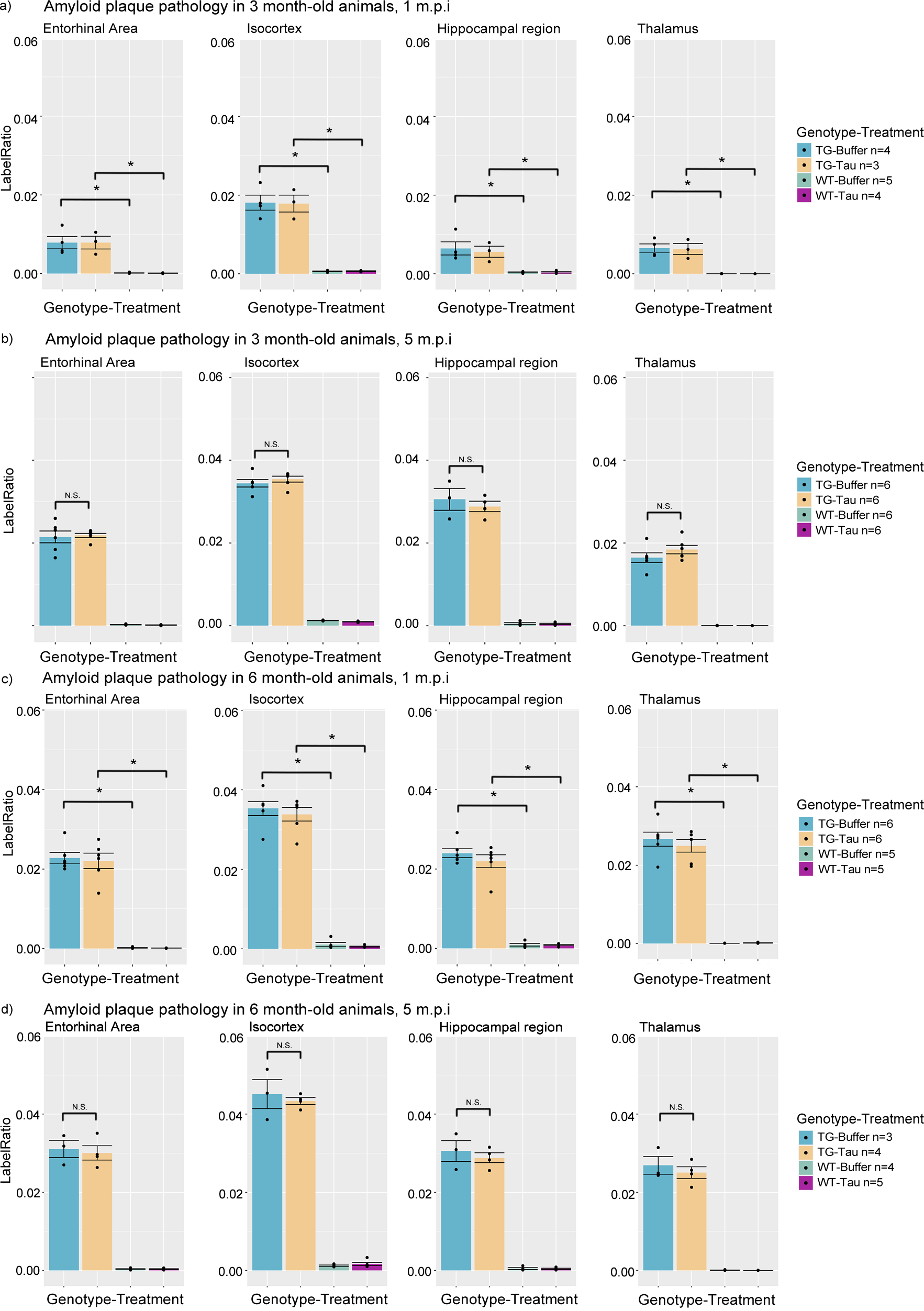


## Supplementary Figure 2.


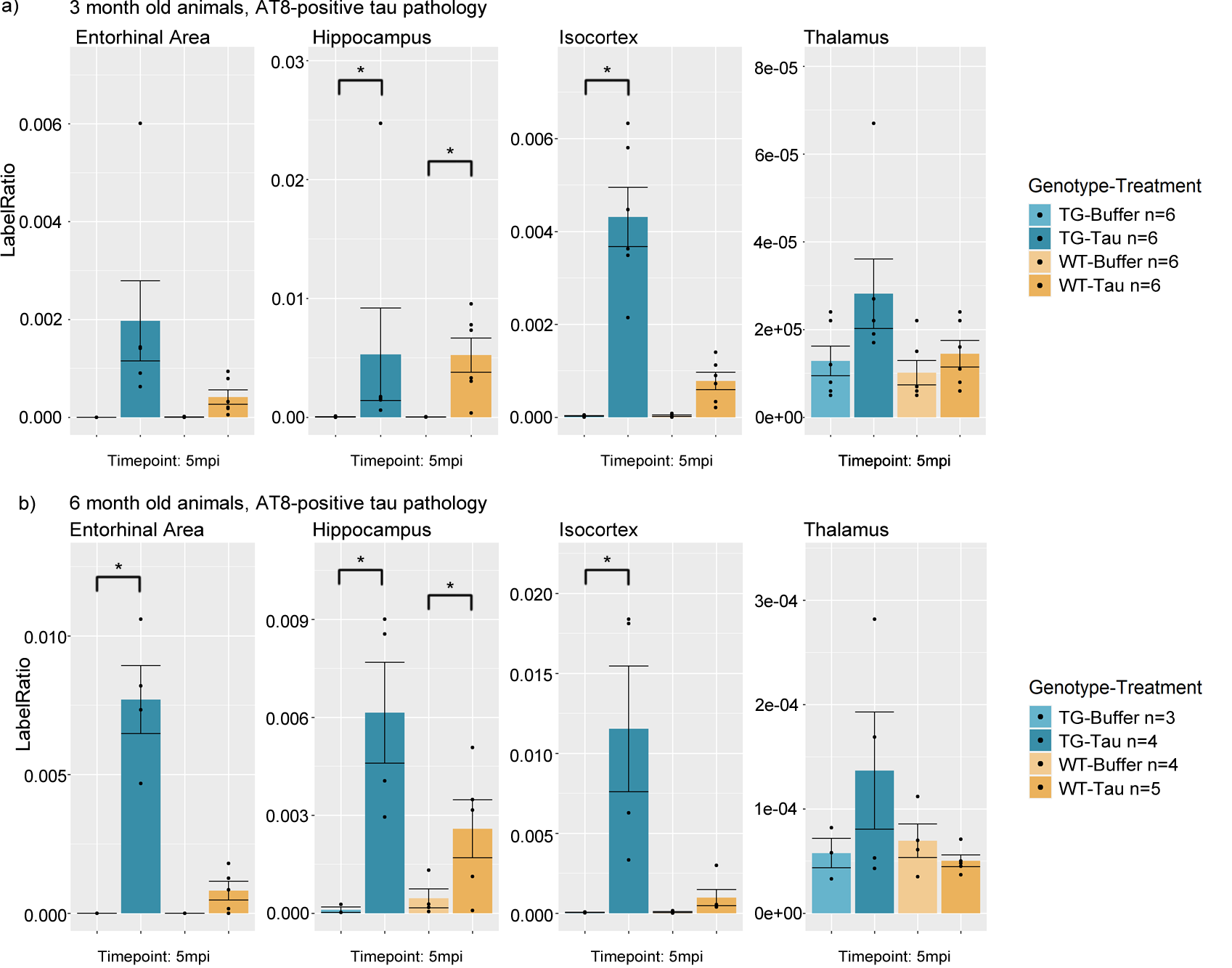


## Supplementary Figure 3.


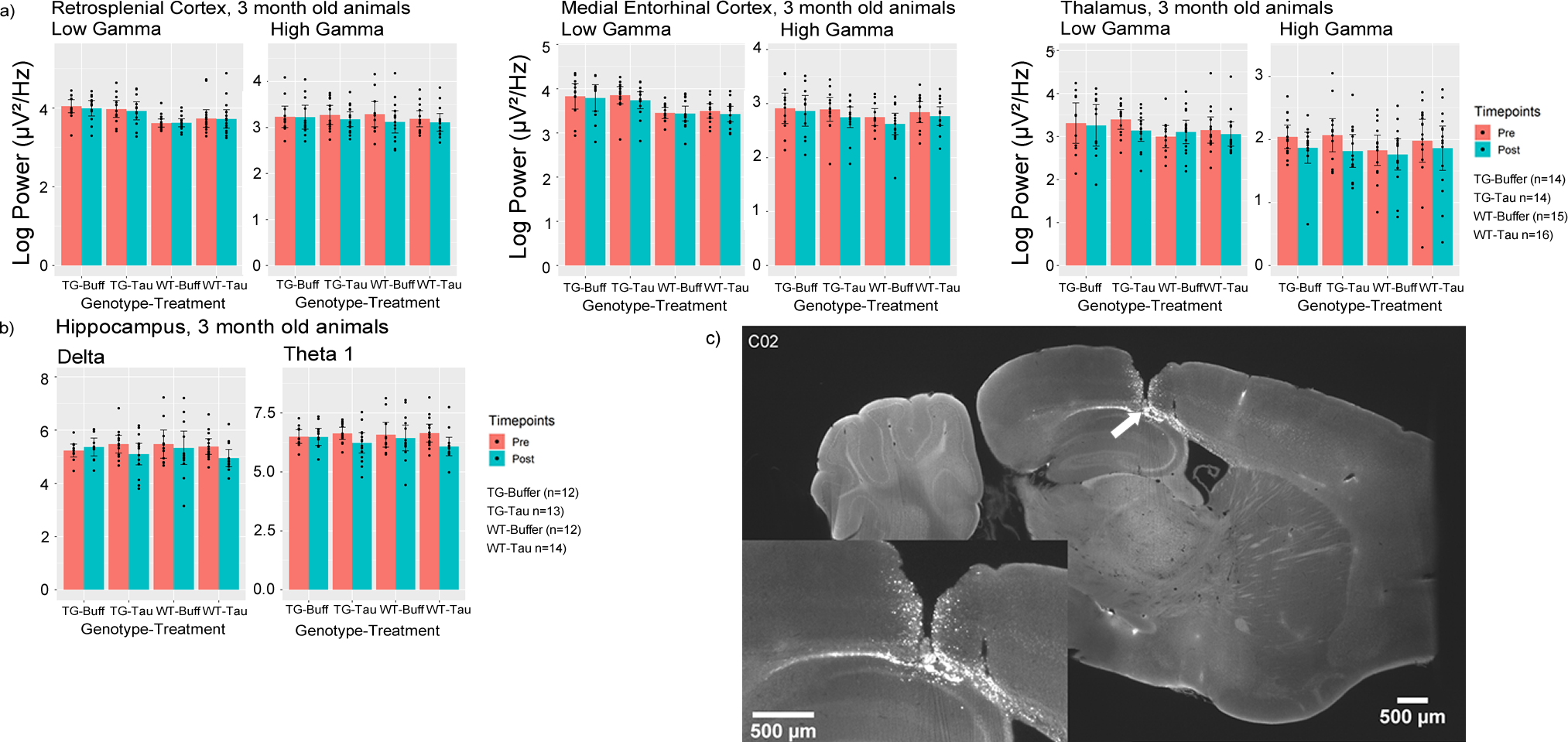


## Supplementary Figure 4.


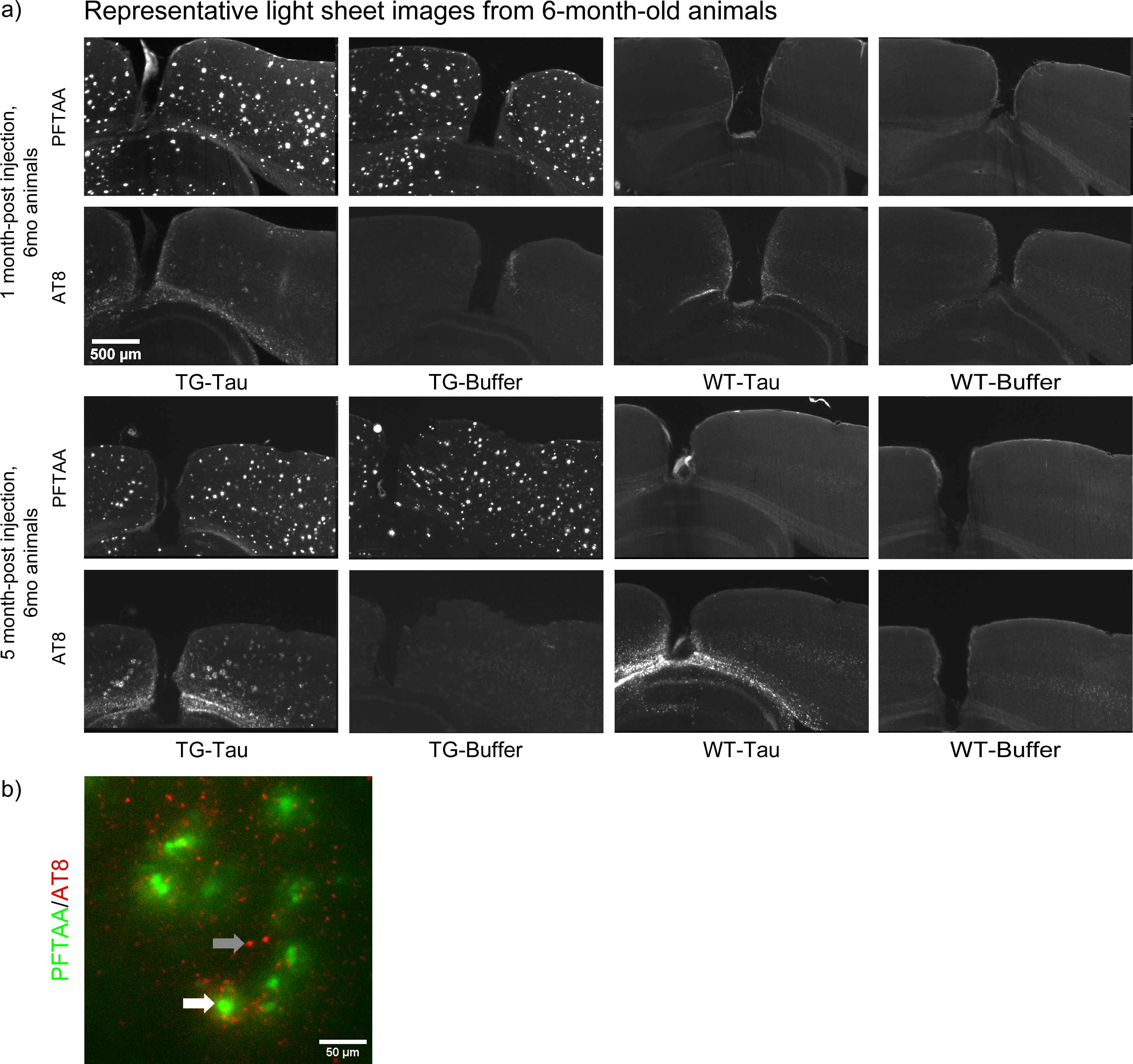


# Supplementary figure legends

## Supplementary Figure 1

Bar plots of PFTAA-positive amyloid pathology in a) 3-month-old animals and b) 6-month-old TG animals compared to WT animals demonstrating significantly increased levels of amyloid pathology in TG animals. c) Bar plots of 6-month-old animals illustrating the lack of a significant difference in the amount of amyloid pathology in TG animals injected with and without AD-tau seeds at 5 months post injection. All error bars indicate standard error of mean. Significant comparisons can be found in Supplementary Table 1. m.p.i refers to months-post-injection. All bar plots are Mean+SEM. Asterisks indicate significant comparisons (p<0.05). N.S. refers to not significant. TG refers to TG animals, WT refers to wild-type. Tau refers to tau-seeded animals and buffer refers to buffer-injected animals.

## Supplementary Figure 2

Bar plots of AT8-positive tau pathology in a) 3-month-old animals and b) 6-month-old animals comparing between AD-tau injected and non-injected animals across several brain regions. Animals injected with AD-tau seeds exhibit significantly increased AT8-positive tau pathology compared to buffer-injected animals. Significant comparisons can be found in Supplementary Table 2. All error bars indicate standard error of mean. All bar plots are Mean+SEM. m.p.i refers to months-post-injection. Asterisks indicate significant comparisons (p<0.05). TG refers to TG animals, WT refers to wild-type. Tau refers to tau-seeded animals and buffer refers to buffer-injected animals.

## Supplementary Figure 3

Bar plots of power band spectra of a) 3-month-old animals and 6-month-old animals for the power bands of Low Gamma and High Gamma in the Retrosplenial cortex, Medial Entorhinal Cortex, and Thalamus. No significant differences were noted in these brain regions. b) Bar plots of power band spectra in 3-month-old animals showing no significant impairments in other oscillatory bands in the hippocampus. All error bars indicate standard error of mean c) Representative histological image showing injection site marked with white arrow. All bar plots are Mean+SEM. TG refers to TG animals, WT refers to wild-type. Tau refers to tau-seeded animals and buffer refers to buffer-injected animals. Pre refers to values prior to injection. Post-refers to values taken one day after recording.

## Supplementary Figure 4

Representative light sheet images from 6-month-old animals as well as high magnification power light sheet image. a) Representative light sheet images from animals injected at 6-months of age, at 1 and 5 months-post injection, showing development of AT8-positive tau pathology in tau seeded mice. b) High magnification image illustrating AT8-positive tau (red signal) localized to the periphery of a plaque (green signal, white arrow), as well as non-colocalized AT8-positive tau (grey arrow).

# Supplementary Tables

## Supplementary Table 1

| 1a) Amyloid plaque pathology, Tg vs WT |  |  |  |  |  |  |
| --- | --- | --- | --- | --- | --- | --- |
| Brain region | Pairwise comparison | estimate | SE | df | T ratio | P value |
| Entorhinal area | 3mo TG Buffer 1mpi - 3mo WT Buffer 1mpi | 0.00778 | 0.00149 | 96 | 5.22940 | 1.64E-06 |
|  | 6mo TG Buffer 1mpi - 6mo WT Buffer 1mpi | 0.02273 | 0.00134 | 96 | 16.92728 | 4.99E-30 |
|  | 3mo TG Buffer 3mpi - 3mo WT Buffer 3mpi | 0.01916 | 0.00134 | 96 | 14.26643 | 4.98E-25 |
|  | 6mo TG Buffer 3mpi - 6mo WT Buffer 3mpi | 0.03179 | 0.00140 | 96 | 22.66995 | 1.82E-39 |
|  | 3mo TG Buffer 5mpi - 3mo WT Buffer 5mpi | 0.02121 | 0.00128 | 96 | 16.57262 | 2.16E-29 |
|  | 6mo TG Buffer 5mpi - 6mo WT Buffer 5mpi | 0.03049 | 0.00170 | 96 | 17.93635 | 8.15E-32 |
|  |  |  |  |  |  |  |
|  | 3mo TG Tau 1mpi - 3mo WT Tau 1mpi | 0.00763 | 0.00169 | 96 | 4.50146 | 3.02E-05 |
|  | 6mo TG Tau 1mpi - 6mo WT Tau 1mpi | 0.02186 | 0.00134 | 96 | 16.27653 | 7.48E-29 |
|  | 3mo TG Tau 3mpi - 3mo WT Tau 3mpi | 0.01730 | 0.00134 | 96 | 12.88266 | 2.92E-22 |
|  | 6mo TG Tau 3mpi - 6mo WT Tau 3mpi | 0.03081 | 0.00134 | 96 | 22.93785 | 7.47E-40 |
|  | 3mo TG Tau 5mpi - 3mo WT Tau 5mpi | 0.02169 | 0.00128 | 96 | 16.94798 | 4.59E-30 |
|  | 6mo TG Tau 5mpi - 6mo WT Tau 5mpi | 0.02984 | 0.00149 | 96 | 20.05273 | 2.23E-35 |
|  |  |  |  |  |  |  |
| Isocortex | 3mo TG Buffer 1mpi - 3mo WT Buffer 1mpi | 0.01755 | 0.00149 | 96 | 11.79355 | 4.92E-20 |
|  | 6mo TG Buffer 1mpi - 6mo WT Buffer 1mpi | 0.03437 | 0.00134 | 96 | 25.59281 | 1.64E-43 |
|  | 3mo TG Buffer 3mpi - 3mo WT Buffer 3mpi | 0.03076 | 0.00134 | 96 | 22.90414 | 8.35E-40 |
|  | 6mo TG Buffer 3mpi - 6mo WT Buffer 3mpi | 0.04482 | 0.00140 | 96 | 31.96052 | 3.18E-51 |
|  | 3mo TG Buffer 5mpi - 3mo WT Buffer 5mpi | 0.03322 | 0.00128 | 96 | 25.94844 | 5.53E-44 |
|  | 6mo TG Buffer 5mpi - 6mo WT Buffer 5mpi | 0.04361 | 0.00170 | 96 | 25.65603 | 1.36E-43 |
|  |  |  |  |  |  |  |
|  | 3mo TG Tau 1mpi - 3mo WT Tau 1mpi | 0.01706 | 0.00169 | 96 | 10.06801 | 2.02E-16 |
|  | 6mo TG Tau 1mpi - 6mo WT Tau 1mpi | 0.03322 | 0.00134 | 96 | 24.73653 | 2.27E-42 |
|  | 3mo TG Tau 3mpi - 3mo WT Tau 3mpi | 0.02934 | 0.00134 | 96 | 21.84871 | 3.11E-38 |
|  | 6mo TG Tau 3mpi - 6mo WT Tau 3mpi | 0.04355 | 0.00134 | 96 | 32.42978 | 1.02E-51 |
|  | 3mo TG Tau 5mpi - 3mo WT Tau 5mpi | 0.03451 | 0.00128 | 96 | 26.95748 | 2.96E-45 |
|  | 6mo TG Tau 5mpi - 6mo WT Tau 5mpi | 0.04183 | 0.00149 | 96 | 28.10698 | 1.19E-46 |
|  |  |  |  |  |  |  |
| Hippocampal region | 3mo TG Buffer 1mpi - 3mo WT Buffer 1mpi | 0.00613 | 0.00149 | 96 | 4.12070 | 0.000124 |
|  | 6mo TG Buffer 1mpi - 6mo WT Buffer 1mpi | 0.02317 | 0.00134 | 96 | 17.25098 | 1.32E-30 |
|  | 3mo TG Buffer 3mpi - 3mo WT Buffer 3mpi | 0.01577 | 0.00134 | 96 | 11.74464 | 6.19E-20 |
|  | 6mo TG Buffer 3mpi - 6mo WT Buffer 3mpi | 0.03136 | 0.00140 | 96 | 22.36302 | 5.21E-39 |
|  | 3mo TG Buffer 5mpi - 3mo WT Buffer 5mpi | 0.01891 | 0.00128 | 96 | 14.77430 | 5.08E-26 |
|  | 6mo TG Buffer 5mpi - 6mo WT Buffer 5mpi | 0.02979 | 0.00170 | 96 | 17.52533 | 4.3E-31 |
|  |  |  |  |  |  |  |
|  | 3mo TG Tau 1mpi - 3mo WT Tau 1mpi | 0.00513 | 0.00169 | 96 | 3.02611 | 0.004594 |
|  | 6mo TG Tau 1mpi - 6mo WT Tau 1mpi | 0.02104 | 0.00134 | 96 | 15.66539 | 1.01E-27 |
|  | 3mo TG Tau 3mpi - 3mo WT Tau 3mpi | 0.01486 | 0.00134 | 96 | 11.06779 | 1.57E-18 |
|  | 6mo TG Tau 3mpi - 6mo WT Tau 3mpi | 0.03002 | 0.00134 | 96 | 22.34900 | 5.45E-39 |
|  | 3mo TG Tau 5mpi - 3mo WT Tau 5mpi | 0.01938 | 0.00128 | 96 | 15.14081 | 1E-26 |
|  | 6mo TG Tau 5mpi - 6mo WT Tau 5mpi | 0.02850 | 0.00149 | 96 | 19.15094 | 6.84E-34 |
| Thalamus |  |  |  |  |  |  |
|  | 3mo TG Buffer 1mpi - 3mo WT Buffer 1mpi | 0.00660 | 0.00149 | 96 | 4.43883 | 3.83E-05 |
|  | 6mo TG Buffer 1mpi - 6mo WT Buffer 1mpi | 0.02669 | 0.00134 | 96 | 19.87038 | 4.45E-35 |
|  | 3mo TG Buffer 3mpi - 3mo WT Buffer 3mpi | 0.01704 | 0.00134 | 96 | 12.68829 | 7.27E-22 |
|  | 6mo TG Buffer 3mpi - 6mo WT Buffer 3mpi | 0.03348 | 0.00140 | 96 | 23.87558 | 3.42E-41 |
|  | 3mo TG Buffer 5mpi - 3mo WT Buffer 5mpi | 0.01646 | 0.00128 | 96 | 12.85580 | 3.31E-22 |
|  | 6mo TG Buffer 5mpi - 6mo WT Buffer 5mpi | 0.02651 | 0.00170 | 96 | 15.59939 | 1.34E-27 |
|  |  |  |  |  |  |  |
|  | 3mo TG Tau 1mpi - 3mo WT Tau 1mpi | 0.00613 | 0.00169 | 96 | 3.61954 | 0.000707 |
|  | 6mo TG Tau 1mpi - 6mo WT Tau 1mpi | 0.02470 | 0.00134 | 96 | 18.39390 | 1.31E-32 |
|  | 3mo TG Tau 3mpi - 3mo WT Tau 3mpi | 0.01644 | 0.00134 | 96 | 12.23958 | 5.99E-21 |
|  | 6mo TG Tau 3mpi - 6mo WT Tau 3mpi | 0.03247 | 0.00134 | 96 | 24.17980 | 1.34E-41 |
|  | 3mo TG Tau 5mpi - 3mo WT Tau 5mpi | 0.01838 | 0.00128 | 96 | 14.36130 | 3.25E-25 |
|  | 6mo TG Tau 5mpi - 6mo WT Tau 5mpi | 0.02513 | 0.00149 | 96 | 16.88310 | 5.98E-30 |
| 1b) Amyloid plaque pathology, Tg-Buffer vs Tg Tau |  |  |  |  |  |  |
| Brain region | Pairwise comparison | estimate | SE | df | T ratio | P value |
| Entorhinal area | 3mo TG Buffer 1mpi - 3mo TG Tau 1mpi | 0.00013 | 0.00169 | 96 | 0.07540 | 0.980988 |
|  | 3mo TG Buffer 3mpi - 3mo TG Tau 3mpi | 0.00201 | 0.00140 | 96 | 1.42849 | 0.216179 |
|  | 3mo TG Buffer 5mpi - 3mo TG Tau 5mpi | -0.00037 | 0.00128 | 96 | -0.28540 | 0.898515 |
|  |  |  |  |  |  |  |
|  | 6mo TG Buffer 1mpi - 6mo TG Tau 1mpi | 0.00077 | 0.00128 | 96 | 0.59931 | 0.699867 |
|  | 6mo TG Buffer 3mpi - 6mo TG Tau 3mpi | 0.00085 | 0.00134 | 96 | 0.63444 | 0.675229 |
|  | 6mo TG Buffer 5mpi - 6mo TG Tau 5mpi | 0.00074 | 0.00170 | 96 | 0.43264 | 0.812108 |
|  |  |  |  |  |  |  |
| Isocortex | 3mo TG Buffer 1mpi - 3mo TG Tau 1mpi | 0.00037 | 0.00169 | 96 | 0.21871 | 0.929227 |
|  | 3mo TG Buffer 3mpi - 3mo TG Tau 3mpi | 0.00132 | 0.00140 | 96 | 0.94145 | 0.468956 |
|  | 3mo TG Buffer 5mpi - 3mo TG Tau 5mpi | -0.00100 | 0.00128 | 96 | -0.78198 | 0.57531 |
|  |  |  |  |  |  |  |
|  | 6mo TG Buffer 1mpi - 6mo TG Tau 1mpi | 0.00150 | 0.00128 | 96 | 1.16945 | 0.335251 |
|  | 6mo TG Buffer 3mpi - 6mo TG Tau 3mpi | 0.00164 | 0.00134 | 96 | 1.22354 | 0.307366 |
|  | 6mo TG Buffer 5mpi - 6mo TG Tau 5mpi | 0.00146 | 0.00170 | 96 | 0.85988 | 0.522507 |
|  |  |  |  |  |  |  |
| Hippocampal region | 3mo TG Buffer 1mpi - 3mo TG Tau 1mpi | 0.00095 | 0.00169 | 96 | 0.55940 | 0.727074 |
|  | 3mo TG Buffer 3mpi - 3mo TG Tau 3mpi | 0.00111 | 0.00140 | 96 | 0.78718 | 0.572303 |
|  | 3mo TG Buffer 5mpi - 3mo TG Tau 5mpi | -0.00038 | 0.00128 | 96 | -0.29972 | 0.891842 |
|  |  |  |  |  |  |  |
|  | 6mo TG Buffer 1mpi - 6mo TG Tau 1mpi | 0.00202 | 0.00128 | 96 | 1.57854 | 0.163525 |
|  | 6mo TG Buffer 3mpi - 6mo TG Tau 3mpi | 0.00139 | 0.00134 | 96 | 1.03536 | 0.411233 |
|  | 6mo TG Buffer 5mpi - 6mo TG Tau 5mpi | 0.00143 | 0.00170 | 96 | 0.84106 | 0.534665 |
|  |  |  |  |  |  |  |
| Thalamus |  |  |  |  |  |  |
|  | 3mo TG Buffer 1mpi - 3mo TG Tau 1mpi | 0.00040 | 0.00169 | 96 | 0.23568 | 0.922353 |
|  | 3mo TG Buffer 3mpi - 3mo TG Tau 3mpi | 0.00058 | 0.00140 | 96 | 0.41296 | 0.825137 |
|  | 3mo TG Buffer 5mpi - 3mo TG Tau 5mpi | -0.00193 | 0.00128 | 96 | -1.50446 | 0.187975 |
|  |  |  |  |  |  |  |
|  | 6mo TG Buffer 1mpi - 6mo TG Tau 1mpi | 0.00173 | 0.00128 | 96 | 1.34939 | 0.248582 |
|  | 6mo TG Buffer 3mpi - 6mo TG Tau 3mpi | 0.00111 | 0.00134 | 96 | 0.82359 | 0.546897 |
|  | 6mo TG Buffer 5mpi - 6mo TG Tau 5mpi | 0.00155 | 0.00170 | 96 | 0.91039 | 0.489683 |
|  |  |  |  |  |  |  |

## Supplementary Table 2

| AT8-positive tau pathology, TG and WT animals |  |  |  |  |  |  |
| --- | --- | --- | --- | --- | --- | --- |
| Brain region | Pairwise comparison | estimate | SE | df | T ratio | P value |
| Entorhinal area | 3mo TG Buffer 5mpi - 3mo TG Tau 5mpi | -0.00197 | 0.00100 | 96 | -1.97151 | 1.91245E-01 |
|  | 6mo TG Buffer 5mpi - 6mo TG Tau 5mpi | -0.00771 | 0.00132 | 96 | -5.82489 | 1.13186E-06 |
|  |  |  |  |  |  |  |
|  | 3mo WT Buffer 5mpi - 3mo WT Tau 5mpi | -0.00041 | 0.00100 | 96 | -0.41175 | 9.99905E-01 |
|  | 6mo WT Buffer 5mpi - 6mo WT Tau 5mpi | -0.00081 | 0.00116 | 96 | -0.69783 | 9.99905E-01 |
|  |  |  |  |  |  |  |
| Isocortex | 3mo TG Buffer 5mpi - 3mo TG Tau 5mpi | -0.00428 | 0.00100 | 96 | -4.28673 | 3.65021E-04 |
|  | 6mo TG Buffer 5mpi - 6mo TG Tau 5mpi | -0.01148 | 0.00132 | 96 | -8.67206 | 6.77347E-12 |
|  |  |  |  |  |  |  |
|  | 3mo WT Buffer 5mpi - 3mo WT Tau 5mpi | -0.00075 | 0.00100 | 96 | -0.74961 | 9.99905E-01 |
|  | 6mo WT Buffer 5mpi - 6mo WT Tau 5mpi | -0.00091 | 0.00116 | 96 | -0.78501 | 9.99905E-01 |
|  |  |  |  |  |  |  |
| Thalamus | No significant differences |  |  |  |  |  |
| Hippocampal region | 3mo TG Buffer 5mpi - 3mo TG Tau 5mpi | -0.00526 | 0.00100 | 96 | -5.26665 | 1.08958E-05 |
|  | 6mo TG Buffer 5mpi - 6mo TG Tau 5mpi | -0.00818 | 0.00098 | 96 | -8.33231 | 7.01470E-12 |
|  |  |  |  |  |  |  |
|  | 3mo WT Buffer 5mpi - 3mo WT Tau 5mpi | -0.00520 | 0.00100 | 96 | -5.20078 | 1.36293E-05 |
|  | 6mo WT Buffer 5mpi - 6mo WT Tau 5mpi | -0.00213 | 0.00116 | 96 | -1.83124 | 2.51736E-01 |
|  |  |  |  |  |  |  |

## Supplementary Table 3

| Brain Region | Treatment-Genotype | Timepoint | Mean | Std. Dev. | Min | Max |
| --- | --- | --- | --- | --- | --- | --- |
| Amyloid Pathology |  |  |  |  |  |  |
| Entorhinal area | Buffer-TG | 1mpi | 0.016826 | 0.008312 | 0.005398 | 0.02916 |
| Entorhinal area | Buffer-TG | 3mpi | 0.025766 | 0.007346 | 0.014576 | 0.03665 |
| Entorhinal area | Buffer-TG | 5mpi | 0.024682 | 0.005887 | 0.016414 | 0.034498 |
| Entorhinal area | Buffer-WT | 1mpi | 0.00015 | 0.000146 | 0.000026 | 0.000467 |
| Entorhinal area | Buffer-WT | 3mpi | 0.000255 | 0.000283 | 0.000012 | 0.001034 |
| Entorhinal area | Buffer-WT | 5mpi | 0.000275 | 0.000173 | 0.000087 | 0.00067 |
| Entorhinal area | Tau-TG | 1mpi | 0.017312 | 0.008138 | 0.004917 | 0.027461 |
| Entorhinal area | Tau-TG | 3mpi | 0.02493 | 0.007571 | 0.015583 | 0.034152 |
| Entorhinal area | Tau-TG | 5mpi | 0.025123 | 0.004831 | 0.019551 | 0.035101 |
| Entorhinal area | Tau-WT | 1mpi | 0.000102 | 6.51E-05 | 0.000016 | 0.000208 |
| Entorhinal area | Tau-WT | 3mpi | 0.000234 | 0.000222 | 0.000036 | 0.000691 |
| Entorhinal area | Tau-WT | 5mpi | 0.000225 | 0.000173 | 0.000014 | 0.000622 |
| Hippocampal region | Buffer-TG | 1mpi | 0.016942 | 0.009493 | 0.004004 | 0.029138 |
| Hippocampal region | Buffer-TG | 3mpi | 0.024327 | 0.00897 | 0.011999 | 0.035049 |
| Hippocampal region | Buffer-TG | 5mpi | 0.023034 | 0.006814 | 0.013646 | 0.034982 |
| Hippocampal region | Buffer-WT | 1mpi | 0.000614 | 0.000576 | 0.000069 | 0.002078 |
| Hippocampal region | Buffer-WT | 3mpi | 0.000705 | 0.000331 | 0.000184 | 0.001224 |
| Hippocampal region | Buffer-WT | 5mpi | 0.000398 | 0.00038 | 0.000094 | 0.001181 |
| Hippocampal region | Tau-TG | 1mpi | 0.016491 | 0.008844 | 0.003033 | 0.02537 |
| Hippocampal region | Tau-TG | 3mpi | 0.023762 | 0.008698 | 0.010939 | 0.034418 |
| Hippocampal region | Tau-TG | 5mpi | 0.023318 | 0.005137 | 0.017487 | 0.031506 |
| Hippocampal region | Tau-WT | 1mpi | 0.000609 | 0.00043 | 0.000088 | 0.001169 |
| Hippocampal region | Tau-WT | 3mpi | 0.000576 | 0.000466 | 0.000034 | 0.00147 |
| Hippocampal region | Tau-WT | 5mpi | 0.000344 | 0.000265 | 0.000036 | 0.000872 |
| Isocortex | Buffer-TG | 1mpi | 0.028441 | 0.00974 | 0.013986 | 0.041054 |
| Isocortex | Buffer-TG | 3mpi | 0.038671 | 0.008381 | 0.026914 | 0.04807 |
| Isocortex | Buffer-TG | 5mpi | 0.038009 | 0.006472 | 0.031212 | 0.051457 |
| Isocortex | Buffer-WT | 1mpi | 0.000834 | 0.000824 | 0.000319 | 0.0031 |
| Isocortex | Buffer-WT | 3mpi | 0.00081 | 0.000495 | 0.000305 | 0.00174 |
| Isocortex | Buffer-WT | 5mpi | 0.001221 | 0.000212 | 0.000906 | 0.001649 |
| Isocortex | Tau-TG | 1mpi | 0.028511 | 0.008844 | 0.013932 | 0.037118 |
| Isocortex | Tau-TG | 3mpi | 0.037815 | 0.007844 | 0.02723 | 0.047793 |
| Isocortex | Tau-TG | 5mpi | 0.038609 | 0.004399 | 0.032187 | 0.045223 |
| Isocortex | Tau-WT | 1mpi | 0.000597 | 0.000225 | 0.000373 | 0.001034 |
| Isocortex | Tau-WT | 3mpi | 0.00069 | 0.000327 | 0.000241 | 0.001256 |
| Isocortex | Tau-WT | 5mpi | 0.001256 | 0.0007 | 0.000773 | 0.003279 |
| Thalamus | Buffer-TG | 1mpi | 0.018607 | 0.010957 | 0.004633 | 0.03305 |
| Thalamus | Buffer-TG | 3mpi | 0.025407 | 0.009348 | 0.013492 | 0.03558 |
| Thalamus | Buffer-TG | 5mpi | 0.019938 | 0.005994 | 0.01228 | 0.03141 |
| Thalamus | Buffer-WT | 1mpi | 2.62E-05 | 3E-05 | 0 | 0.000101 |
| Thalamus | Buffer-WT | 3mpi | 9.88E-05 | 0.000114 | 0 | 0.000292 |
| Thalamus | Buffer-WT | 5mpi | 2.66E-05 | 5.43E-05 | 0.000001 | 0.000178 |
| Thalamus | Tau-TG | 1mpi | 0.018706 | 0.009899 | 0.003889 | 0.028594 |
| Thalamus | Tau-TG | 3mpi | 0.025265 | 0.008776 | 0.01233 | 0.034958 |
| Thalamus | Tau-TG | 5mpi | 0.021042 | 0.004252 | 0.015802 | 0.028439 |
| Thalamus | Tau-WT | 1mpi | 8.32E-05 | 0.000118 | 0 | 0.000347 |
| Thalamus | Tau-WT | 3mpi | 6.57E-05 | 7.12E-05 | 0 | 0.000192 |
| Thalamus | Tau-WT | 5mpi | 7.73E-06 | 8.57E-06 | 0.000001 | 0.00003 |
| AT8 Tau |  |  |  |  |  |  |
| Entorhinal area | Buffer-TG | 1mpi | 0.000593 | 0.000934 | 0.000002 | 0.002591 |
| Entorhinal area | Buffer-TG | 3mpi | 4.96E-05 | 8.98E-05 | 0 | 0.000291 |
| Entorhinal area | Buffer-TG | 5mpi | 1.11E-07 | 3.33E-07 | 0 | 0.000001 |
| Entorhinal area | Buffer-WT | 1mpi | 0.000473 | 0.000669 | 0 | 0.001999 |
| Entorhinal area | Buffer-WT | 3mpi | 0.000161 | 0.00038 | 0 | 0.001235 |
| Entorhinal area | Buffer-WT | 5mpi | 2.2E-06 | 5.59E-06 | 0 | 0.000018 |
| Entorhinal area | Tau-TG | 1mpi | 0.000199 | 0.00011 | 0.000041 | 0.000367 |
| Entorhinal area | Tau-TG | 3mpi | 0.002527 | 0.001786 | 0.000068 | 0.004853 |
| Entorhinal area | Tau-TG | 5mpi | 0.004265 | 0.003607 | 0.000625 | 0.010609 |
| Entorhinal area | Tau-WT | 1mpi | 0.000244 | 0.000364 | 0 | 0.000981 |
| Entorhinal area | Tau-WT | 3mpi | 0.000126 | 0.000176 | 0.000004 | 0.000564 |
| Entorhinal area | Tau-WT | 5mpi | 0.000596 | 0.000578 | 0 | 0.001794 |
| Hippocampal region | Buffer-TG | 1mpi | 0.000485 | 0.000487 | 0.000086 | 0.001266 |
| Hippocampal region | Buffer-TG | 3mpi | 0.000625 | 0.000598 | 0.000002 | 0.001613 |
| Hippocampal region | Buffer-TG | 5mpi | 5.43E-05 | 8.66E-05 | 0.000003 | 0.000269 |
| Hippocampal region | Buffer-WT | 1mpi | 0.000516 | 0.000357 | 0.000045 | 0.001236 |
| Hippocampal region | Buffer-WT | 3mpi | 0.001057 | 0.000959 | 0.000011 | 0.002804 |
| Hippocampal region | Buffer-WT | 5mpi | 0.0002 | 0.000402 | 0.000007 | 0.001317 |
| Hippocampal region | Tau-TG | 1mpi | 0.001163 | 0.001315 | 0.000186 | 0.004325 |
| Hippocampal region | Tau-TG | 3mpi | 0.006189 | 0.00367 | 0.001054 | 0.011585 |
| Hippocampal region | Tau-TG | 5mpi | 0.005631 | 0.007339 | 0.000595 | 0.024732 |
| Hippocampal region | Tau-WT | 1mpi | 0.000627 | 0.000437 | 0.000102 | 0.001381 |
| Hippocampal region | Tau-WT | 3mpi | 0.002862 | 0.003129 | 0.000444 | 0.01165 |
| Hippocampal region | Tau-WT | 5mpi | 0.004026 | 0.003105 | 0.000083 | 0.009534 |
| Isocortex | Buffer-TG | 1mpi | 0.001881 | 0.002704 | 0.00007 | 0.008329 |
| Isocortex | Buffer-TG | 3mpi | 0.000522 | 0.000545 | 0.000014 | 0.001778 |
| Isocortex | Buffer-TG | 5mpi | 3.63E-05 | 2.4E-05 | 0.000005 | 0.000083 |
| Isocortex | Buffer-WT | 1mpi | 0.001316 | 0.001341 | 0.000014 | 0.003639 |
| Isocortex | Buffer-WT | 3mpi | 0.000466 | 0.000642 | 0.00001 | 0.001945 |
| Isocortex | Buffer-WT | 5mpi | 4.69E-05 | 5.12E-05 | 0.000005 | 0.000162 |
| Isocortex | Tau-TG | 1mpi | 0.001584 | 0.001066 | 0.000402 | 0.003256 |
| Isocortex | Tau-TG | 3mpi | 0.006119 | 0.003084 | 0.000715 | 0.010634 |
| Isocortex | Tau-TG | 5mpi | 0.007199 | 0.005985 | 0.002145 | 0.018394 |
| Isocortex | Tau-WT | 1mpi | 0.000976 | 0.001033 | 0.000137 | 0.003034 |
| Isocortex | Tau-WT | 3mpi | 0.000695 | 0.000485 | 0.000157 | 0.001473 |
| Isocortex | Tau-WT | 5mpi | 0.000873 | 0.000789 | 0.000208 | 0.002994 |
| Thalamus | Buffer-TG | 1mpi | 0.000141 | 0.00016 | 0 | 0.000539 |
| Thalamus | Buffer-TG | 3mpi | 0.000117 | 0.000122 | 0.000013 | 0.000394 |
| Thalamus | Buffer-TG | 5mpi | 2.78E-05 | 2.64E-05 | 0.000005 | 0.000082 |
| Thalamus | Buffer-WT | 1mpi | 0.000126 | 0.000151 | 0 | 0.000513 |
| Thalamus | Buffer-WT | 3mpi | 0.000107 | 0.00015 | 0.000007 | 0.000411 |
| Thalamus | Buffer-WT | 5mpi | 3.39E-05 | 3.61E-05 | 0.000005 | 0.000112 |
| Thalamus | Tau-TG | 1mpi | 0.000135 | 9.88E-05 | 0.000011 | 0.00035 |
| Thalamus | Tau-TG | 3mpi | 0.000349 | 0.000385 | 0.000026 | 0.001364 |
| Thalamus | Tau-TG | 5mpi | 7.16E-05 | 8.7E-05 | 0.000017 | 0.000282 |
| Thalamus | Tau-WT | 1mpi | 9.34E-05 | 8.01E-05 | 0.000003 | 0.000228 |
| Thalamus | Tau-WT | 3mpi | 0.000145 | 0.000174 | 0.000005 | 0.000533 |
| Thalamus | Tau-WT | 5mpi | 3.07E-05 | 2.1E-05 | 0.000006 | 0.000071 |
| Colocalized Tau |  |  |  |  |  |  |
| Entorhinal area | Buffer-TG | 1mpi | 0.000218 | 0.000301 | 0.000001 | 0.000796 |
| Entorhinal area | Buffer-TG | 3mpi | 3.99E-05 | 7.26E-05 | 0 | 0.000237 |
| Entorhinal area | Buffer-TG | 5mpi | 0 | 0 | 0 | 0 |
| Entorhinal area | Buffer-WT | 1mpi | 0.000147 | 0.000191 | 0 | 0.000495 |
| Entorhinal area | Buffer-WT | 3mpi | 0.000049 | 0.000102 | 0 | 0.00026 |
| Entorhinal area | Buffer-WT | 5mpi | 0 | 0 | 0 | 0 |
| Entorhinal area | Tau-TG | 1mpi | 0.000134 | 8.94E-05 | 0.000032 | 0.000322 |
| Entorhinal area | Tau-TG | 3mpi | 0.002166 | 0.00158 | 0.000024 | 0.004295 |
| Entorhinal area | Tau-TG | 5mpi | 0.003719 | 0.003152 | 0.000523 | 0.00912 |
| Entorhinal area | Tau-WT | 1mpi | 5.43E-05 | 8.9E-05 | 0 | 0.000248 |
| Entorhinal area | Tau-WT | 3mpi | 1.08E-05 | 1.99E-05 | 0 | 0.000062 |
| Entorhinal area | Tau-WT | 5mpi | 8.27E-06 | 2.48E-05 | 0 | 0.000083 |
| Hippocampal region | Buffer-TG | 1mpi | 0.000101 | 0.00019 | 0.000005 | 0.000624 |
| Hippocampal region | Buffer-TG | 3mpi | 0.000133 | 0.000203 | 0 | 0.000676 |
| Hippocampal region | Buffer-TG | 5mpi | 1.28E-05 | 1.78E-05 | 0 | 0.000055 |
| Hippocampal region | Buffer-WT | 1mpi | 6.23E-05 | 9.28E-05 | 0 | 0.00032 |
| Hippocampal region | Buffer-WT | 3mpi | 7.85E-05 | 0.000148 | 0 | 0.000505 |
| Hippocampal region | Buffer-WT | 5mpi | 9.9E-06 | 1.69E-05 | 0 | 0.000053 |
| Hippocampal region | Tau-TG | 1mpi | 0.000305 | 0.000451 | 0.000022 | 0.001414 |
| Hippocampal region | Tau-TG | 3mpi | 0.00169 | 0.001206 | 0.000135 | 0.004072 |
| Hippocampal region | Tau-TG | 5mpi | 0.00137 | 0.00143 | 0.000135 | 0.004203 |
| Hippocampal region | Tau-WT | 1mpi | 8.92E-05 | 0.00012 | 0 | 0.000302 |
| Hippocampal region | Tau-WT | 3mpi | 9.41E-05 | 0.000101 | 0.000012 | 0.0003 |
| Hippocampal region | Tau-WT | 5mpi | 0.000113 | 0.000118 | 0 | 0.000317 |
| Isocortex | Buffer-TG | 1mpi | 0.000766 | 0.001156 | 0.000039 | 0.003224 |
| Isocortex | Buffer-TG | 3mpi | 0.000292 | 0.000277 | 0.000005 | 0.000837 |
| Isocortex | Buffer-TG | 5mpi | 0.000008 | 5.12E-06 | 0.000003 | 0.000018 |
| Isocortex | Buffer-WT | 1mpi | 0.000378 | 0.000406 | 0.000005 | 0.00109 |
| Isocortex | Buffer-WT | 3mpi | 8.95E-05 | 9.27E-05 | 0.000001 | 0.000295 |
| Isocortex | Buffer-WT | 5mpi | 1.18E-05 | 2.14E-05 | 0.000001 | 0.000072 |
| Isocortex | Tau-TG | 1mpi | 0.00074 | 0.000403 | 0.000297 | 0.001421 |
| Isocortex | Tau-TG | 3mpi | 0.004775 | 0.002914 | 0.000494 | 0.009225 |
| Isocortex | Tau-TG | 5mpi | 0.005215 | 0.004419 | 0.001876 | 0.015017 |
| Isocortex | Tau-WT | 1mpi | 0.000232 | 0.000281 | 0.000003 | 0.000716 |
| Isocortex | Tau-WT | 3mpi | 7.15E-05 | 5.21E-05 | 0.000005 | 0.000145 |
| Isocortex | Tau-WT | 5mpi | 7.75E-05 | 0.000233 | 0.000001 | 0.000779 |
| Thalamus | Buffer-TG | 1mpi | 3.43E-05 | 7.29E-05 | 0 | 0.000238 |
| Thalamus | Buffer-TG | 3mpi | 2.73E-05 | 3.23E-05 | 0.000001 | 0.000098 |
| Thalamus | Buffer-TG | 5mpi | 4.78E-06 | 5.91E-06 | 0 | 0.000017 |
| Thalamus | Buffer-WT | 1mpi | 9.7E-06 | 2.75E-05 | 0 | 0.000088 |
| Thalamus | Buffer-WT | 3mpi | 2.36E-06 | 4.57E-06 | 0 | 0.000014 |
| Thalamus | Buffer-WT | 5mpi | 2E-07 | 6.32E-07 | 0 | 0.000002 |
| Thalamus | Tau-TG | 1mpi | 0.000035 | 4.29E-05 | 0.000001 | 0.000133 |
| Thalamus | Tau-TG | 3mpi | 0.000115 | 0.000132 | 0.000002 | 0.000442 |
| Thalamus | Tau-TG | 5mpi | 4.78E-05 | 7.84E-05 | 0.000005 | 0.000242 |
| Thalamus | Tau-WT | 1mpi | 6.56E-06 | 1.82E-05 | 0 | 0.000055 |
| Thalamus | Tau-WT | 3mpi | 0.000021 | 5.56E-05 | 0 | 0.000184 |
| Thalamus | Tau-WT | 5mpi | 0 | 0 | 0 | 0 |
| Uncolocalized Tau |  |  |  |  |  |  |
| Entorhinal area | Buffer-TG | 1mpi | 0.000375 | 0.000637 | 0 | 0.001795 |
| Entorhinal area | Buffer-TG | 3mpi | 9.6E-06 | 1.77E-05 | 0 | 0.000055 |
| Entorhinal area | Buffer-TG | 5mpi | 1.11E-07 | 3.33E-07 | 0 | 0.000001 |
| Entorhinal area | Buffer-WT | 1mpi | 0.000326 | 0.000503 | 0 | 0.001504 |
| Entorhinal area | Buffer-WT | 3mpi | 0.000112 | 0.000292 | 0 | 0.000976 |
| Entorhinal area | Buffer-WT | 5mpi | 2.2E-06 | 5.59E-06 | 0 | 0.000018 |
| Entorhinal area | Tau-TG | 1mpi | 0.000065 | 6.66E-05 | 0.000005 | 0.000185 |
| Entorhinal area | Tau-TG | 3mpi | 0.00036 | 0.00025 | 0.000044 | 0.000681 |
| Entorhinal area | Tau-TG | 5mpi | 0.000546 | 0.000483 | 0.000097 | 0.001491 |
| Entorhinal area | Tau-WT | 1mpi | 0.000189 | 0.000325 | 0 | 0.000959 |
| Entorhinal area | Tau-WT | 3mpi | 0.000115 | 0.000175 | 0.000003 | 0.000556 |
| Entorhinal area | Tau-WT | 5mpi | 0.000588 | 0.000586 | 0 | 0.001794 |
| Hippocampal region | Buffer-TG | 1mpi | 0.000384 | 0.000375 | 0.000081 | 0.001133 |
| Hippocampal region | Buffer-TG | 3mpi | 0.000492 | 0.000537 | 0.000002 | 0.001463 |
| Hippocampal region | Buffer-TG | 5mpi | 4.16E-05 | 7.96E-05 | 0.000002 | 0.00025 |
| Hippocampal region | Buffer-WT | 1mpi | 0.000454 | 0.000295 | 0.000045 | 0.000916 |
| Hippocampal region | Buffer-WT | 3mpi | 0.000979 | 0.000946 | 0.000011 | 0.002745 |
| Hippocampal region | Buffer-WT | 5mpi | 0.000189 | 0.000403 | 0.000004 | 0.001306 |
| Hippocampal region | Tau-TG | 1mpi | 0.000858 | 0.000874 | 0.000149 | 0.002911 |
| Hippocampal region | Tau-TG | 3mpi | 0.004498 | 0.003065 | 0.000873 | 0.008897 |
| Hippocampal region | Tau-TG | 5mpi | 0.004262 | 0.006042 | 0.00046 | 0.020529 |
| Hippocampal region | Tau-WT | 1mpi | 0.000537 | 0.000338 | 0.000093 | 0.001079 |
| Hippocampal region | Tau-WT | 3mpi | 0.002768 | 0.003051 | 0.000376 | 0.011353 |
| Hippocampal region | Tau-WT | 5mpi | 0.003912 | 0.003038 | 0.000083 | 0.009284 |
| Isocortex | Buffer-TG | 1mpi | 0.001115 | 0.001749 | 0.000024 | 0.005723 |
| Isocortex | Buffer-TG | 3mpi | 0.000231 | 0.000279 | 0.000009 | 0.000941 |
| Isocortex | Buffer-TG | 5mpi | 2.86E-05 | 2.2E-05 | 0.000002 | 0.000068 |
| Isocortex | Buffer-WT | 1mpi | 0.000939 | 0.00098 | 0.00001 | 0.002549 |
| Isocortex | Buffer-WT | 3mpi | 0.000377 | 0.000556 | 0.000006 | 0.001649 |
| Isocortex | Buffer-WT | 5mpi | 3.51E-05 | 3.47E-05 | 0.000003 | 0.00009 |
| Isocortex | Tau-TG | 1mpi | 0.000844 | 0.00086 | 0.000106 | 0.00209 |
| Isocortex | Tau-TG | 3mpi | 0.001344 | 0.000765 | 0.000221 | 0.003167 |
| Isocortex | Tau-TG | 5mpi | 0.001985 | 0.002027 | 0.00027 | 0.006836 |
| Isocortex | Tau-WT | 1mpi | 0.000744 | 0.000772 | 0.000049 | 0.002318 |
| Isocortex | Tau-WT | 3mpi | 0.000623 | 0.000439 | 0.000151 | 0.001328 |
| Isocortex | Tau-WT | 5mpi | 0.000795 | 0.00059 | 0.000205 | 0.002216 |
| Thalamus | Buffer-TG | 1mpi | 0.000106 | 9.59E-05 | 0 | 0.000301 |
| Thalamus | Buffer-TG | 3mpi | 8.96E-05 | 9.17E-05 | 0.000011 | 0.000296 |
| Thalamus | Buffer-TG | 5mpi | 2.28E-05 | 2.08E-05 | 0.000003 | 0.000065 |
| Thalamus | Buffer-WT | 1mpi | 0.000117 | 0.000127 | 0 | 0.000425 |
| Thalamus | Buffer-WT | 3mpi | 0.000105 | 0.000146 | 0.000007 | 0.000397 |
| Thalamus | Buffer-WT | 5mpi | 3.37E-05 | 3.57E-05 | 0.000005 | 0.00011 |
| Thalamus | Tau-TG | 1mpi | 0.0001 | 6.24E-05 | 0.000009 | 0.000217 |
| Thalamus | Tau-TG | 3mpi | 0.000233 | 0.000275 | 0.000016 | 0.000922 |
| Thalamus | Tau-TG | 5mpi | 2.39E-05 | 1.5E-05 | 0.000007 | 0.000049 |
| Thalamus | Tau-WT | 1mpi | 8.69E-05 | 7.51E-05 | 0.000003 | 0.000225 |
| Thalamus | Tau-WT | 3mpi | 0.000124 | 0.000132 | 0.000005 | 0.000349 |
| Thalamus | Tau-WT | 5mpi | 3.06E-05 | 2.09E-05 | 0.000006 | 0.000071 |

## Supplementary Table 4

| Power spectra values | Treatment-Genotype | Timepoint | Mean | Std. Dev. | Min | Max |
| --- | --- | --- | --- | --- | --- | --- |
| Theta 1 | Buffer-TG | 1mpi | 6.2700 | 0.6667 | 4.3543 | 7.6935 |
|  | Buffer-TG | 3mpi | 6.3621 | 0.6785 | 4.1783 | 7.9341 |
|  | Buffer-TG | 5mpi | 6.2171 | 0.6744 | 3.8218 | 8.0794 |
|  | Buffer-TG | Post | 6.0785 | 0.7613 | 3.1796 | 7.4647 |
|  | Buffer-TG | Pre | 6.1325 | 0.6531 | 4.2447 | 7.4913 |
|  | Buffer-WT | 1mpi | 5.9532 | 0.8456 | 4.3390 | 8.1049 |
|  | Buffer-WT | 3mpi | 6.0429 | 0.8717 | 3.1804 | 8.1348 |
|  | Buffer-WT | 5mpi | 5.9400 | 0.7832 | 4.1469 | 7.9521 |
|  | Buffer-WT | Post | 5.9492 | 0.9111 | 4.0770 | 8.0475 |
|  | Buffer-WT | Pre | 5.7286 | 0.7752 | 4.0009 | 8.1255 |
|  | Tau-TG | 1mpi | 6.3773 | 0.7442 | 3.9051 | 8.1432 |
|  | Tau-TG | 3mpi | 6.5872 | 0.7784 | 4.1152 | 8.5865 |
|  | Tau-TG | 5mpi | 6.2160 | 0.7465 | 3.9800 | 8.3330 |
|  | Tau-TG | Post | 6.1321 | 0.7779 | 3.8592 | 7.5777 |
|  | Tau-TG | Pre | 6.2447 | 0.7186 | 4.4350 | 7.6780 |
|  | Tau-WT | 1mpi | 5.9394 | 0.7642 | 3.7882 | 7.7881 |
|  | Tau-WT | 3mpi | 6.0259 | 0.8305 | 4.0747 | 7.9278 |
|  | Tau-WT | 5mpi | 6.0355 | 0.7953 | 3.8012 | 8.0487 |
|  | Tau-WT | Post | 5.6724 | 0.7555 | 4.0657 | 8.2795 |
|  | Tau-WT | Pre | 5.8228 | 0.7455 | 4.0292 | 8.1585 |
| Theta 2 | Buffer-TG | 1mpi | 6.4107 | 0.6622 | 4.6766 | 8.2824 |
|  | Buffer-TG | 3mpi | 6.4399 | 0.6437 | 4.4931 | 7.8828 |
|  | Buffer-TG | 5mpi | 6.3352 | 0.6323 | 4.1683 | 7.8952 |
|  | Buffer-TG | Post | 6.2136 | 0.7538 | 3.4456 | 7.9085 |
|  | Buffer-TG | Pre | 6.2754 | 0.6338 | 4.4683 | 7.6352 |
|  | Buffer-WT | 1mpi | 6.1726 | 0.8512 | 4.3052 | 8.4449 |
|  | Buffer-WT | 3mpi | 6.1921 | 0.8635 | 3.2514 | 8.9288 |
|  | Buffer-WT | 5mpi | 6.1360 | 0.8010 | 4.3527 | 8.6630 |
|  | Buffer-WT | Post | 6.0334 | 0.7398 | 4.2711 | 8.0285 |
|  | Buffer-WT | Pre | 5.8951 | 0.7286 | 4.1566 | 8.1034 |
|  | Tau-TG | 1mpi | 6.5119 | 0.7421 | 4.0473 | 8.8453 |
|  | Tau-TG | 3mpi | 6.6715 | 0.7610 | 4.2851 | 8.7898 |
|  | Tau-TG | 5mpi | 6.3479 | 0.7369 | 4.1877 | 8.0867 |
|  | Tau-TG | Post | 6.2157 | 0.7464 | 4.1307 | 8.2068 |
|  | Tau-TG | Pre | 6.3830 | 0.6833 | 4.8340 | 7.7386 |
|  | Tau-WT | 1mpi | 6.1094 | 0.7982 | 4.1813 | 8.3758 |
|  | Tau-WT | 3mpi | 6.1558 | 0.8207 | 4.2563 | 8.4986 |
|  | Tau-WT | 5mpi | 6.1525 | 0.7856 | 4.1434 | 8.6467 |
|  | Tau-WT | Post | 5.8043 | 0.7206 | 4.0944 | 8.3508 |
|  | Tau-WT | Pre | 5.9797 | 0.7463 | 4.1669 | 8.9170 |
| Low Gamma | Buffer-TG | 1mpi | 4.0693 | 0.6139 | 2.0711 | 5.9924 |
|  | Buffer-TG | 3mpi | 3.9527 | 0.6068 | 1.7215 | 5.4314 |
|  | Buffer-TG | 5mpi | 3.9747 | 0.4783 | 2.4073 | 5.1015 |
|  | Buffer-TG | Post | 3.8548 | 0.7387 | 1.6919 | 5.7432 |
|  | Buffer-TG | Pre | 3.8979 | 0.6532 | 2.1353 | 5.2654 |
|  | Buffer-WT | 1mpi | 3.9246 | 0.8388 | 2.3768 | 6.6022 |
|  | Buffer-WT | 3mpi | 3.7667 | 0.8154 | 1.5460 | 6.6033 |
|  | Buffer-WT | 5mpi | 3.8278 | 0.7484 | 2.1906 | 6.2464 |
|  | Buffer-WT | Post | 3.5363 | 0.7114 | 1.5102 | 6.0877 |
|  | Buffer-WT | Pre | 3.5594 | 0.7301 | 1.5015 | 6.0490 |
|  | Tau-TG | 1mpi | 4.1211 | 0.6427 | 2.3651 | 6.3097 |
|  | Tau-TG | 3mpi | 4.0482 | 0.6216 | 2.2365 | 6.3881 |
|  | Tau-TG | 5mpi | 3.9861 | 0.5857 | 1.5282 | 5.3151 |
|  | Tau-TG | Post | 3.6942 | 0.6735 | 2.1987 | 5.7592 |
|  | Tau-TG | Pre | 3.9749 | 0.6496 | 2.6192 | 5.5014 |
|  | Tau-WT | 1mpi | 3.8821 | 0.7821 | 1.9352 | 6.2277 |
|  | Tau-WT | 3mpi | 3.7869 | 0.7684 | 1.7630 | 6.2820 |
|  | Tau-WT | 5mpi | 3.7368 | 0.6938 | 2.0757 | 6.1602 |
|  | Tau-WT | Post | 3.4446 | 0.7396 | 1.5197 | 6.0048 |
|  | Tau-WT | Pre | 3.6436 | 0.7811 | 1.4802 | 6.3580 |
| High Gamma | Buffer-TG | 1mpi | 2.9660 | 0.5147 | 1.1456 | 4.5485 |
|  | Buffer-TG | 3mpi | 2.8414 | 0.5471 | 0.8517 | 3.8512 |
|  | Buffer-TG | 5mpi | 2.9043 | 0.4416 | 1.0112 | 3.6850 |
|  | Buffer-TG | Post | 2.6939 | 0.6520 | 0.6537 | 4.0428 |
|  | Buffer-TG | Pre | 2.7493 | 0.6013 | 1.0486 | 4.0883 |
|  | Buffer-WT | 1mpi | 3.0814 | 0.6760 | 1.7095 | 5.3037 |
|  | Buffer-WT | 3mpi | 2.8641 | 0.6584 | 0.9109 | 5.1280 |
|  | Buffer-WT | 5mpi | 2.9707 | 0.5707 | 1.4872 | 4.9081 |
|  | Buffer-WT | Post | 2.5786 | 0.6578 | 0.7699 | 4.9391 |
|  | Buffer-WT | Pre | 2.6890 | 0.6572 | 0.8449 | 5.0196 |
|  | Tau-TG | 1mpi | 2.9601 | 0.5349 | 1.2802 | 4.7384 |
|  | Tau-TG | 3mpi | 2.9101 | 0.5267 | 0.8486 | 4.6076 |
|  | Tau-TG | 5mpi | 2.8747 | 0.5363 | 1.3227 | 3.8863 |
|  | Tau-TG | Post | 2.5281 | 0.6208 | 1.0567 | 3.8479 |
|  | Tau-TG | Pre | 2.8307 | 0.5597 | 1.4668 | 3.8911 |
|  | Tau-WT | 1mpi | 2.9935 | 0.6244 | 0.9685 | 4.9929 |
|  | Tau-WT | 3mpi | 2.9255 | 0.6239 | 0.7322 | 4.8734 |
|  | Tau-WT | 5mpi | 2.9216 | 0.5781 | 1.1685 | 4.7849 |
|  | Tau-WT | Post | 2.5285 | 0.6931 | 0.2973 | 4.5700 |
|  | Tau-WT | Pre | 2.7608 | 0.7047 | 0.2867 | 5.0633 |
| Phase-amplitude coupling |  |  |  |  |  |  |
| Theta 1-Low Gamma | Buffer-TG | 1mpi | 6.33E-05 | 5.51E-05 | 6.45E-06 | 4.36E-04 |
|  | Buffer-TG | 3mpi | 1.48E-04 | 7.46E-04 | 7.87E-06 | 8.61E-03 |
|  | Buffer-TG | 5mpi | 7.08E-05 | 6.52E-05 | 9.11E-06 | 4.08E-04 |
|  | Buffer-TG | Post | 6.88E-05 | 4.95E-05 | 1.25E-05 | 3.46E-04 |
|  | Buffer-TG | Pre | 6.90E-05 | 7.23E-05 | 1.28E-05 | 5.00E-04 |
|  | Buffer-WT | 1mpi | 5.90E-05 | 6.13E-05 | 1.15E-05 | 4.25E-04 |
|  | Buffer-WT | 3mpi | 5.96E-05 | 4.33E-05 | 8.62E-06 | 2.96E-04 |
|  | Buffer-WT | 5mpi | 6.24E-05 | 5.47E-05 | 5.62E-06 | 4.72E-04 |
|  | Buffer-WT | Post | 7.99E-05 | 1.08E-04 | 8.17E-06 | 7.65E-04 |
|  | Buffer-WT | Pre | 5.30E-05 | 3.96E-05 | 8.17E-06 | 2.37E-04 |
|  | Tau-TG | 1mpi | 6.32E-05 | 4.83E-05 | 1.23E-05 | 3.57E-04 |
|  | Tau-TG | 3mpi | 7.40E-05 | 7.62E-05 | 8.63E-06 | 7.27E-04 |
|  | Tau-TG | 5mpi | 1.26E-04 | 3.92E-04 | 1.00E-05 | 4.66E-03 |
|  | Tau-TG | Post | 1.07E-04 | 2.00E-04 | 9.12E-06 | 1.32E-03 |
|  | Tau-TG | Pre | 6.42E-05 | 4.38E-05 | 8.00E-06 | 2.48E-04 |
|  | Tau-WT | 1mpi | 4.88E-05 | 3.04E-05 | 8.05E-06 | 2.05E-04 |
|  | Tau-WT | 3mpi | 8.05E-05 | 1.50E-04 | 8.82E-06 | 1.19E-03 |
|  | Tau-WT | 5mpi | 8.55E-05 | 2.92E-04 | 5.73E-06 | 4.13E-03 |
|  | Tau-WT | Post | 1.37E-04 | 3.25E-04 | 6.42E-06 | 1.86E-03 |
|  | Tau-WT | Pre | 6.81E-05 | 9.30E-05 | 1.05E-05 | 6.72E-04 |
| Theta 2-Low Gamma | Buffer-TG | 1mpi | 1.42E-04 | 1.15E-04 | 1.15E-05 | 1.12E-03 |
|  | Buffer-TG | 3mpi | 1.81E-04 | 4.04E-04 | 1.07E-05 | 4.28E-03 |
|  | Buffer-TG | 5mpi | 1.27E-04 | 1.18E-04 | 7.41E-06 | 6.64E-04 |
|  | Buffer-TG | Post | 1.43E-04 | 1.01E-04 | 1.21E-05 | 4.58E-04 |
|  | Buffer-TG | Pre | 1.52E-04 | 1.16E-04 | 1.62E-05 | 6.10E-04 |
|  | Buffer-WT | 1mpi | 1.87E-04 | 1.33E-04 | 1.18E-05 | 6.29E-04 |
|  | Buffer-WT | 3mpi | 1.74E-04 | 1.36E-04 | 1.12E-05 | 1.05E-03 |
|  | Buffer-WT | 5mpi | 1.86E-04 | 1.45E-04 | 1.38E-05 | 7.98E-04 |
|  | Buffer-WT | Post | 1.79E-04 | 1.57E-04 | 1.74E-05 | 9.10E-04 |
|  | Buffer-WT | Pre | 1.70E-04 | 1.37E-04 | 1.44E-05 | 8.76E-04 |
|  | Tau-TG | 1mpi | 1.32E-04 | 1.08E-04 | 1.57E-05 | 6.79E-04 |
|  | Tau-TG | 3mpi | 1.24E-04 | 1.03E-04 | 7.71E-06 | 7.53E-04 |
|  | Tau-TG | 5mpi | 1.46E-04 | 2.36E-04 | 6.92E-06 | 2.92E-03 |
|  | Tau-TG | Post | 1.61E-04 | 1.39E-04 | 1.72E-05 | 7.00E-04 |
|  | Tau-TG | Pre | 1.60E-04 | 1.24E-04 | 1.60E-05 | 7.19E-04 |
|  | Tau-WT | 1mpi | 1.47E-04 | 1.07E-04 | 1.02E-05 | 6.37E-04 |
|  | Tau-WT | 3mpi | 1.64E-04 | 1.42E-04 | 4.05E-06 | 1.05E-03 |
|  | Tau-WT | 5mpi | 1.71E-04 | 2.00E-04 | 4.39E-06 | 1.87E-03 |
|  | Tau-WT | Post | 1.94E-04 | 2.16E-04 | 1.19E-05 | 1.19E-03 |
|  | Tau-WT | Pre | 1.89E-04 | 1.55E-04 | 1.54E-05 | 7.02E-04 |
| Theta 1-High Gamma | Buffer-TG | 1mpi | 1.23E-04 | 1.30E-04 | 9.65E-06 | 9.37E-04 |
|  | Buffer-TG | 3mpi | 1.74E-04 | 5.30E-04 | 7.52E-06 | 4.89E-03 |
|  | Buffer-TG | 5mpi | 1.19E-04 | 1.26E-04 | 1.06E-05 | 7.14E-04 |
|  | Buffer-TG | Post | 1.17E-04 | 8.89E-05 | 2.02E-05 | 5.24E-04 |
|  | Buffer-TG | Pre | 1.25E-04 | 1.16E-04 | 1.37E-05 | 7.11E-04 |
|  | Buffer-WT | 1mpi | 1.20E-04 | 1.25E-04 | 1.51E-05 | 7.89E-04 |
|  | Buffer-WT | 3mpi | 1.10E-04 | 9.74E-05 | 1.13E-05 | 7.02E-04 |
|  | Buffer-WT | 5mpi | 1.13E-04 | 1.10E-04 | 7.07E-06 | 6.54E-04 |
|  | Buffer-WT | Post | 1.27E-04 | 1.41E-04 | 1.51E-05 | 1.01E-03 |
|  | Buffer-WT | Pre | 1.04E-04 | 9.73E-05 | 1.04E-05 | 6.55E-04 |
|  | Tau-TG | 1mpi | 1.14E-04 | 1.12E-04 | 1.24E-05 | 7.13E-04 |
|  | Tau-TG | 3mpi | 1.17E-04 | 1.23E-04 | 7.23E-06 | 7.35E-04 |
|  | Tau-TG | 5mpi | 1.77E-04 | 4.34E-04 | 9.65E-06 | 5.79E-03 |
|  | Tau-TG | Post | 1.62E-04 | 2.84E-04 | 1.60E-05 | 1.93E-03 |
|  | Tau-TG | Pre | 1.10E-04 | 8.72E-05 | 1.13E-05 | 4.50E-04 |
|  | Tau-WT | 1mpi | 9.09E-05 | 7.35E-05 | 1.11E-05 | 4.38E-04 |
|  | Tau-WT | 3mpi | 1.27E-04 | 1.74E-04 | 1.21E-05 | 1.13E-03 |
|  | Tau-WT | 5mpi | 1.29E-04 | 3.18E-04 | 4.05E-06 | 3.90E-03 |
|  | Tau-WT | Post | 1.97E-04 | 4.05E-04 | 6.04E-06 | 2.53E-03 |
|  | Tau-WT | Pre | 1.26E-04 | 1.46E-04 | 1.60E-05 | 8.84E-04 |
| Theta 2-High Gamma | Buffer-TG | 1mpi | 4.45E-04 | 3.88E-04 | 2.17E-05 | 3.06E-03 |
|  | Buffer-TG | 3mpi | 3.87E-04 | 4.31E-04 | 1.13E-05 | 3.22E-03 |
|  | Buffer-TG | 5mpi | 3.31E-04 | 2.88E-04 | 1.62E-05 | 1.33E-03 |
|  | Buffer-TG | Post | 3.97E-04 | 3.17E-04 | 1.18E-05 | 1.79E-03 |
|  | Buffer-TG | Pre | 4.56E-04 | 3.71E-04 | 2.23E-05 | 1.63E-03 |
|  | Buffer-WT | 1mpi | 6.99E-04 | 6.76E-04 | 2.29E-05 | 4.14E-03 |
|  | Buffer-WT | 3mpi | 5.41E-04 | 4.99E-04 | 2.98E-05 | 2.93E-03 |
|  | Buffer-WT | 5mpi | 6.19E-04 | 6.27E-04 | 2.32E-05 | 3.72E-03 |
|  | Buffer-WT | Post | 5.13E-04 | 5.33E-04 | 1.95E-05 | 2.62E-03 |
|  | Buffer-WT | Pre | 5.66E-04 | 5.53E-04 | 3.28E-05 | 3.27E-03 |
|  | Tau-TG | 1mpi | 3.87E-04 | 3.81E-04 | 1.47E-05 | 2.25E-03 |
|  | Tau-TG | 3mpi | 2.85E-04 | 2.31E-04 | 1.11E-05 | 1.17E-03 |
|  | Tau-TG | 5mpi | 3.22E-04 | 3.39E-04 | 1.17E-05 | 3.13E-03 |
|  | Tau-TG | Post | 3.96E-04 | 4.36E-04 | 3.10E-05 | 3.07E-03 |
|  | Tau-TG | Pre | 4.38E-04 | 3.79E-04 | 2.03E-05 | 1.76E-03 |
|  | Tau-WT | 1mpi | 5.18E-04 | 5.10E-04 | 2.32E-05 | 3.41E-03 |
|  | Tau-WT | 3mpi | 4.86E-04 | 4.43E-04 | 1.43E-05 | 3.63E-03 |
|  | Tau-WT | 5mpi | 5.20E-04 | 6.41E-04 | 7.57E-06 | 4.29E-03 |
|  | Tau-WT | Post | 5.13E-04 | 5.45E-04 | 5.29E-06 | 2.96E-03 |
|  | Tau-WT | Pre | 6.42E-04 | 6.31E-04 | 1.57E-05 | 3.38E-03 |
| Higuchi Fractal Dimension |  |  |  |  |  |  |
|  | Buffer-TG | 1mpi | 1.5411 | 0.0574 | 1.3859 | 1.7032 |
|  | Buffer-TG | 3mpi | 1.5345 | 0.0625 | 1.3768 | 1.6716 |
|  | Buffer-TG | 5mpi | 1.5537 | 0.0577 | 1.3676 | 1.6916 |
|  | Buffer-TG | Post | 1.5394 | 0.0550 | 1.4115 | 1.6920 |
|  | Buffer-TG | Pre | 1.5439 | 0.0482 | 1.4188 | 1.6454 |
|  | Buffer-WT | 1mpi | 1.5918 | 0.0722 | 1.4088 | 1.8323 |
|  | Buffer-WT | 3mpi | 1.5870 | 0.0755 | 1.3710 | 1.8130 |
|  | Buffer-WT | 5mpi | 1.5959 | 0.0793 | 1.3726 | 1.8017 |
|  | Buffer-WT | Post | 1.5754 | 0.0663 | 1.3843 | 1.6967 |
|  | Buffer-WT | Pre | 1.5952 | 0.0525 | 1.4246 | 1.7064 |
|  | Tau-TG | 1mpi | 1.5285 | 0.0601 | 1.3665 | 1.6790 |
|  | Tau-TG | 3mpi | 1.5209 | 0.0725 | 1.3417 | 1.7071 |
|  | Tau-TG | 5mpi | 1.5465 | 0.0679 | 1.3163 | 1.7881 |
|  | Tau-TG | Post | 1.5280 | 0.0493 | 1.3898 | 1.6336 |
|  | Tau-TG | Pre | 1.5360 | 0.0499 | 1.4353 | 1.6393 |
|  | Tau-WT | 1mpi | 1.5940 | 0.0724 | 1.4019 | 1.7606 |
|  | Tau-WT | 3mpi | 1.5988 | 0.0761 | 1.4033 | 1.7635 |
|  | Tau-WT | 5mpi | 1.6047 | 0.0745 | 1.3775 | 1.7613 |
|  | Tau-WT | Post | 1.5959 | 0.0554 | 1.4134 | 1.7516 |
|  | Tau-WT | Pre | 1.5945 | 0.0609 | 1.4163 | 1.7263 |

## Supplementary Table 5

| Patient Data |  |  |  |  |  |  |  |  |  |  |  |  |  |
| --- | --- | --- | --- | --- | --- | --- | --- | --- | --- | --- | --- | --- | --- |
| Patient ID | Age at Death | PMI | Sex | Clinical Diagnosis | Race | Ethnicity | Global Age Onset | APOE | MF Tau | MF Thio Plaques | MF Antibody Plaques | MF aSyn | MF TDP43 |
| BE077883 | 72 | 12 | Female | FTLD-NOS | White | Non-Latino | 58 | E3/E3 | 2+ | 2+ | 3+ | 0 | 0 |
| BE077881 | 71 | 4.5 | Female | PPA (Logopenic) | White | Non-Latino | 62 | E2/E4 | 3+ | 3+ |  | 0 | 0 |
| BE077890 | 79 | 4 | Female | Alzheimer's Disease Probable | Black | Non-Latino | 69 | E3/E4 | 3+ | 2+ | 3+ | 0 | 0 |
| BE077885 | 73 | 4 | Female | PPA (Semantic dementia) | White | Non-Latino | 59 | E3/E3 | 3+ | 3+ |  | 0 | 0 |
| BE077889 | 78 | 10 | Female | Alzheimer's Disease Probable | Black | Non-Latino | 62 | E4/E4 | 3+ | 3+ |  |  |  |
| BE077886 | 74 | 4 | Female | Alzheimer's Disease Probable | White | Non-Latino | 62 | E4/E4 | 3+ | 2+ | 3+ | 1+ | 0 |
| Sample Data |  |  |  |  |  |  |  |  |  |  |  |  |  |
| Patient sample | Protein concentration (mg/ml) |  |  |  |  |  |  |  |  |  |  |  |  |
| BE077883 | 1.760893575 |  |  |  |  |  |  |  |  |  |  |  |  |
| BE077881 | 1.977165486 |  |  |  |  |  |  |  |  |  |  |  |  |
| BE077890 | 2.112351699 |  |  |  |  |  |  |  |  |  |  |  |  |
| BE077889 | 5.035435237 |  |  |  |  |  |  |  |  |  |  |  |  |
| BE077889 | 4.683272218 |  |  |  |  |  |  |  |  |  |  |  |  |
| BE077886 | 3.410776788 |  |  |  |  |  |  |  |  |  |  |  |  |
| Pooled Sample | 2.572078482 |  |  |  |  |  |  |  |  |  |  |  |  |

# Supplementary Table Legends

## Supplementary Table 1 legend

Table containing the pairwise comparisons of the amount of quantified amyloid plaque pathology in TG and WT animals. Pairwise comparisons between a) transgenic APP.PS1 animals exhibiting amyloid pathology and non-transgenic wild-type animals exhibiting no amyloid pathology. b) Comparisons between tau-seeded APP.PS1 transgenic animals and wild-type animals showing a lack of an effect of tau-seeding on amyloid pathology. (m.p.i refers to months post injection). TG refers to APP.PS1 animals, WT refers to wild-type. Tau refers to tau-seeded mice, Buffer refers to phosphate-buffered saline injected mice. SE refers to standard error. df refers to degrees of freedom. Estimate refers to the estimated difference in value between pairwise comparisons. mo refers to months-old at injection.

## Supplementary Table 2 legend

Table containing the pairwise comparisons of the amount of quantified AT8-positive tau pathology in TG animals injected with tau seeds or buffer solution. Pairwise comparisons between a) transgenic APP.PS1 animals injected with buffer or tau seeds, showing a significant increase in AT8-tau pathology in tau-seeded animals. (m.p.i refers to months post injection). TG refers to APP.PS1 animals, WT refers to wild-type. Tau refers to tau-seeded mice, Buffer refers to phosphate-buffered saline injected mice. SE refers to standard error. df refers to degrees of freedom. Estimate refers to the estimated difference in value between pairwise comparisons. mo refers to months-old at injection.

## Supplementary Table 3 legend

Table containing the descriptive summary statistics of histological quantification outcomes of Amyloid pathology, AT8 tau pathology, Colocalized Tau and Uncolocalized tau pathology. Descriptive statistics of Mean, Standard deviation, Minimum and Maximum ranges for each treatment-genotype group for each timepoint. TG refers to APP.PS1 animals, WT refers to wild-type. Tau refers to tau-seeded mice, Buffer refers to phosphate-buffered saline injected mice.

## Supplementary Table 4 legend

Table containing the descriptive summary statistics of neurophysiological outcomes of Power spectra values, Phase-amplitude coupling measures and Higuchi fractal dimension scores. Descriptive statistics of Mean, Standard deviation, Minimum and Maximum ranges for each treatment-genotype group for each timepoint. TG refers to APP.PS1 animals, WT refers to wild-type. Tau refers to tau-seeded mice, Buffer refers to phosphate-buffered saline injected mice.

## Supplementary Table 5 legend

Table containing patient information, describing the age, post-mortem interval (PMI), Sex, clinical diagnosis, Race, Ethnicity, Age of Onset, Presence of APOE mutations, presence of Tau, Thioflavin-positive plaques, Antibody-positive plaques, alpha-synuclein and TDP43. Data regarding the patient-specific total protein concentrations of the seeding material, following purification, and the protein concentration after pooling.
